# Psychometric properties of Short Form-36 Health Survey, EuroQol 5-dimensions, and Hospital Anxiety and Depression Scale in patients with chronic pain

**DOI:** 10.1097/j.pain.0000000000001700

**Published:** 2019-09-13

**Authors:** Riccardo LoMartire, Björn Olov Äng, Björn Gerdle, Linda Vixner

**Affiliations:** a Division of Physiotherapy, Department of Neurobiology, Care Sciences and Society, Karolinska Institutet, Huddinge, Sweden; b School of Education, Health and Social Studies, Dalarna University, Falun, Sweden; c Center for Clinical Research Dalarna - Uppsala University, Falun, Sweden; d Department of Medical and Health Sciences, Pain and Rehabilitation Centre, Linköping University, Linköping, Sweden

**Keywords:** Chronic pain, Construct validity, EuroQol 5-Dimensions, Factor analysis, Hospital anxiety and depression scale, Internal consistency, Item response theory, Latent variable modeling, RAND-36, Short Form-36 Health Survey, Structural equation modeling, Structural validity

## Abstract

Recent research has highlighted a need for the psychometric evaluation of instruments targeting core domains of the pain experience in chronic pain populations. In this study, the measurement properties of Short Form-36 Health Survey (SF-36),EuroQol 5-dimensions (EQ-5D) and Hospital Anxiety and Depression Scale (HADS) were analyzed within the item response-theory framework based on data from 35,908 patients. To assess the structural validity of these instruments, the empirical representations of several conceptually substantiated latent structures were compared in a cross-validation procedure. The most structurally sound representations were selected from each questionnaire and their internal consistency reliability computed as a summary of their precision. Finally, questionnaire scores were correlated with each other to evaluate their convergent and discriminant validity. Our results supported that SF-36 is an acceptable measure of 2 independent constructs of physical and mental health. By contrast, although the approach to summarize the health-related quality of life construct of EQ-5D as a unidimensional score was valid, its low reliability rendered practical model implementation of doubtful utility. Finally, rather than being separated into 2 subscales of anxiety and depression, HADS was a valid and reliable measure of overall emotional distress. In support of convergent and discriminant validity, correlations between questionnaires showed that theoretically similar traits were highly associated, whereas unrelated traits were not. Our models can be applied to score SF-36 and HADS in chronic pain patients, but we recommend against using the EQ-5D model due to its low reliability. These results are useful for researchers and clinicians involved in chronic pain populations because questionnaires' properties determine their discriminating ability in patient status assessment.

## Abstract 2

Supplemental Digital Content is Available in the Text.

This large-sample item response theory-based evaluation assessed the measurement properties of SF-36, EQ-5D, and hospital anxiety and depression scale for chronic pain patients in clinical settings.

## 1. Introduction

Chronic pain is a globally prevalent condition that can permeate all aspects of a person's life.^55,61^ Because it manifests itself differently in each individual, it is also notoriously difficult to measure. Considerable resources have therefore been invested into isolating core domains of the chronic pain experience.^16,24,35,64^ Health-related quality of life (HRQoL) and emotional distress are 2 central domains that consistently recur in some form; they reflect perceived functioning and well-being in physical, mental, and social dimensions of health, and feelings of depression, anxiety, and anger, respectively. Both are recommended as core outcome domains in pain intervention clinical trials to increase research consistency.^16,35,64^ Emotional distress has additionally been considered a critical psychosocial component in chronic pain patient profiling,^35^ and was recently included as a central feature of the chronic pain experience in *ICD-11*.^41,62^

Unlike objective characteristics such as weight and height, human experiences are unobservable latent traits that need to be inferred from indicators through statistical procedures.^4^ Self-administered questionnaires are the most commonly used indicators, with the Short Form-36 Health Survey (SF-36), the EuroQol 5-Dimensions (EQ-5D) for HRQoL,^7,33,66^ and the Hospital Anxiety and Depression Scale (HADS) for emotional distress being the most predominant.^70^ The measurement properties of these questionnaires have been extensively studied in various populations, including the general population, and patients with mental health conditions, cardiovascular disease, and cancer.^6,19,21^ The combined evidence reveals that their properties vary widely in different populations,^6,19^ and recent research has highlighted the paucity of psychometric evaluations of these instruments in chronic pain patients.^15,17^

This is an important void to fill because the accuracy with which a latent trait is captured depends on a questionnaire's validity.^57^ For a questionnaire to be valid, it needs to be both based on solid underlying theory and have statistically robust empirical properties.^52,57^ Rather than being an inherent questionnaire property, validity is a property of the test score, which, in turn, hinges on the population characteristics and the applied setting.^57^ To avoid biased conclusions about the latent trait status, it is therefore critical to establish validity for individual populations separately.^57^ Hence, psychometrically sound instruments are a prerequisite for the latent trait to be captured accurately, and the properties of SF-36, EQ-5D, and HADS have not yet been systematically addressed in chronic pain patients. We therefore evaluated their measurement properties in a large sample of this population.

## 2. Methods

### 2.1. Design and participants

This psychometric study was based on data from the Swedish Quality Registry of Pain Rehabilitation, which is an extensive nationwide database of patients with chronic musculoskeletal pain who are eligible for specialist rehabilitation.^43^ It consists of self-report cross-sectional data from 35,908 patients who were consecutively recruited between 2009 and 2016 from 38 interdisciplinary pain specialist treatment clinics, distributed according to Swedish population density. These clinics target patients with particularly complex chronic pain conditions, characterized by psychological comorbidities, impaired work, and social ability, and a failure to respond to monodisciplinary interventions. Inclusion criteria were: aged 18 years or older with noncancer musculoskeletal pain for a minimum of 90 days. During their first visit, patients received information about the registry and signed a written informed consent form. They then provided information on demographics and pain characteristics, and completed the Swedish versions of HADS, EQ-5D, and SF-36 in this order. This study was approved by Uppsala's Medical Research Ethics Committee (DNR 2018/036).

### 2.2. Questionnaires

English translations of the questionnaires are provided in the supplementary materials (available at http://links.lww.com/PAIN/A877), along with path diagrams of their conceptual frameworks, which are also described below.

#### 2.2.1. Short Form-36 Health Survey

SF-36 was designed to measure physical and mental health based on 8 health concepts: physical and social functioning, role limitations due to physical and emotional problems, mental health, vitality, bodily pain, and general health perception.^33,58,66,67^ The scale was constructed to be suitable for use by anyone, irrespective of demographics or disease, and contains 36 items that are rated on 2 to 6 ordered categories.^67^ SF-36 is often considered a measure of HRQoL,^15,17,33^ due to the definitions of health and HRQoL being inconsistent but largely overlapping.^6,37^ SF-36 is conceptualized as a hierarchical 2-level structure where the 2 constructs of physical and mental health (component summary scores), mediated through the 8 health concepts (subscales), drive the item responses.^33,65,67^ Although this theory is largely consistent across studies, its empirical representations vary, which has resulted in various scoring methods and disparate association schemes linking the constructs, subscales, and items together.^33,63,66,68^

#### 2.2.2. EuroQol 5-Dimensions

EQ-5D is a standard instrument in health economic evaluations that was constructed as a generic multiattribute utility measure to reflect the multidimensionality of HRQoL.^7,25^ It contains 5 items with 3 ordered response categories each, which were selected to target different HRQoL dimensions. The original theory defined the items as independent causes of HRQoL and summarized them as a preference-based index.^7^ However, because the items represent different aspects of HRQoL, they could simultaneously be conceptualized as indicators of a unidimensional construct.

#### 2.2.3. Hospital Anxiety and Depression Scale

The Hospital Anxiety and Depression Scale is a 14-item questionnaire, rated on a 4-point ordinal scale, which was designed to measure emotional distress in nonpsychiatric patient populations.^70^ The original theory defined 2 constructs of anxiety and depression represented by 7 items each;^70^ however, alternative structures have since been proposed and evaluated in various populations.^2,19,42^ These include a single distress construct,^47^ variations of the 2 constructs of anxiety and depression,^40^ 3 constructs of anxiety and depression combined with restlessness,^5,10^ agitation,^28^ or negative affectivity,^23^ and bifactor structures with a general factor of emotional distress and 2 or 3 specific factors for residual item dependencies.^42^

### 2.3. Structural models

**Figure 1.**
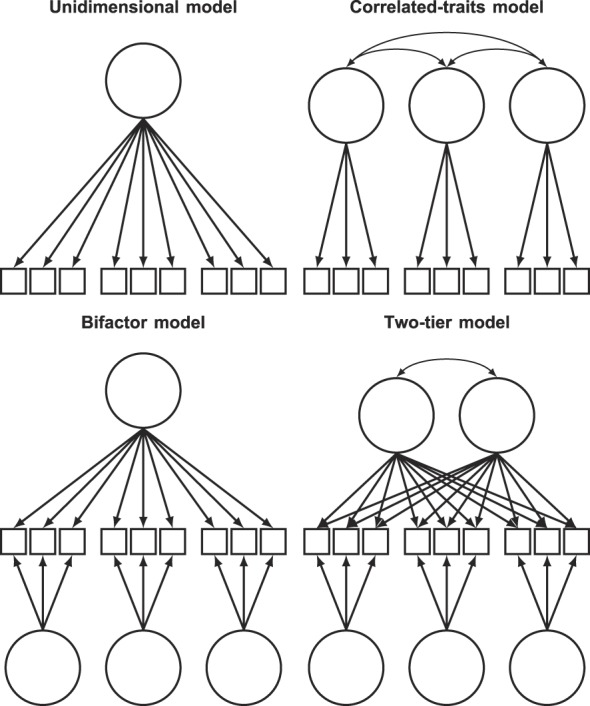
Path diagrams representing the structural models used in this study. Factors are represented by circles, items by squares, and causal pathways by arrows. Unidimensional model: all items load on one single factor that accounts for their covariance. Correlated-traits model: item subsets load on separate factors that are correlated; accounts for between-item multidimensionality. Bifactor model: each item loads on 2 uncorrelated factors; one general for all items and one specific for item subgroups; accommodates within-item multidimensionality. Two-tier model: a bifactor model with multiple general factors, which can be correlated.

Structural models are empirical representations of a questionnaire's underlying theory that specify the number of latent traits and their relationship to each other as well as to the questionnaire items (Fig. 1).^48,52^ The simplest structure is the unidimensional model, where all items measure a single latent trait. However, multidimensional models are required for questionnaires that measure more than one latent trait and can manage both between- and within-item multidimensionality. The correlated-traits model accommodates between-item multidimensionality and is implemented when subsets of items reflect different traits, which, in turn, are correlated with each other. By contrast, the two-tier model manages within-item multidimensionality in situations where items simultaneously measure some general traits of interest and specific features unique to item subsets, where the specific features can be thought of as residual dependencies not captured by the general trait.^12^ The two-tier model simplifies to both the bifactor model, when a single general trait is measured,^30^ and to the more widespread second-order model (not shown in Fig. 1), when items are constrained so that the general and specific loadings are proportional to each other.^53^

### 2.4. Functional models

Functional models describe how the latent trait and items relate to each other mathematically.^52^ Item response theory (IRT) consists of a set of such generalized models that were developed for categorical data; they subclassify according to the mathematical function they are based on. Conceptually, IRT considers the item responses to be caused by (reflect) the latent trait. Item response theory–based analyses describe the shape of the item–trait relationship by estimating the probability of observed response patterns across the latent trait. Two common IRT models are the logistic graded response model and its more constrained form, the 2-parameter logistic model; they are used for ordered and dichotomous item responses, respectively.^52,54^ Item response theory models are advantageous in psychometric evaluations due to their flexibility and the detailed information they provide on item characteristics.^52,69^ However, to be valid, they require that several assumptions are met: that the items are independent after accounting for the latent trait, that the item–trait relationship follows the specified mathematical function, and generally that the latent trait follows a predefined probability distribution.

### 2.5. Statistical analyses

Statistical analyses were computed in R (v3.5.2, R Core Team 2018) using the package “mirt” (v1.30).^14^ Four types of structural models were used to accommodate the underlying theories of the questionnaires (Fig. 1), and IRT-based generalizations of the logistic graded response model (or the 2-parameter logistic model for the dichotomous items of SF-36) were used to describe the functional relationship between latent traits and item responses.^38,48,52,54^ In confirmatory IRT models, the number of latent factors, which factors items can load on, and whether factors may intercorrelate are determined a priori, whereas the item parameters are estimated from the data. Models with normally distributed traits were estimated by full-information marginal maximum likelihood, using Bock and Aiken's expectation maximization algorithm and Cai's Metropolis-Hastings Robbins-Monro algorithm for up to 3, and 4 or more integral dimensions, respectively.^3,11^ Model assumptions of the trait's normal distribution, item independence given the latent structure, and the item–trait relationship shape following the graded response model were assessed by: visual inspection of the test score and IRT score distribution, the item residuals, and comparing the observed and expected probabilities, respectively.^39,52^ Path diagrams, assumption plots, the R scripts used in the analyses, and the models for scoring the questionnaires are provided in the supplementary materials (available at http://links.lww.com/PAIN/A877).

Patients with complete missing data were excluded from the analyses. For each questionnaire, k-fold cross-validation was computed to identify the model with the best fit and to assess the robustness of that model's parameter estimates.^32^ Specifically, data were randomly split into 5 equal parts, models were fitted on 4 parts (training set), and model fit was evaluated using the parameter estimates from the training set on the remaining part (validation set). Subsequently, models were refitted on the validation set using the same procedure with no additional constraints, and the training and validation set parameter estimate differences were computed. The procedure was repeated 5 times so that each part of the data was used as a validation set once.

Model selection was guided by several criteria. Firstly, after confirming model convergence, Akaike's and Schwartz's Bayesian information criteria were calculated to compare the model–model fit, with lower values supporting a better fit. Second, to evaluate the scale-level model-data fit, approximate fit indices based on the limited-information M_2_* statistic were used, as it generally is unrealistic to expect exact model-data fit in IRT applications due to the many degrees of freedom resulting from the possible response pattern combinations.^13,39,52^ The root mean square error of approximation (RMSEA) and the standardized root mean square residual (SRMSR) were the primary indicators, with estimates ≤ 0.05 considered an acceptable fit for both.^39,52^ However, to facilitate comparison with previous studies, the Tucker–Lewis index (TLI) and the comparative fit index (CFI) were also included, which indicate the model fit relative to the null model, with estimates ≥0.90 typically considered acceptable. Third, item-level model-data fit was assessed based on the residual correlation matrix, with estimates ≤0.05 considered negligible,^39,52^ and by comparing observed and expected S-X^2^-based category response probabilities.^36^ Fourth, person-level fit was assessed by the Zh-statistic, with estimates <|2.0| considered acceptable; negative and positive values suggest misfit (unpredictability) and overfit (redundancy), respectively.^50,52^ Finally, to determine whether the model warranted its concomitant increase in complexity, expected and maximum a posteriori scores (IRT scores) for up to 2 and 3 or more latent dimensions, respectively, and their standard errors were compared across models.^52^ For bifactor models, the explained common variance was also included as a measure of the general factor's strength relative to other factors, with estimates ≥0.85 suggesting that a unidimensional model would be sufficiently complex.^52^

Once the final model was selected, it was refitted on the complete data set to compute the final parameter estimates. The empirical internal consistency of each trait was also computed as a summary measure of precision,^14^ with estimates ≥0.70 generally considered acceptable.^60^ Finally, IRT scores were correlated with each other as well as the conventionally computed scores, as a measure of convergent and discriminant validity.

## 3. Results

### 3.1. Sample characteristics

Of the 35,908 patients, data from 34,910 (97.2%), 35,262 (98.2%) and 35,545 (99.0%) were used in the analyses, with partial missingness for 5703 (15.9%), 760 (2.1%), and 1946 (5.4%) of SF-36, EQ-5D, and HADS, respectively. Nearly 72% of the patients were female, the average age was 44 years, 84% had a secondary school education or higher, and 59% were actively employed. Moreover, 75% had a history of persistent or recurrent pain for 2 years or more, with the most prevalent locations after widespread pain being the lower back and the neck/shoulders, or upper extremities. Table 1 details the sample characteristics.

**Table 1:**
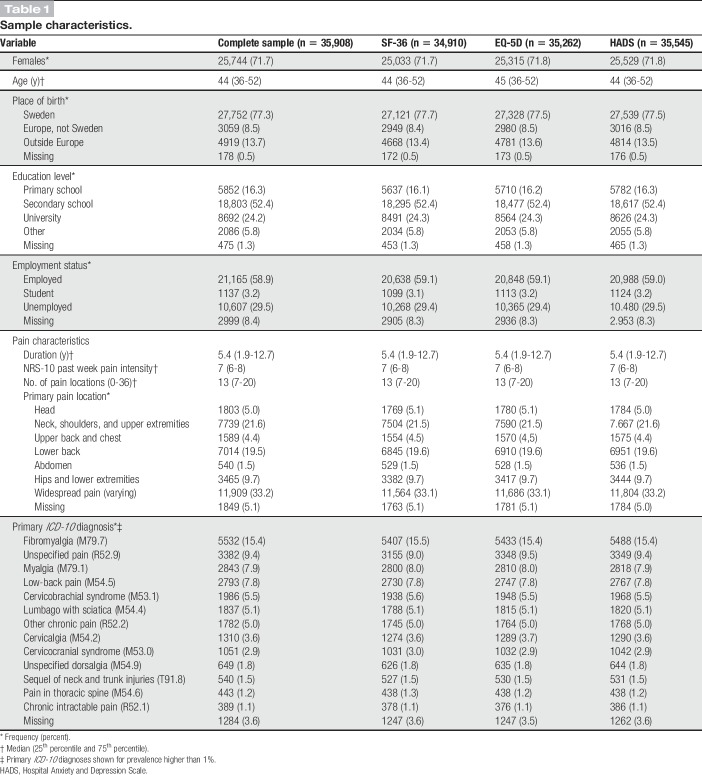
Sample characteristics.

### 3.2. Model selection

For all questionnaires, individual item distributions were either unimodal or monotone. The minimum number of observations per response category was 44 (followed by 171), 243, and 1239 for SF-36, EQ-5D, and HADS, respectively. No ceiling or floor effects were observed, with maximum and minimum test scores consistently being less than 0.1%.

Sixteen, 1, and 9 confirmatory models were computed for SF-36, EQ-5D, and HADS, respectively; all converged. For SF-36, scale-level indices supported that 5 models roughly had an acceptable fit. A two-tier model with all items free to load on 2 orthogonal general factors displayed the best fit, closely followed by its corresponding structure with 2 oblique general factors, whereas 3 bifactor models of the complete questionnaire and of the physical or mental health questionnaire sections separately had a somewhat worse fit. Piecewise indices more distinctly favored the orthogonal two-tier model because nonnegligible amounts of residual dependencies remained for the other models. However, the relationships between the general trait scores and conventional scores of all questionnaires revealed some trait–subscale inconsistencies and illogically high associations between the physical health trait and mentally oriented scales. The orthogonal two-tier model was therefore refitted with all factor loadings limited to positive values, which both mitigated the inconsistency and improved construct validity with no decrease in statistical properties. For EQ-5D, a unidimensional model was fitted, which showed satisfactory properties. Finally, indices unanimously supported that HADS was best modeled with a bifactor structure with 2 specific factors; other models performed similarly to each other, with the exception of the unidimensional model, which was markedly worse. For the selected models, latent trait distribution proxies supported normality, observed and expected categorical probabilities were similar, and item residual correlations were mostly within an acceptable level, thereby supporting the fact that the model assumptions were roughly met.

### 3.3. Short Form-36 Health Survey

Two dominant physical and mental health traits were observed, which accounted for 21.2% and 20.4% of the total variance, and constituted 30.3% and 29.3% of the variance common with the other latent traits, respectively. Scale-level indices unanimously supported an acceptable two-tier model fit {RMSEA: 0.041 (90% confidence interval [CI] 0.041-0.042); SRMSR: 0.038; TLI: 0.971; CFI: 0.976}. However, although mean item residual correlations were below 0.05, 72 individual correlations surpassed 0.05, with the maximum correlation of 0.19. Positive correlation patterns were observed both between items 24 and 25 from the mental health subscale with items 17 to 19 from the emotional role limitation subscale (*r* ≤ 0.13), and between item 22 from the bodily pain subscale with items 13 to 16 from the physical role limitation subscale (*r* ≤ 0.13). In addition, individual residual correlations remained, including between items 4 and 5 from the physical functioning subscale (*r* = 0.15), and items 23 and 27 from the vitality subscale (*r* = 0.16). Meanwhile, observed and expected response category probabilities generally matched well for most items, with the exception of items 29 and 31 of the vitality subscale, which showed distinct differences for higher categories. At person level, 4.0% (n = 1389) and 5.2% (n = 1808) of patients misfitted and overfitted the model, respectively. In addition, the cross-validation supported that most item parameters were relatively stable (loadings ≤|0.09|; intercepts ≤|0.6| logits); however, thresholds varied considerably more for items 10, 11, and 31 (≤|1.4| logits). Nonetheless, the variation in person IRT scores was less than the size of their standard errors for both traits (≤|0.3| logits).

**Figure 2.**
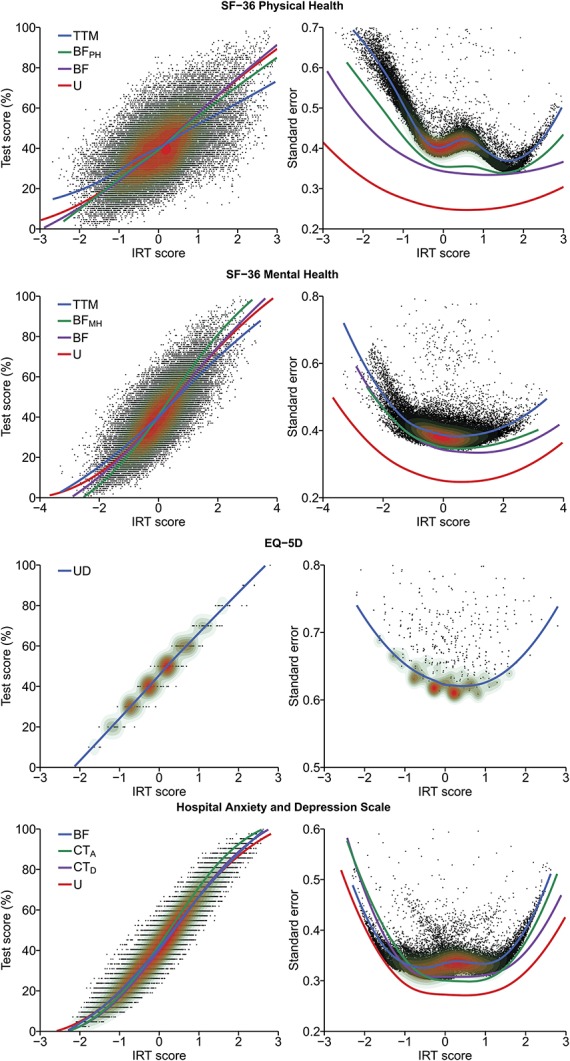
Relationship between IRT scores and standardized test scores (left), and IRT scores and their standard errors (right). The points with the overlaid contour plots show individual observations and sample density calculated from the final model, with higher density in red areas, whereas LOESS lines depict trends per model. Larger IRT scores indicate better health for SF-36, lower HRQoL for EQ-5D, and higher emotional distress for HADS. BF, bifactor model; CT, correlation traits model; HADS, Hospital Anxiety and Depression Scale; HRQoL, health-related quality of life; IRT, item response theory; TTM, two-tier model; U, unidimensional model.

The two-tier model was also motivated with respect to parsimony because meaningful differences in the IRT-test score relationship and the IRT score standard errors were observed across models (Fig. 2). Both physical health and mental health were well targeted in patients with chronic pain, and had an internal consistency of 0.79 and 0.83, respectively. Items 10 to 11 and 24 to 25 best targeted patients with low health, whereas items 14 to 16, 18, 21, and 36 were best suited for patients with high health. Physical functioning, bodily pain, and role-physical items most strongly related to the physical health trait, whereas mental health, role-emotional, social functioning, and vitality items most strongly related to the mental health trait (Table 2). In addition, physical functioning items 3 to 5 and mental health items 26 and 30 were found to be particularly pure indicators of physical and mental health, respectively.

**Table 2:**
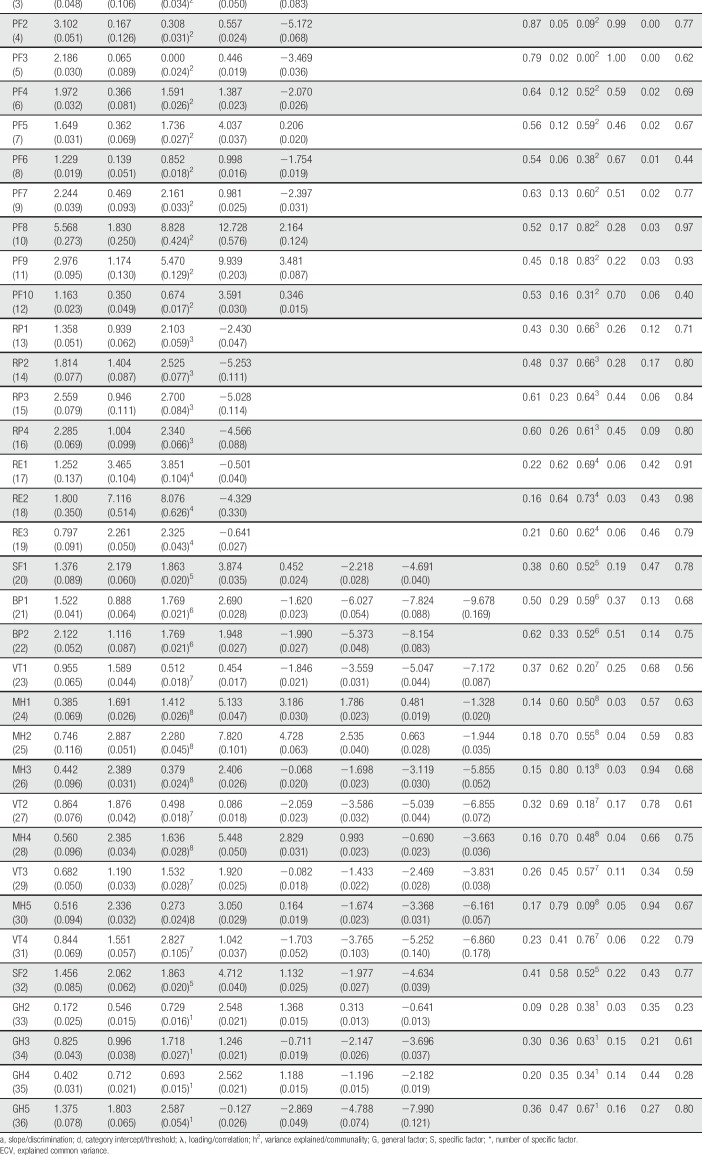
Short Form-36 Health Survey parameter estimates.

### 3.4. EuroQol 5-Dimensions

A single HRQoL trait was observed, which accounted for 35.5% of the total variance. All indices supported an acceptable unidimensional model fit, with scale-level indices within predefined thresholds (RMSEA: 0.046 [90% CI 0.040-0.052]; SRMSR: 0.031; TLI: 0.931; CFI: 0.972), item residual correlations below 0.06, observed and expected response category proportions matching well, and only 2.1% (n = 752) of patients misfitting the model. The cross-validation further sustained that item parameters (loadings ≤|0.06|; intercepts ≤|0.4| logits) and person IRT scores (≤|0.3| logits) were stable.

EQ-5D was well targeted and provided most information in the central range of the latent trait (Fig. 2); however, with an internal consistency of 0.60, its precision was rather low. Item 4 best targeted patients with high HRQoL, whereas items 1 and 2 best matched those with low HRQoL. Finally, item 2 had the strongest relationship with the HRQoL trait, whereas item 5 had the weakest (Table 3).

**Table 3:**
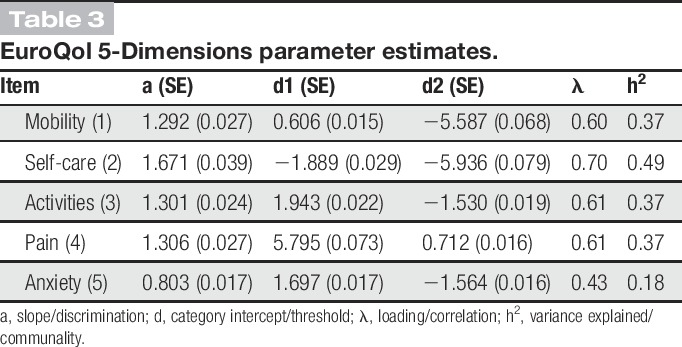
EuroQol 5-Dimensions parameter estimates.

### 3.5. Hospital Anxiety and Depression Scale

A strong emotional distress trait was identified, which represented 46.4% of the total variance, and constituted 74.1% of the variance common with the other latent traits. All indices supported an acceptable bifactor model fit, with scale-level indices within predefined thresholds (RMSEA: 0.048 [90% CI 0.046-0.049]; SRMSR: 0.032; TLI: 0.969; CFI: 0.983), and piecewise indices limited to minor misfits. At item level, mean residual correlations were below 0.05, and although 12 individual correlations surpassed that threshold, the maximum correlation was 0.09. Observed and expected response category probabilities also matched well, with deviances for items 2 and 9 in particular. At person level, 3.1% (n = 1105) misfitted the model and 1.9% (n = 678) overfitted it. The cross-validation further supported that item parameters (loadings ≤|0.06|; intercepts ≤|0.4| logits) and person IRT scores (≤|0.2| logits) were stable.

The bifactor model was also motivated with respect to parsimony because less complex models, although showing similar IRT-test score relationships, tended to overestimate IRT score precision (Fig. 2). In addition, the results showed that HADS was well targeted to the emotional distress levels in patients with chronic pain, with an internal consistency of 0.88. Items 1 and 8 best targeted patients with low emotional distress, whereas items 4 and 10 best targeted those with high emotional distress. All items were at least moderately related to the emotional distress trait, with anxiety items typically having the strongest relationship (Table 4). Finally, items 1, 7, 11, and 14 distinguished themselves as particularly pure indicators of emotional distress.

**Table 4:**
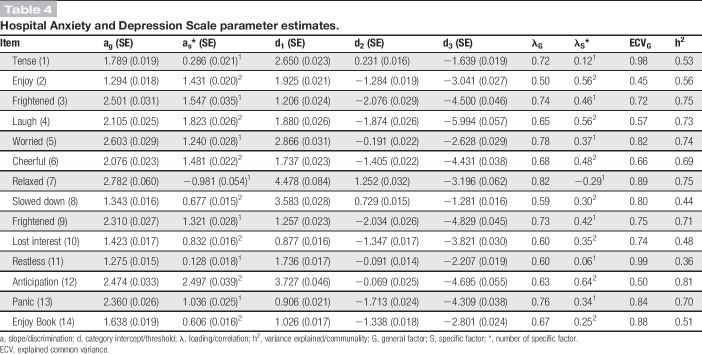
Hospital Anxiety and Depression Scale parameter estimates.

### 3.6. Latent trait relationships

**Figure 3.**
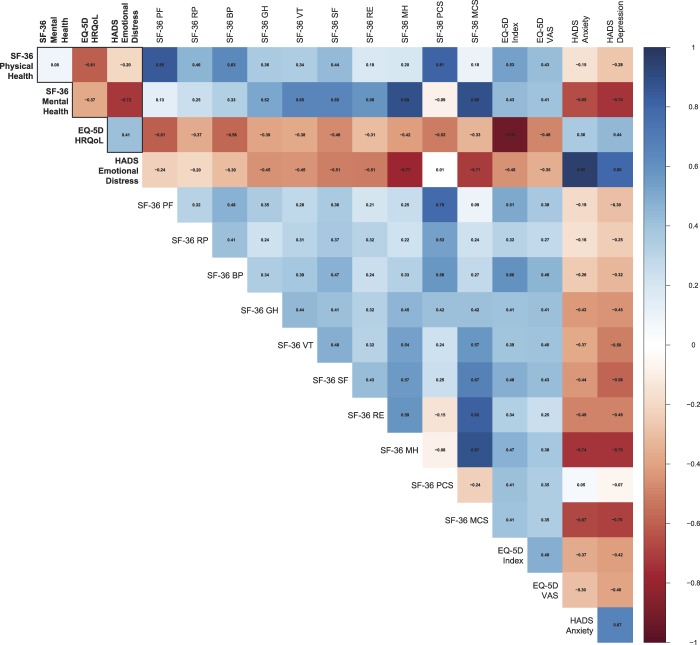
Associations between the IRT scores from the final models (bold) and the conventional scores for SF-36, EQ-5D, and HADS. Larger IRT scores indicate better health for SF-36, lower HRQoL for EQ-5D, and higher emotional distress for HADS. Conventional scores were calculated based on Ware et al., 1993, the United Kingdom time trade-off value set, and as item summaries for SF-36, EQ-5D, and HADS, respectively. BP, bodily pain; GH, general health; HADS,Hospital Anxiety and Depression Scale; HRQoL, health-related quality of life; IRT, item response theory; MH, mental health; PCS and MCS, physical and mental component summary scores, respectively; PF, physical functioning; RE, role-emotional; RP, role-physical; SF, social functioning; VT, vitality. n = 31,050.

The IRT score relationships were consistent with their underlying theories (Fig. 3). Physical health scores generally had the strongest associations with each other (SF-36 physical health vs SF-36 physical functioning subscale and SF-36 bodily pain subscale: *r* = |0.63-0.85|) and the weakest association with mental health scores (SF-36 physical health vs HADS and SF-36 mental health subscale: *r* = |0.20|). Likewise, mental health scores generally had the strongest association with each other (SF-36 mental health vs HADS and SF-36 mental health subscale: *r* = |0.72-0.89|; HADS vs SF-36 mental health subscale: *r* = −0.77), and the weakest association with physical health scores (SF mental health vs SF-36 physical functioning subscale: *r* = 0.13; HADS vs SF-36 physical functioning subscale: *r* = −0.24). The pattern was less obvious for EQ-5D, which was more equally associated to both physical (SF-36 physical health and SF-36 physical functioning subscale: *r* = |0.61|) and mental measures (SF-36 mental health, HADS, and SF-36 mental health subscale: *r* = |0.37-0.42|). Finally, all IRT scores were strongly related to their conventionally calculated scores (*r* ≥ |0.80|), most noticeably for the EQ-5D index and the HADS anxiety subscale, which had nearly perfect associations (*r* ≥ |0.92|).

## 4. Discussion

SF-36, EQ-5D, and HADS are widely used questionnaires for measuring HRQoL and emotional distress in the health sciences, and many theories pertaining to their latent structures have arisen over time. We compared the empirical representations of the most recognized theories, and evaluated their properties through a robust statistical procedure, in a large sample of chronic pain patients. For all 3 questionnaires, we identified conceptualizations that were structurally sound and logically associated, which provides evidence for their validity in the chronic pain population. However, because the internal consistency of our EQ-5D representation was unacceptably low, we recommend that our models be used to score SF-36 and HADS only.

Consistent with the original theory behind SF-36, we observed 2 general constructs of physical and mental health.^33,67^ They were most accurately captured in a model that also accounted for unrelated item dependencies within the 8 SF-36 subscales. Although these constructs are generally agreed upon,^1,26,31,33,63,65,67,68^ 2 conflicting perspectives, either viewing them as independent or correlated, have largely dominated the psychometric literature.^26,31,33,67^ Our results support the former view owing to a better data fit and higher parsimony. Summary scores based on the independent representation have received criticism for negative scoring weights that result in inconsistencies between summary scales and subscales.^59^ To a lesser extent, these concerns apply to our results because the physical and mental health scores were inversely associated with some SF-36 items. These inconsistencies were not eliminated by allowing the physical and mental traits to correlate, in line with previous findings.^26,31^ Instead, the problem was mitigated to a higher degree by restricting item loadings to positive values on all latent factors. In practice, the inconsistency is primarily a concern towards the trait score extremes and the subscale scores should therefore be considered when interpreting the overall scores in this range.^59^ However, although the selected model had the comparatively best properties of the fitted models, further refinements are likely possible. In agreement with previous reports,^1,18,26,31,65^ the physical and mental health constructs were most strongly associated with the physical functioning and mental health subscales, respectively, whereas the social functioning, vitality, and general health subscales were more evenly associated to both constructs. Largely, they were also logically correlated with other scales, particularly so between the mental health trait and HADS, which both measure mental state. However, given that the physical and mental health traits were uncorrelated by design, a small albeit seemingly contradictory association arose between the physical health construct and mentally oriented scales. A potential explanation for this is the importance of physical health perception for mental health, as previously motivated for the association between the physical health summary score and the mental health subscale of SF-36.^49^ Hence, in general agreement with most previous studies, our results support 2 summary scales and 8 subscales, but suggest that the summary scales are independent rather than correlated, which is still an open question in the scientific community.^1,26,31,33,63,65,67^

Despite the conventional conceptualization of EQ-5D as a multidimensional scale, its HRQoL construct was acceptably represented by a unidimensional model. It is justifiable to model the scale both ways from the perspective of dimensionality alone because the unidimensional model only assumes that items share one common construct, not whether systematic components unique to each item remain within the residuals.^51^ However, from a causality perspective, it may be conceptually inappropriate to model EQ-5D within the IRT framework because the original theory defines the items as independent causes of HRQoL, whereas they are viewed as correlated effects of HRQoL in IRT. Nonetheless, EQ-5D's causal structure is an open question, with recent studies providing some support for that it contains both cause and effect items.^27,29^ Our results are coherent with other IRT evaluations in chronic pain and mental illness patients, which also assessed the properties of EQ-5D as a unidimensional scale and reported acceptable unidimensionality.^34,46,56^ Our IRT-based score was strongly associated with the preference-based EQ-5D index and other physical scales and, to a lesser extent, with mental scales. These findings support construct validity and are intuitive because 4 of 5 items in EQ-5D target physical health.^7^ They are also consistent with studies of other populations, which have reported similar associations.^6,9^ Regardless, we do not recommend the use of the IRT-based score because its internal consistency was too low to reliably discriminate between patients of different HRQoL levels. This is consistent with previous results,^34,56^ and is hardly surprising because items intentionally target different HRQoL aspects. Interestingly, the multidimensional 3-level version of EQ-5D is also known to have low discriminatory power, resulting in the development of a 5-level version.^8^ Our results are limited to the former, and it is possible that an IRT score of the latter would have acceptable reliability.

Although HADS has received considerable criticism in the past,^20^ our results supported that it is a valid and reliable questionnaire. However, rather than the 2 original constructs of anxiety and depression, we observed one strong emotional distress construct. Similar to SF-36, the construct was most accurately captured in a model that accounted for unrelated item dependencies within the anxiety and depression subscales. This result corresponds to those of another study that compared the same latent structures in a meta-confirmatory factor analysis of 28 samples from various populations.^42^ Conversely, 2 studies that evaluated the dimensionality of HADS in one sample of musculoskeletal pain patients came to somewhat different conclusions, which nonetheless are supported by our findings.^44,45^ The first found that 2 highly correlated (*r* = 0.80) constructs of anxiety and depression had an acceptable fit with item 7 excluded.^44^ Similarly, we observed a nearly acceptable scale-level fit for the original 2-factor model in a sample subgroup, and item 7 also behaved differently in our analysis by loading negatively on the specific factor. Their second study suggested that HADS was sufficiently unidimensional to be used as a global measure of emotional distress, but that residual patterns related to the anxiety and depression subscales of a considerable size remained.^45^ Our findings are largely in agreement because we found additional features not accounted for by the emotional distress construct that was necessary to factor into the model. The combined evidence thereby supports our selected structure for HADS.

For all questionnaires, the latent structures selected had the best statistical properties. However, inspecting the fit of individual SF-36 items revealed that small residual dependencies, not accounted for by the model, remained. Such dependencies are common and may reflect interpretation difficulties or response bias,^1,49^ and are unlikely to implicate consequences other than a slight overestimation of the latent trait reliability. In addition to model fit, cross-validation supported that the item parameters obtained were generally stable for all questionnaires, although a more pronounced instability was observed for 3 SF-36 items, which we attribute to data sparsity owing to the many possible response patterns. Nevertheless, the IRT scores remained stable, which supports the fact that our models capture the marginal relationships in this population and that the models provided can be used to score HRQoL and emotional distress in chronic pain patients. EQ-5D was unidimensionally modeled and its scoring is thus unequivocal, but to accurately score the multidimensionally modelled traits, it is necessary to estimate them jointly with all modeled factors. Trait scores can be derived in accordance with recommended methods,^22,52^ using the scripts provided in the supplementary materials (available at http://links.lww.com/PAIN/A877). Alternatively, the models can be included directly in statistical analyses through the structural equation modeling framework.

Our results rest on a large population-representative patient sample obtained from a nearly complete database of Sweden's pain specialist treatment clinics, and should therefore be robust and generalizable. The more important limitations to consider include a possible response bias due to varying physical and social settings during data collection, and from the systematic completion of the 3 questionnaires in the same order, which partially explains the higher data attrition for the SF-36. Amounts of missing data were, however, acceptable and sensitivity analyses supported the fact that missingness did not result in any meaningful bias. Another limitation was the observational design, which necessitated the assumption of identical item–trait relationships irrespective of patient characteristics. This assumption should be tested in future measurement invariance studies. Finally, internal consistency estimates should be interpreted with caution because they summarize reliability into a single value, whereas in reality, it varies across the latent trait.

## 5. Conclusions

This study evaluated the measurement properties of SF-36, EQ-5D, and HADS for chronic pain patients in clinical settings. Our results support that SF-36 is an acceptable measure of 2 independent constructs of physical and mental health. In contrast, although it was a valid approach to summarize the HRQoL construct of EQ-5D as a unidimensional score, its low reliability rendered practical model implementation of dubious value. Finally, rather than dividing into 2 subscales of anxiety and depression, HADS was a valid and reliable measure of overall emotional distress. Relationships between the measured constructs were consistent with their underlying theories, which further supports their construct validity. We recommend that the provided models be used to score SF-36 and HADS in chronic pain patients.

## Conflict of interest statement

The authors have no conflicts of interest to declare.

## Appendix A. Supplemental digital content

Supplemental digital content associated with this article can be found online at http://links.lww.com/PAIN/A877.

## Supplementary Material

SUPPLEMENTARY MATERIAL
