# Supplementary material for: Psychometric properties of Short Form-36 Health Survey, EuroQol 5-dimensions, and Hospital Anxiety and Depression Scale in patients with chronic pain
Source: Pain. 2019 Sep 13;161(1):83–95. doi: 10.1097/j.pain.0000000000001700 (PMC6940032; doi:10.1097/j.pain.0000000000001700)
Supplement: SUPPLEMENTARY MATERIAL [file jop-161-083-s001.pdf]

Supplementary materials

Psychometric properties of SF-36, EQ-5D, and HADS
in patients with chronic pain

LoMartire R, Ang BO, Gerdle B, Vixner L

Contents

1 SF-36 3
1.1 Questionnaire . . . 3
1.2 Item response distribution . . . 6
1.3 Model path diagrams . . . 7
1.4 Model selection . . . 9
1.5 Final model . . . 10
1.5.1 Theta distribution proxies . . . 10
1.5.2 Item residual correlations . . . 11
1.5.3 Observed and expected item response proportions . . . 12
1.5.4 Marginal item category proportions . . . 21
1.5.5 Person fit . . . 22
1.6 Parameter estimate robustness . . . 23
1.6.1 Item parameters . . . 23
1.6.2 Person estimates . . . 26

2 EQ-5D 27
2.1 Questionnaire . . . 27
2.2 Item response distribution . . . 27
2.3 Model path diagram . . . 28
2.4 Model selection . . . 28
2.5 Final model . . . 29
2.5.1 Theta distribution proxies . . . 29
2.5.2 Item residual correlations . . . 29
2.5.3 Observed and expected item response proportions . . . 30
2.5.4 Marginal item category proportions . . . 30
2.5.5 Person fit . . . 31
2.6 Parameter estimate robustness . . . 32
2.6.1 Item parameters . . . 32
2.6.2 Person estimates . . . 32

3 Hospital Anxiety and Depression Scale 33
3.1 Questionnaire . . . 33
3.2 Item response distribution . . . 35
3.3 Model path diagrams . . . 36
3.4 Model selection . . . 38
3.5 Final model . . . 39
3.5.1 Theta distribution proxies . . . 39
3.5.2 Item residual correlations . . . 40
3.5.3 Observed and expected item response proportions . . . 41
3.5.4 Marginal item category proportions . . . 43
3.5.5 Person fit . . . 43
3.6 Parameter estimate robustness . . . 44
3.6.1 Item parameters . . . 44
3.6.2 Person estimates . . . 45

4 R scripts **46**
4.1 Install CRAN packages 46
4.2 Analysis scripts 46
4.3 Model specifications 49
4.4 Limited-information reproduction of the final models 53
4.5 Scoring 56

1. SF-36

1.1 Questionnaire

Table 1.1: English version of SF-36

Item	Reponse option (score)
(1) In general, would you say your health is:	Excellent (4)<br>Very good (3)<br>Good (2)<br>Fair (1)<br>Poor (0)
(2) Compared to one year ago, how would you rate your health in general now?	Much better now than one year ago (*)<br>Somewhat better now than one year ago (*)<br>About the same (*)<br>Somewhat worse now than one year ago (*)<br>Much worse now than one year ago (*)
The following items are about activities you might do during a typical day. Does your health now limit you in these activities? If so, how much?	
(3) Vigorous activities, such as running, lifting heavy objects, participating in strenuous sports	Yes, limited a lot (0)<br>Yes, limited a little (1)<br>No, not limited at all (2)
(4) Moderate activities, such as moving a table, pushing a vacuum cleaner, bowling, or playing golf	
(5) Lifting or carrying groceries	
(6) Climbing several flights of stairs	
(7) Climbing one flight of stairs	
(8) Bending, kneeling, or stooping	
(9) Walking more than a mile	
(10) Walking several blocks	
(11) Walking one block	
(12) Bathing or dressing yourself	
During the past 4 weeks, have you had any of the following problems with your work or other regular daily activities as a result of your physical health?	
(13) Cut down the amount of time you spent on work or other activities	Yes (0)<br>No (1)
(14) Accomplished less than you would like	
(15) Were limited in the kind of work or other activities	
(16) Had difficulty performing the work or other activities (for example, it took extra effort)	

Continued on next page

Table 1.1 – *Continued from previous page*

**Item**	**Reponse option (score)**
During the past 4 weeks, have you had any of the following problems with your work or other regular daily activities as a result of any emotional problems (such as feeling depressed or anxious)?	
(17) Cut down the amount of time you spent on work or other activities<br>(18) Accomplished less than you would like<br>(19) Didn't do work or other activities as carefully as usual	Yes (0)<br>No (1)
(20) During the past 4 weeks, to what extent has your physical health or emotional problems interfered with your normal social activities with family, friends, neighbors, or groups?	Not at all (4)<br>Slightly (3)<br>Moderately (2)<br>Quite a bit (1)<br>Extremely (0)
(21) How much bodily pain have you had during the past 4 weeks?	None (5)<br>Very mild (4)<br>Mild (3)<br>Moderate (2)<br>Severe (1)<br>Very severe (0)
(22) During the past 4 weeks, how much did pain interfere with your normal work (including both work outside the home and housework)?	Not at all (4)<br>A little bit (3)<br>Moderately (2)<br>Quite a bit (1)<br>Extremely (0)
These questions are about how you feel and how things have been with you during the past 4 weeks. For each question, please give the one answer that comes closest to the way you have been feeling. How much of the time during the past 4 weeks...	
(23) Did you feel full of pep?<br>(24) Have you been a very nervous person?<br>(25) Have you felt so down in the dumps that nothing could cheer you up?<br>(26) Have you felt calm and peaceful?<br>(27) Did you have a lot of energy?<br>(28) Have you felt downhearted and blue?<br>(29) Did you feel worn out?<br>(30) Have you been a happy person?<br>(31) Did you feel tired?	All of the time (0)<br>Most of the time (1)<br>A good bit of the time (2)<br>Some of the time (3)<br>A little of the time (4)<br>None of the time (5)
(32) During the past 4 weeks, how much of the time has your physical health or emotional problems interfered with your social activities (like visiting with friends, relatives, etc.)?	All of the time (0)<br>Most of the time (1)<br>Some of the time (2)<br>A little of the time (3)<br>None of the time (4)

Continued on next page

Table 1.1 – *Continued from previous page*

**Item**	**Reponse option (score)**
How TRUE or FALSE is each of the following statements for you.	
(33) I seem to get sick a little easier than other people	Definitely true (0)
(34) I am as healthy as anybody I know	Mostly true (1)
(35) I expect my health to get worse	Don't know (2)
(36) My health is excellent	Mostly false (3)
	Definitely false (4)

Adapted from *www.rand.org*.

1.2 Item response distribution

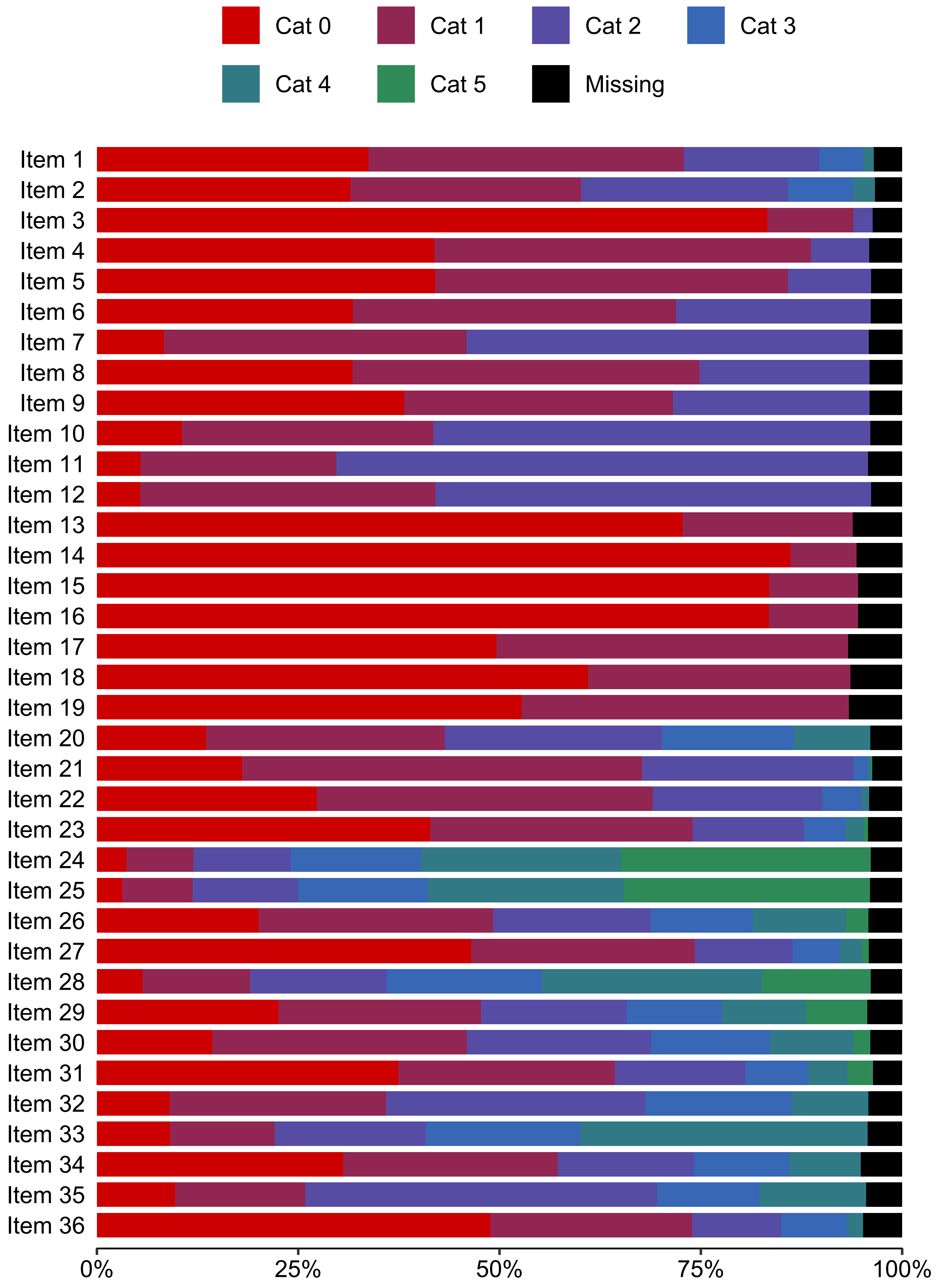

Figure 1.1: Relative item response frequencies (n = 35,908). Cat, category as defined in Table 1.1.

1.3 Model path diagrams

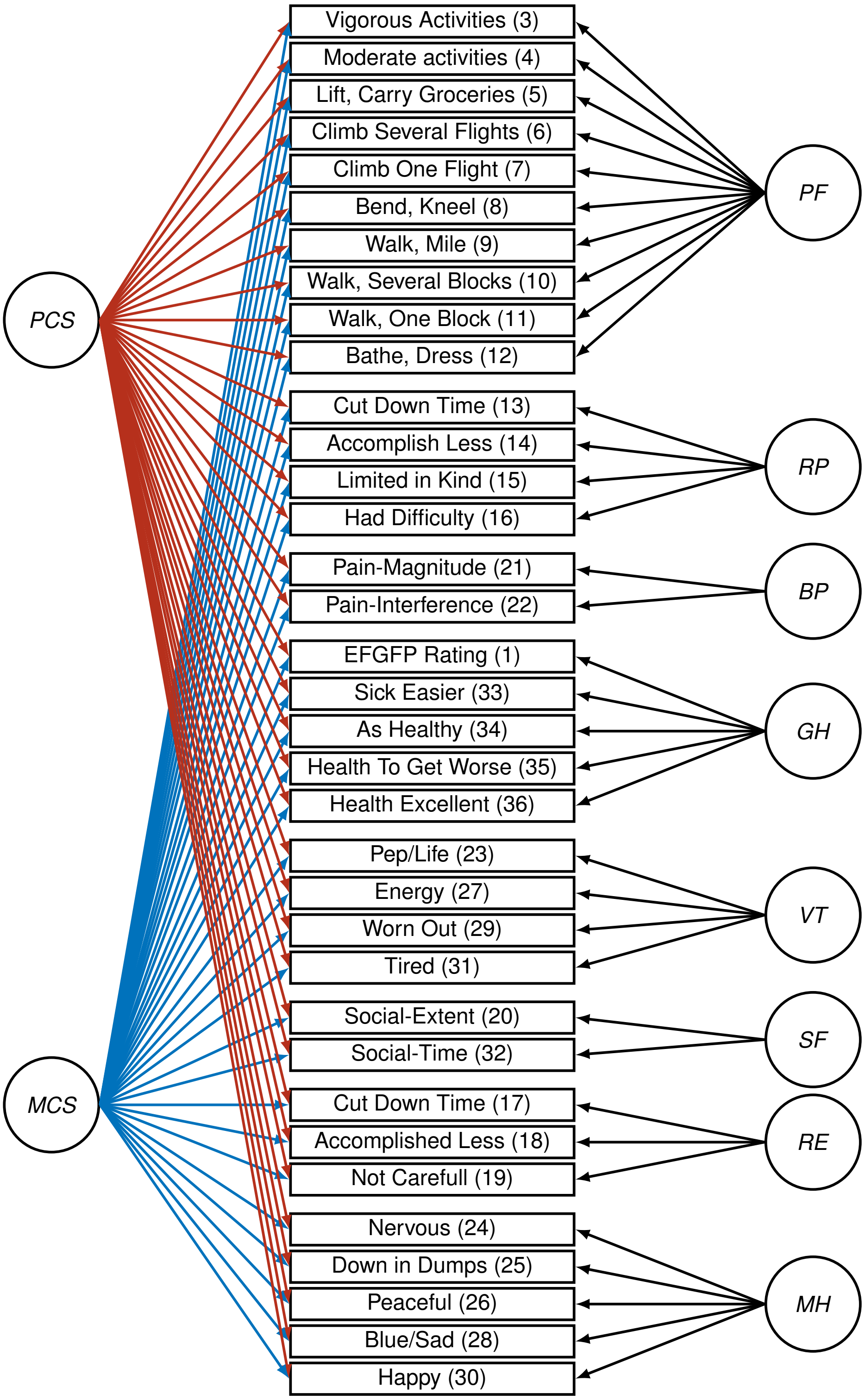

Figure 1.2: Path diagram of the final model. Factors, items and causal pathways are represented by circles, rectangles and arrows, respectively. Item number is presented within the parentheses.

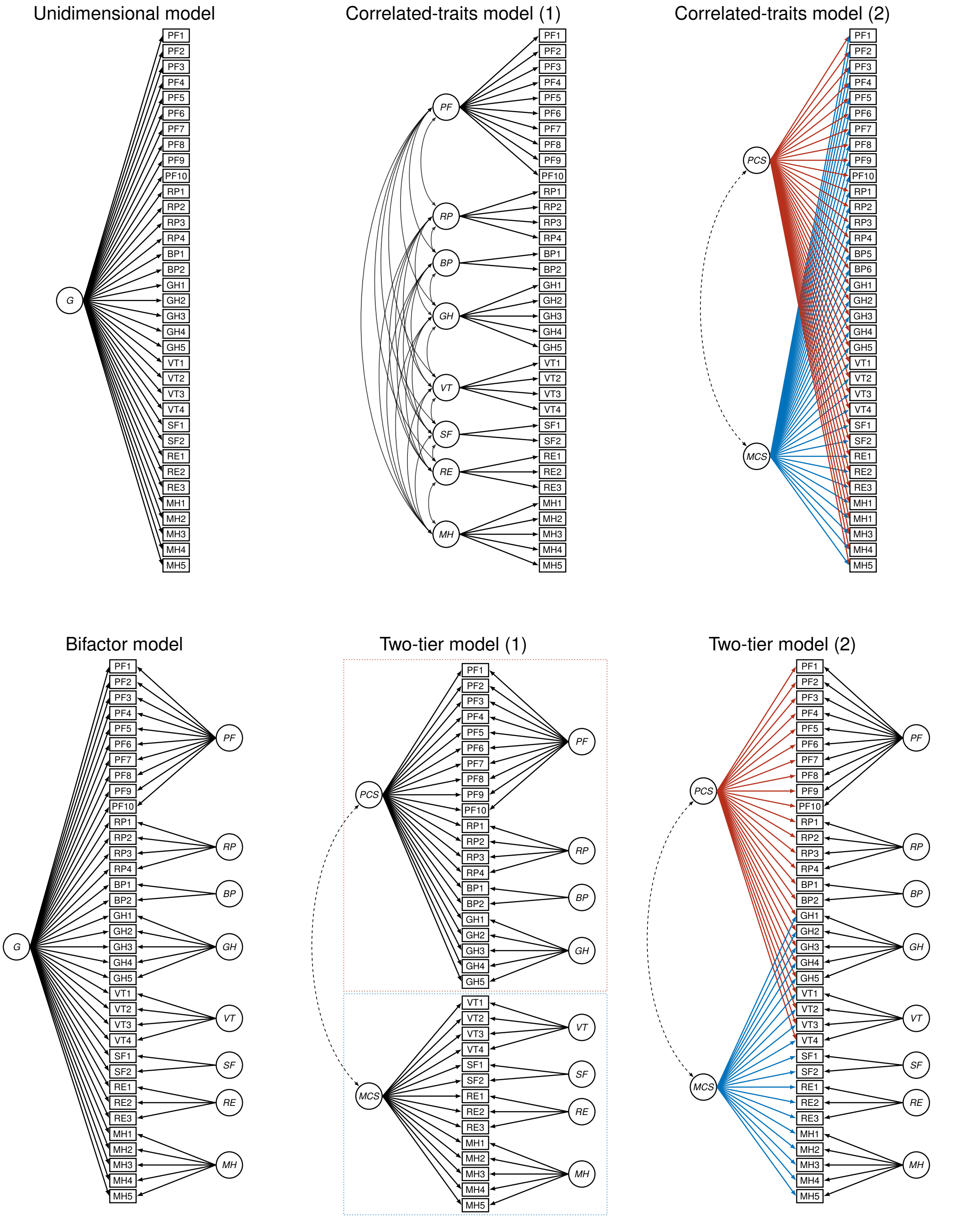

Figure 1.3: Path diagrams of the computed models. Factors, items and causal pathways are represented by circles, rectangles and arrows, respectively. The dotted covariance lines indicate that both correlated and orthogonal models were fitted. The dotted rectangles of the two-tier model (1) denote items included separately in unidimensional, correlated-traits and bifactor models of the physical (red) and mental (blue) factors.

1.4 Model selection

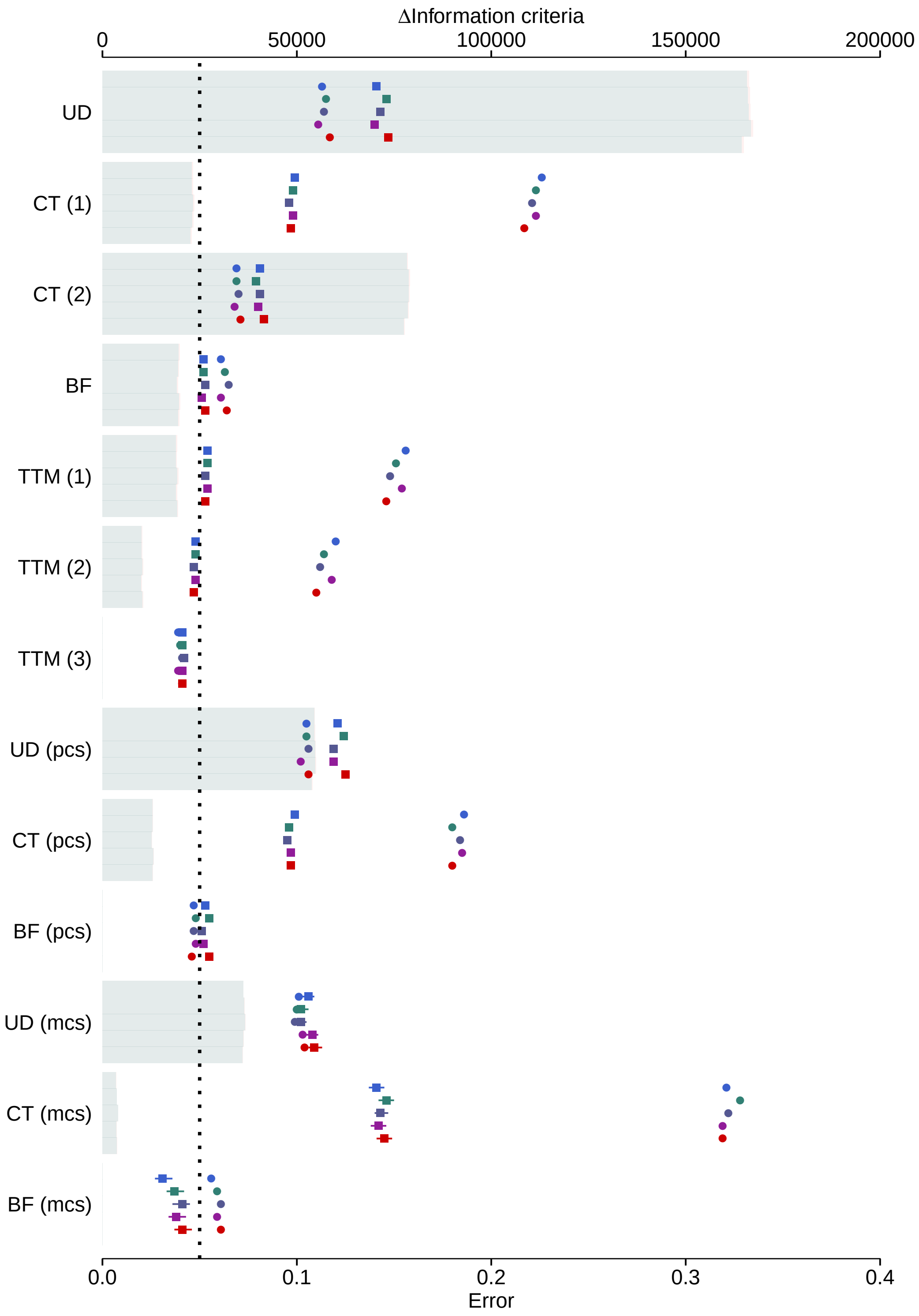

Figure 1.4: Global fit of the competing models. Squares (lines) and circles mark the point estimate (95% CI) of RMSEA and SRMSR of the validation models (fold *k*), respectively. Green and red bars denote the difference in AIC and BIC, relative to the best fitting model of the complete scale, physical part and mental part, respectively. Only orthonal models showed for CT(2) and TTM(1-3) as fit was nearly identical for oblique models. UD, unidimensional model. CT, correlated-traits model. BF, bifactor model. TTM, two-tier model with orthogonal general factors.

1.5 Final model

1.5.1 Theta distribution proxies

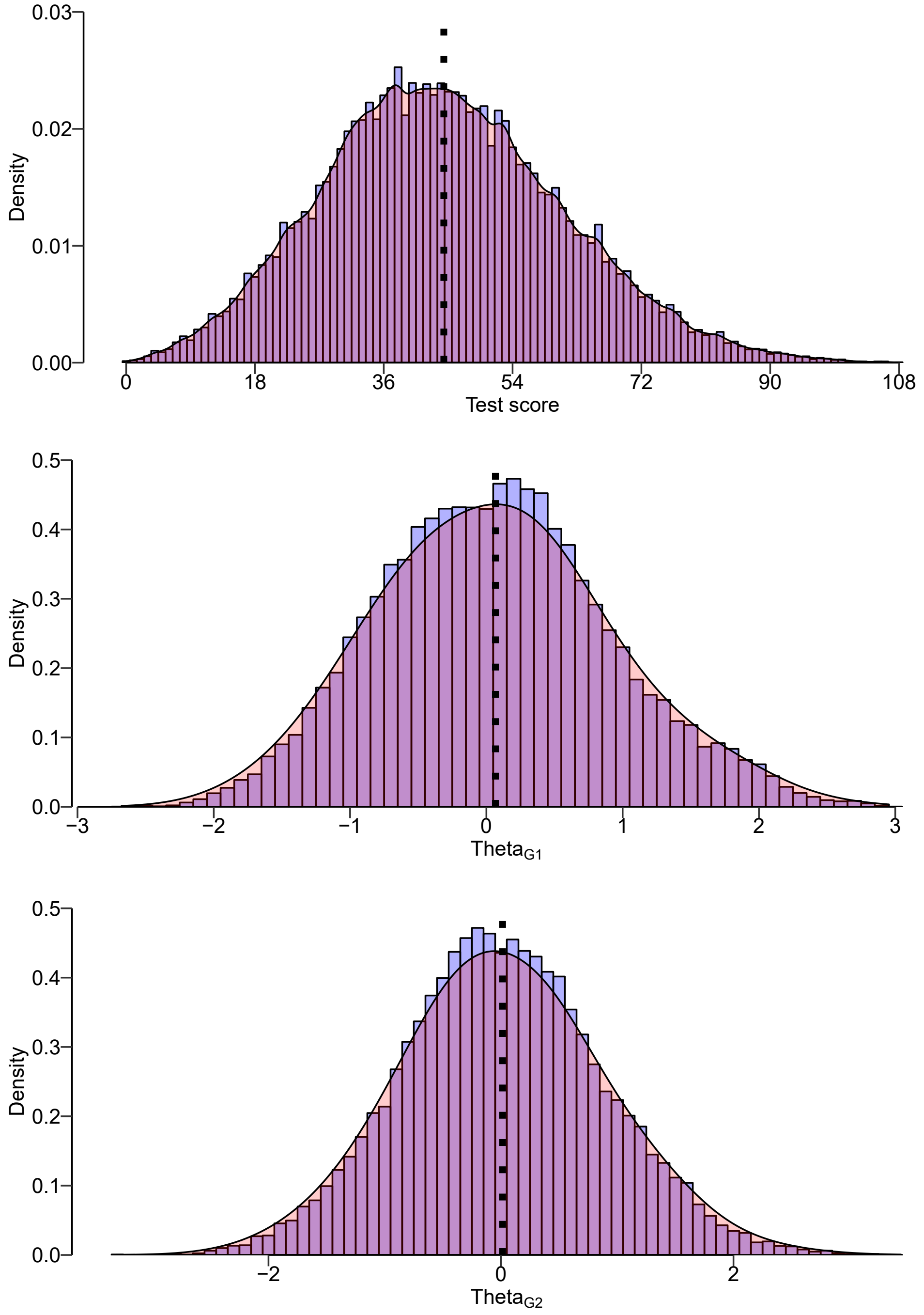

Figure 1.5: Histogram with overlayed density of test score (top) and estimated theta for the physical (middle) and mental (bottom) factors. The dotted vertical line marks the mean.

1.5.2 Item residual correlations

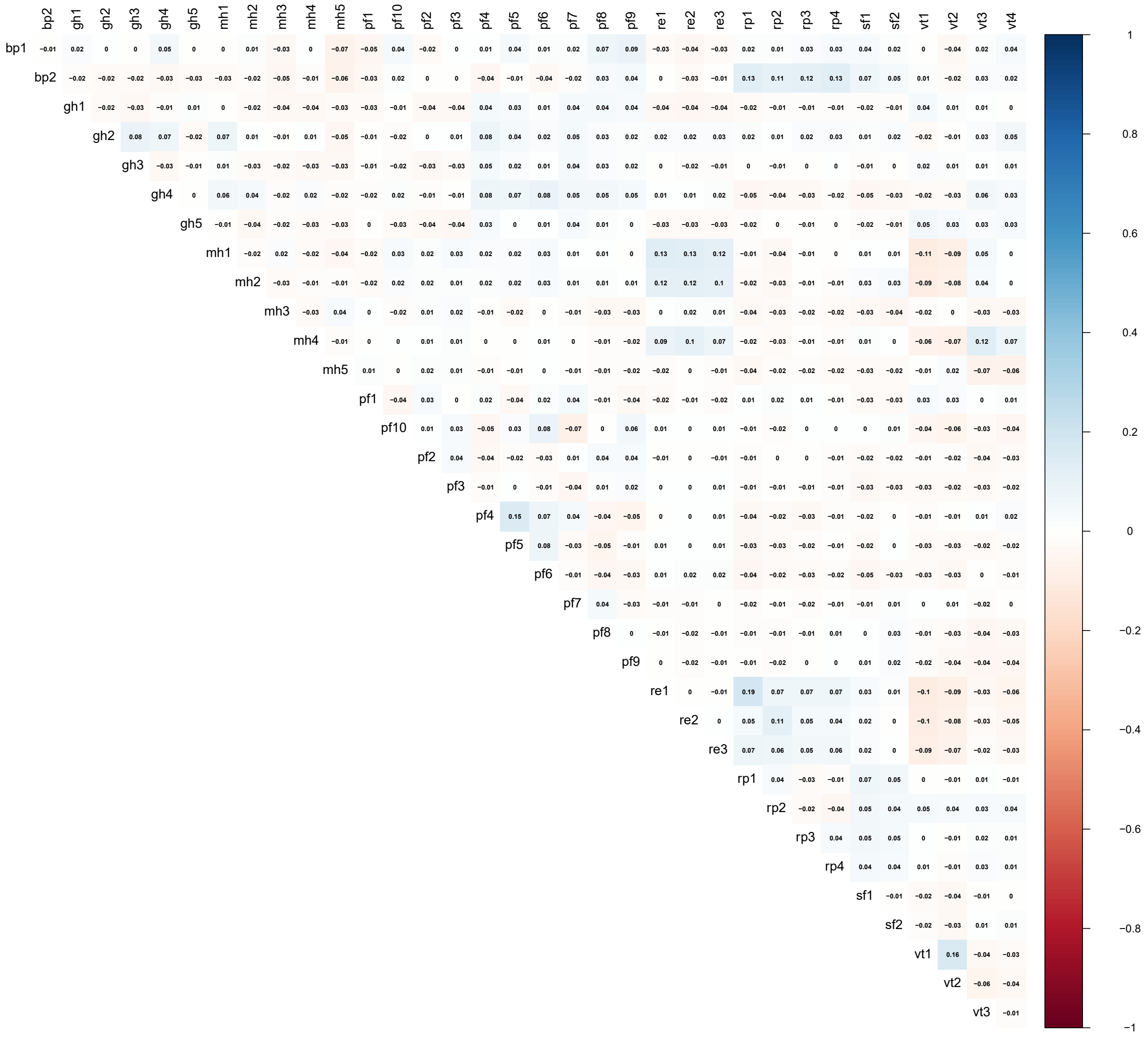

Figure 1.6: M_2^*-based item residuals.

1.5.3 Observed and expected item response proportions

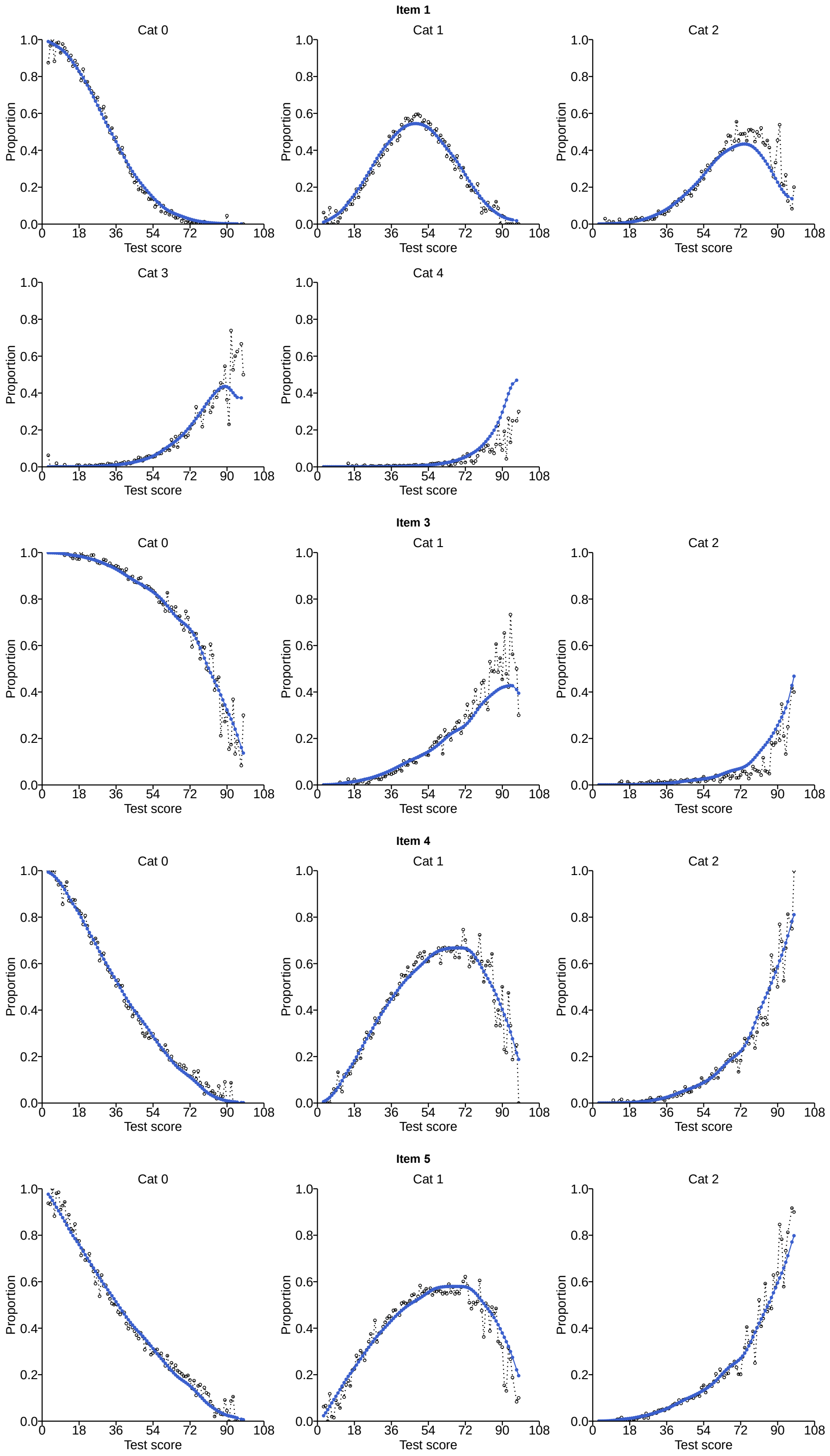

Figure 1.7: Observed and expected S-X^2-based proportions per category for items 1-5.

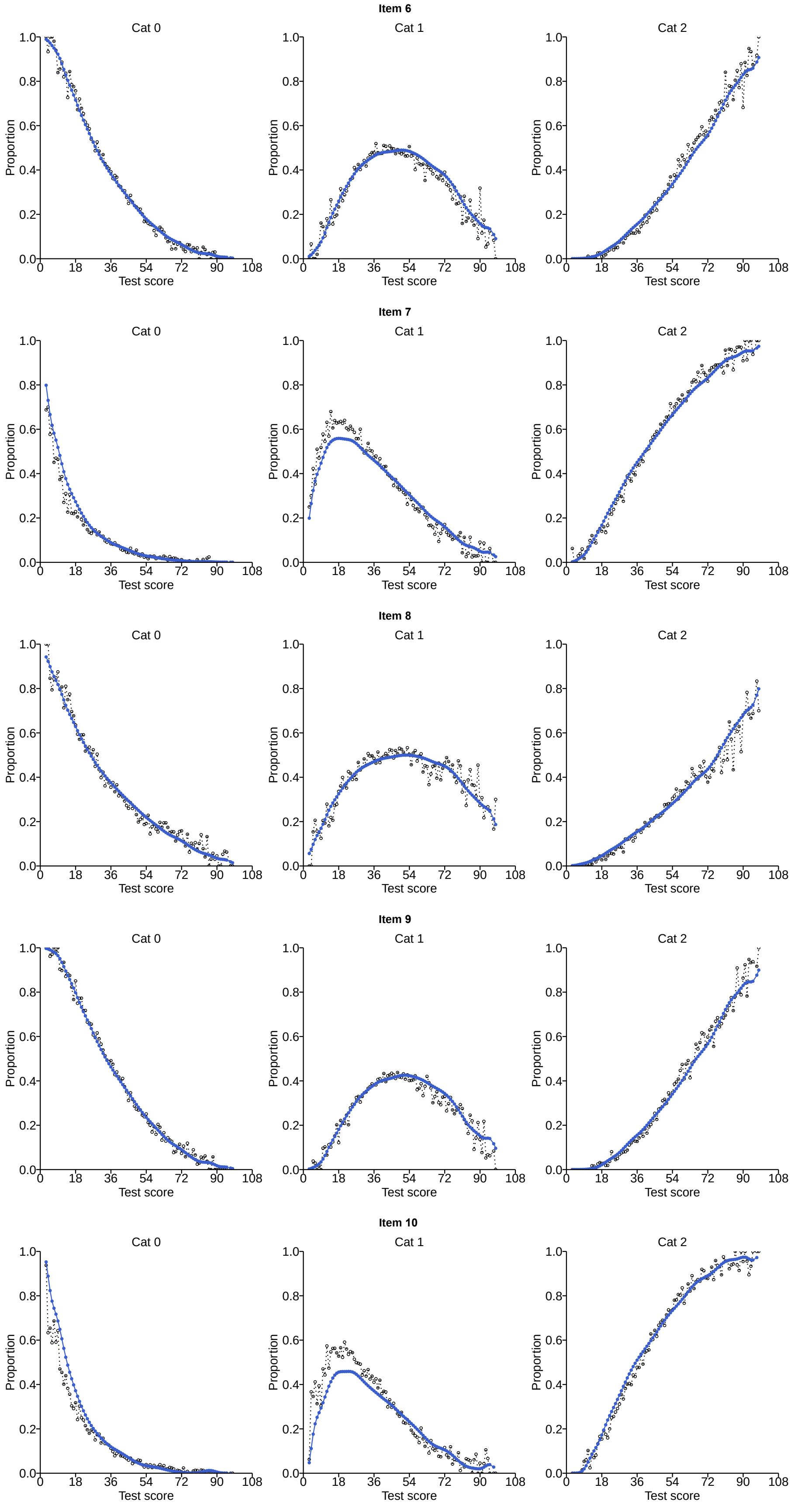

Figure 1.7: Observed and expected S-X^2-based proportions per category for items 6-10.

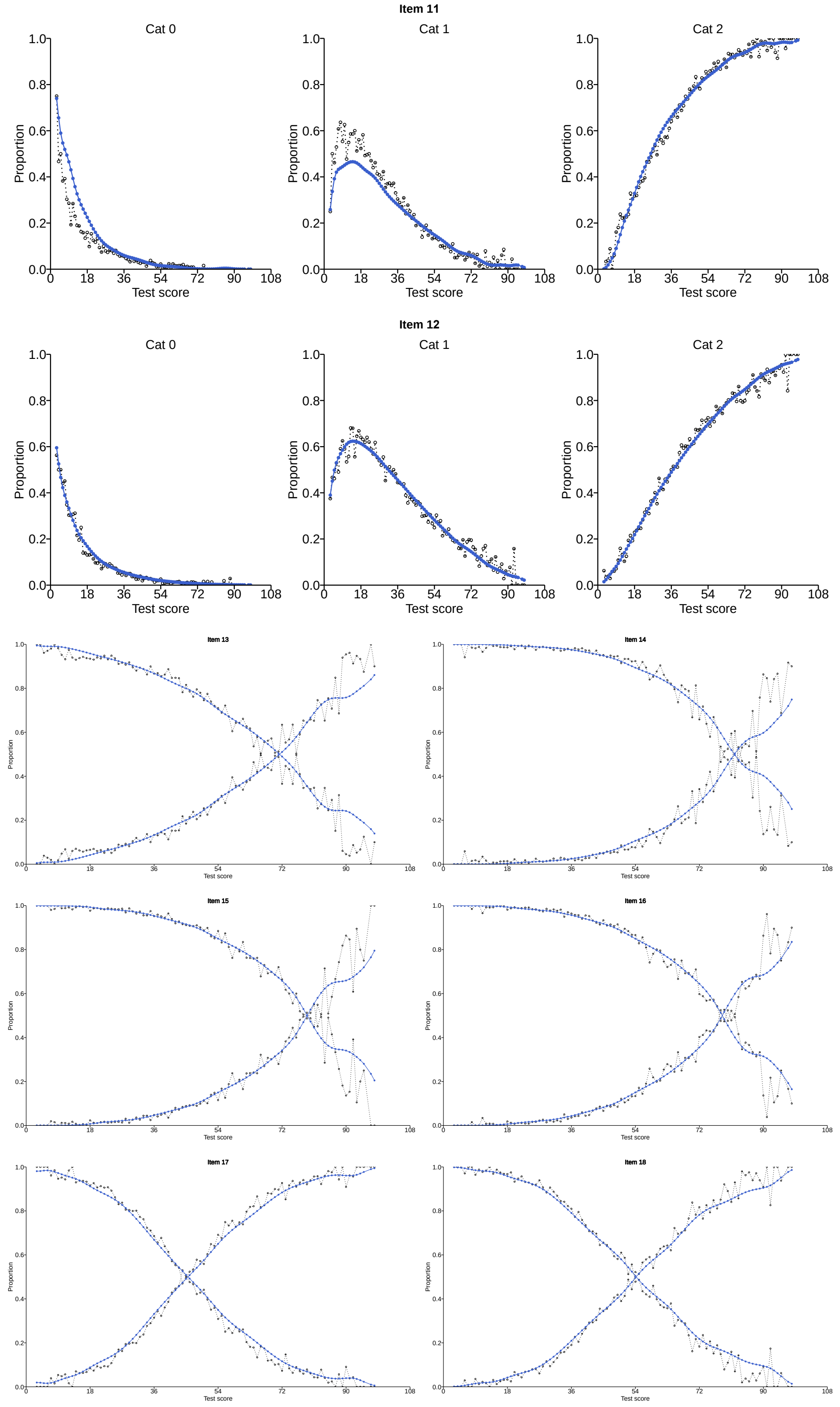

Figure 1.7: Observed and expected S-X^2-based proportions per category for items 11-18.

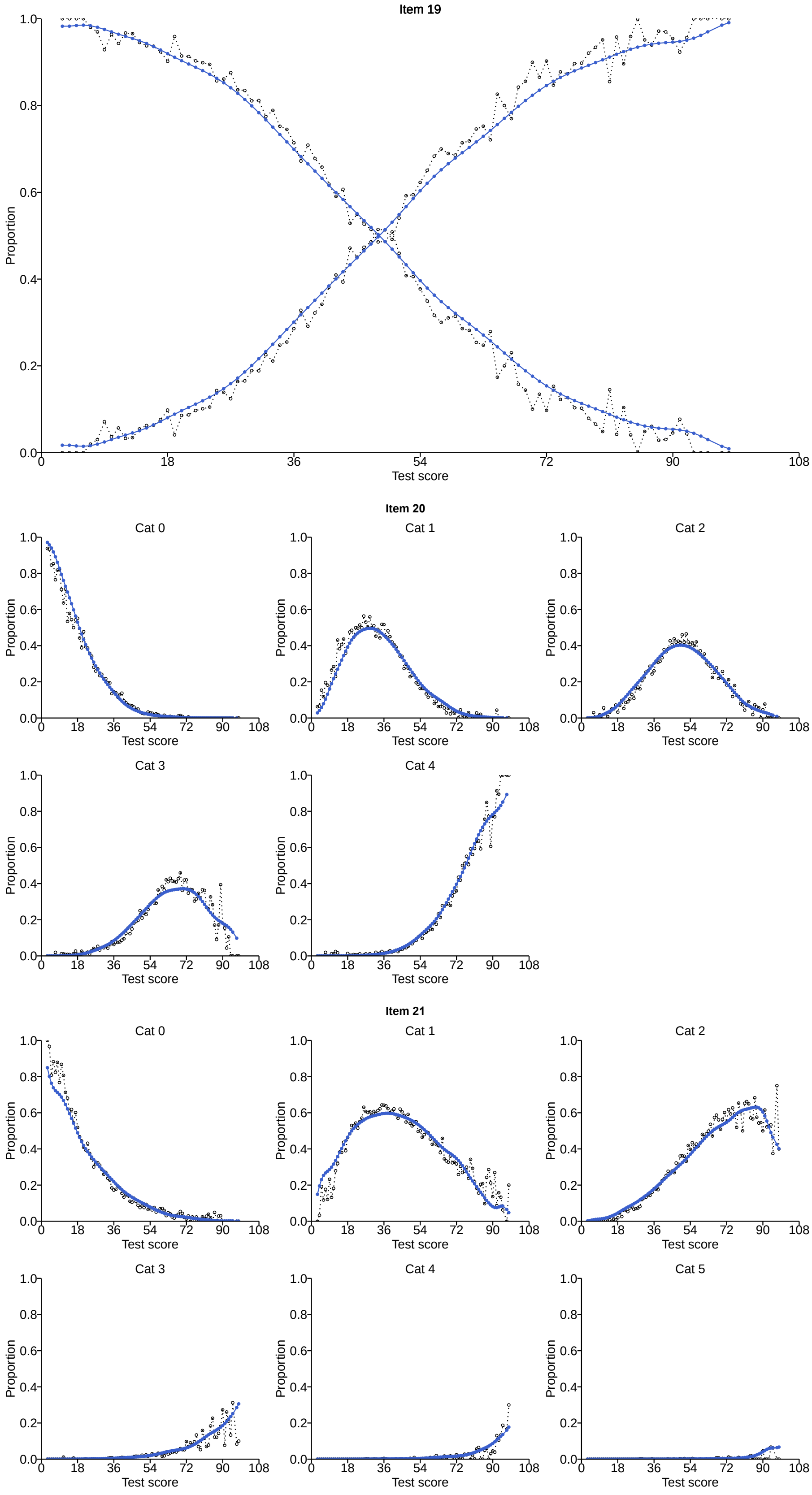

Figure 1.7: Observed and expected S-X^2-based proportions per category for items 19-21.

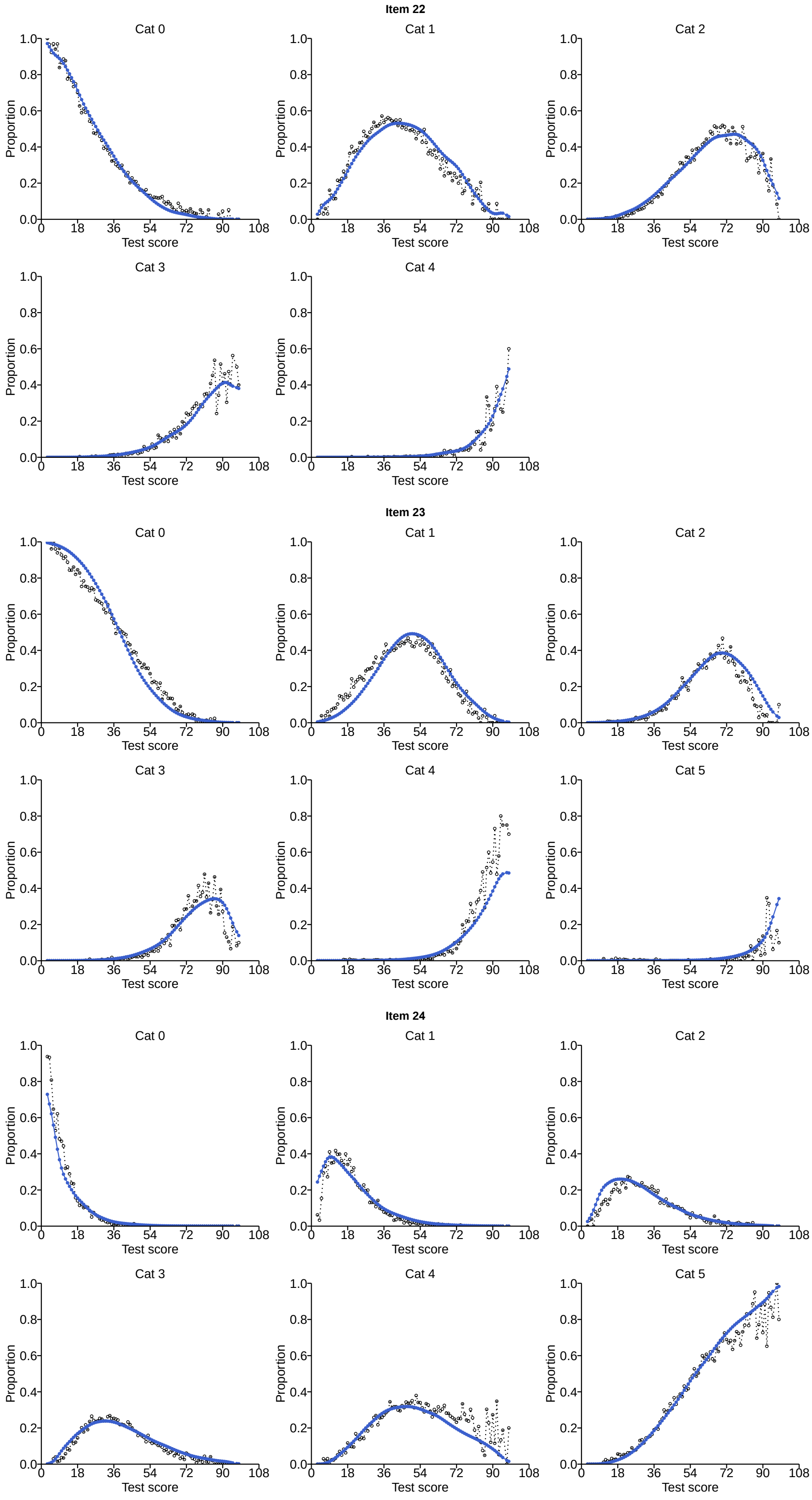

Figure 1.7: Observed and expected S-X^2-based proportions per category for items 22-24.

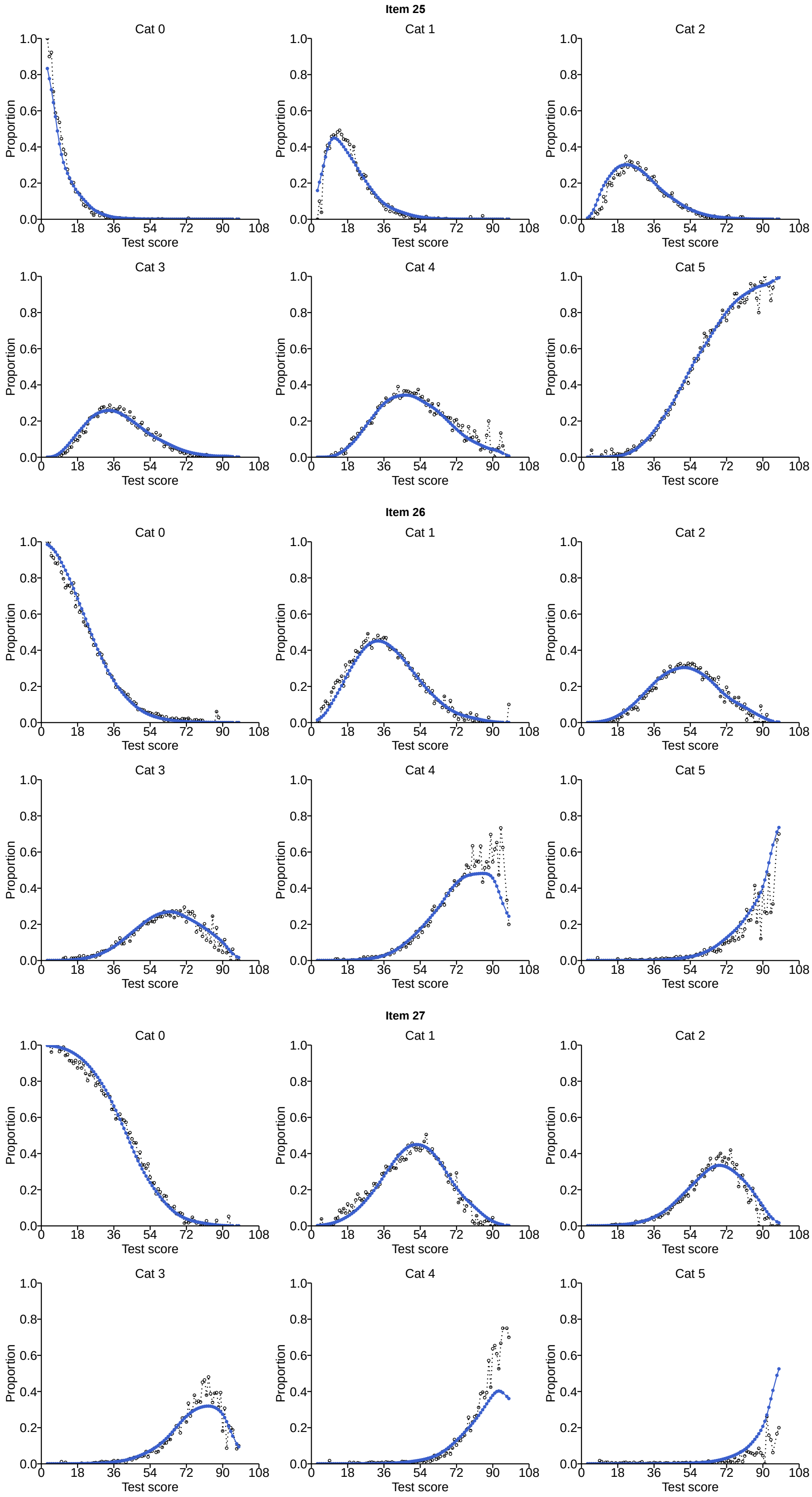

Figure 1.7: Observed and expected S-X^2-based proportions per category for items 25-27.

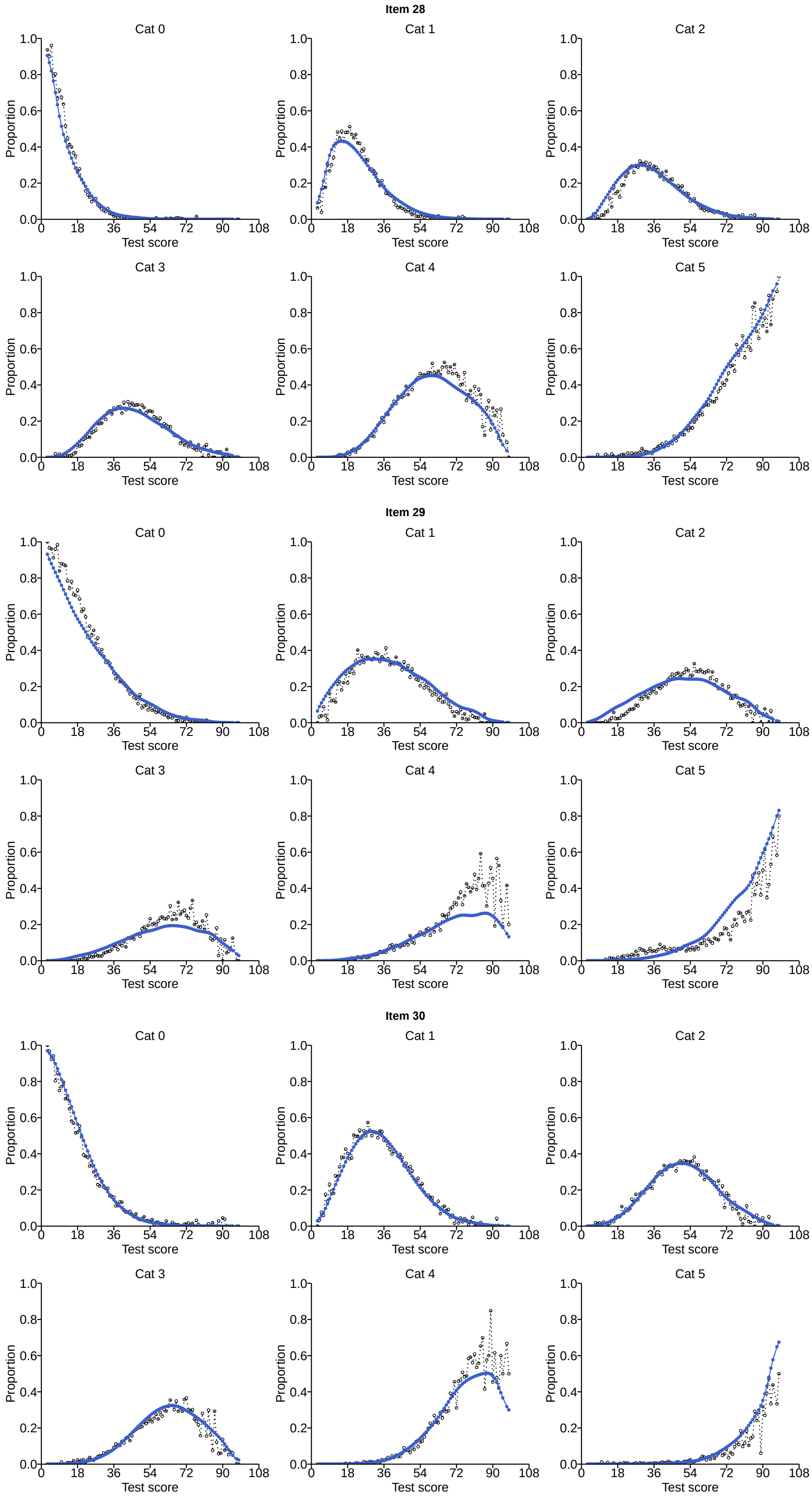

Figure 1.7: Observed and expected S-X^2-based proportions per category for items 28-30.

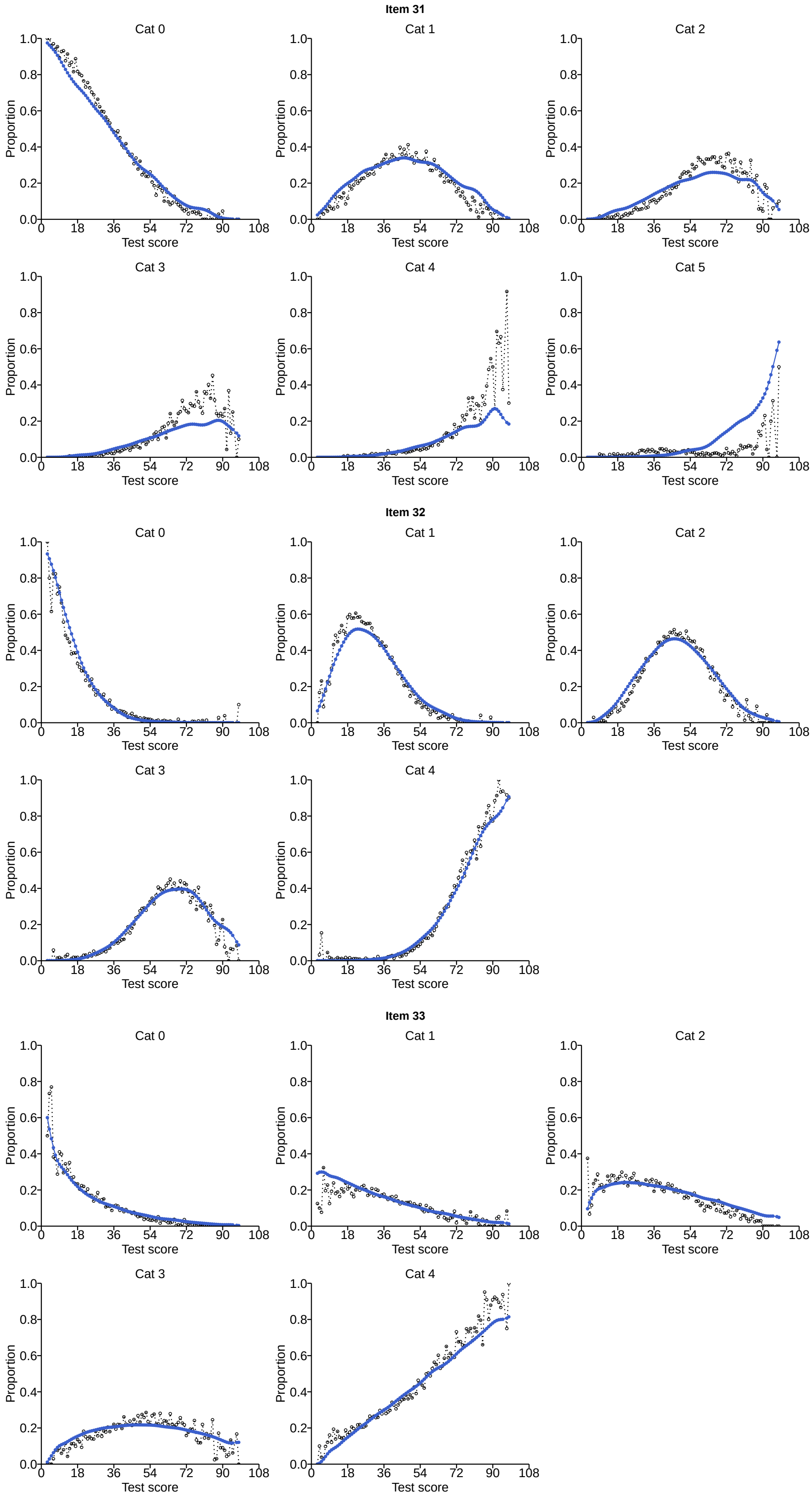

Figure 1.7: Observed and expected S-X^2-based proportions per category for items 31-33.

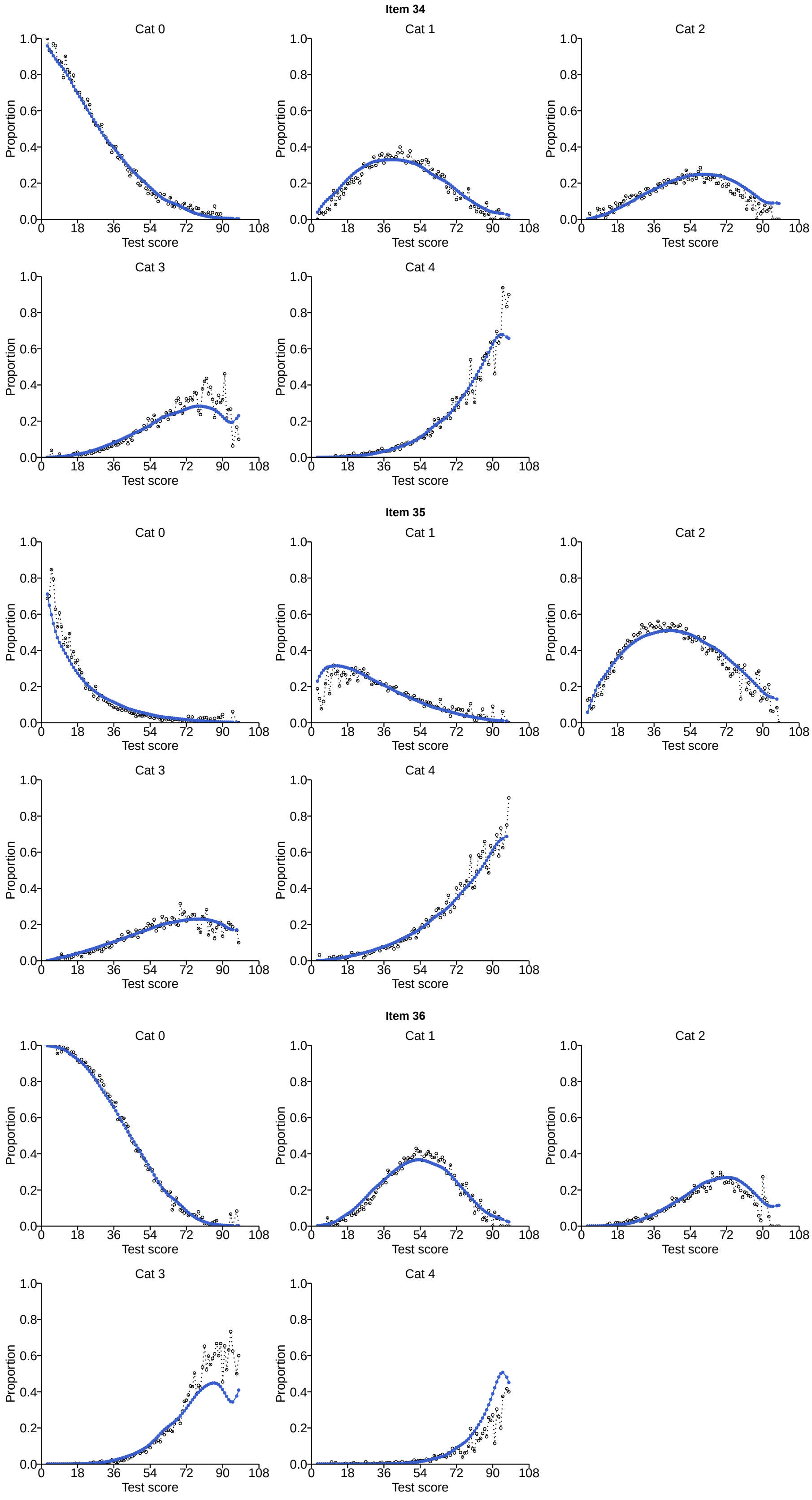

Figure 1.7: Observed and expected S-X^2-based proportions per category for items 34-36.

1.5.4 Marginal item category proportions

Table 1.2: Marginal item category response proportions based on S-X^2 frequencies.

	Observed (expected) proportions					
	**Cat 0**	**Cat 1**	**Cat 2**	**Cat 3**	**Cat 4**	**Cat 5**
Item 1	0.317 (0.322)	0.240 (0.245)	0.199 (0.177)	0.167 (0.145)	0.077 (0.112)	
Item 2	0.704 (0.706)	0.194 (0.173)	0.102 (0.121)			
Item 3	0.376 (0.373)	0.394 (0.392)	0.230 (0.235)			
Item 4	0.377 (0.372)	0.358 (0.371)	0.264 (0.257)			
Item 5	0.308 (0.307)	0.281 (0.293)	0.411 (0.400)			
Item 6	0.113 (0.127)	0.287 (0.278)	0.600 (0.595)			
Item 7	0.308 (0.305)	0.350 (0.350)	0.342 (0.345)			
Item 8	0.351 (0.350)	0.241 (0.249)	0.409 (0.401)			
Item 9	0.141 (0.164)	0.234 (0.206)	0.625 (0.630)			
Item 10	0.081 (0.107)	0.209 (0.184)	0.710 (0.709)			
Item 11	0.081 (0.086)	0.298 (0.293)	0.621 (0.620)			
Item 12	0.607 (0.626)	0.393 (0.374)				
Item 13	0.725 (0.750)	0.275 (0.250)				
Item 14	0.700 (0.716)	0.300 (0.284)				
Item 15	0.695 (0.709)	0.305 (0.291)				
Item 16	0.427 (0.431)	0.573 (0.569)				
Item 17	0.504 (0.512)	0.496 (0.488)				
Item 18	0.449 (0.454)	0.551 (0.546)				
Item 19	0.186 (0.194)	0.192 (0.184)	0.165 (0.166)	0.153 (0.156)	0.304 (0.300)	
Item 20	0.214 (0.203)	0.354 (0.351)	0.325 (0.301)	0.055 (0.071)	0.031 (0.045)	0.021 (0.030)
Item 21	0.283 (0.279)	0.264 (0.270)	0.207 (0.212)	0.127 (0.127)	0.120 (0.112)	
Item 22	0.364 (0.366)	0.198 (0.198)	0.123 (0.141)	0.098 (0.105)	0.139 (0.113)	0.077 (0.077)
Item 23	0.097 (0.085)	0.087 (0.095)	0.083 (0.092)	0.095 (0.102)	0.195 (0.161)	0.442 (0.464)
Item 24	0.098 (0.089)	0.095 (0.099)	0.085 (0.093)	0.088 (0.093)	0.154 (0.145)	0.480 (0.481)
Item 25	0.238 (0.242)	0.179 (0.169)	0.120 (0.124)	0.106 (0.109)	0.199 (0.185)	0.157 (0.171)
Item 26	0.395 (0.396)	0.169 (0.172)	0.114 (0.118)	0.105 (0.101)	0.131 (0.103)	0.085 (0.109)
Item 27	0.130 (0.120)	0.116 (0.119)	0.098 (0.106)	0.109 (0.111)	0.221 (0.207)	0.326 (0.337)
Item 28	0.257 (0.240)	0.157 (0.177)	0.119 (0.127)	0.103 (0.095)	0.161 (0.117)	0.204 (0.244)
Item 29	0.197 (0.202)	0.199 (0.192)	0.139 (0.140)	0.125 (0.130)	0.208 (0.184)	0.132 (0.152)
Item 30	0.346 (0.339)	0.169 (0.190)	0.140 (0.136)	0.109 (0.088)	0.154 (0.082)	0.081 (0.164)
Item 31	0.148 (0.155)	0.191 (0.182)	0.186 (0.194)	0.166 (0.170)	0.309 (0.299)	
Item 32	0.117 (0.114)	0.102 (0.120)	0.144 (0.150)	0.147 (0.160)	0.489 (0.457)	
Item 33	0.304 (0.299)	0.172 (0.180)	0.121 (0.138)	0.148 (0.147)	0.254 (0.237)	
Item 34	0.144 (0.130)	0.129 (0.136)	0.319 (0.326)	0.120 (0.135)	0.287 (0.274)	
Item 35	0.417 (0.413)	0.165 (0.168)	0.104 (0.119)	0.196 (0.186)	0.118 (0.115)	

1.5.5 Person fit

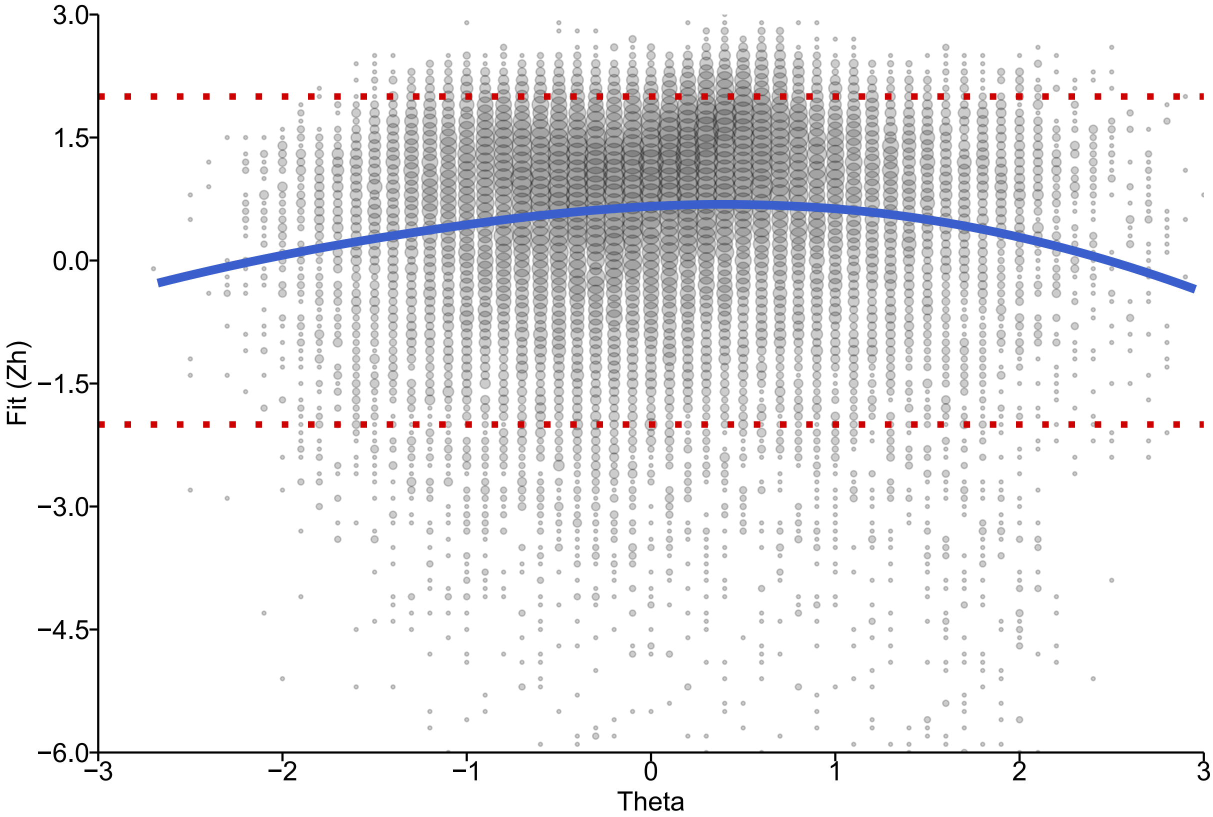

Figure 1.8: Person fit based on Zh. Bubbles depict estimates with increased size and darkness showing higher density. The trend is represented by the fitted line from a generalized additive model with smoothed integration. The dotted red lines mark ±2.

1.6 Parameter estimate robustness

1.6.1 Item parameters

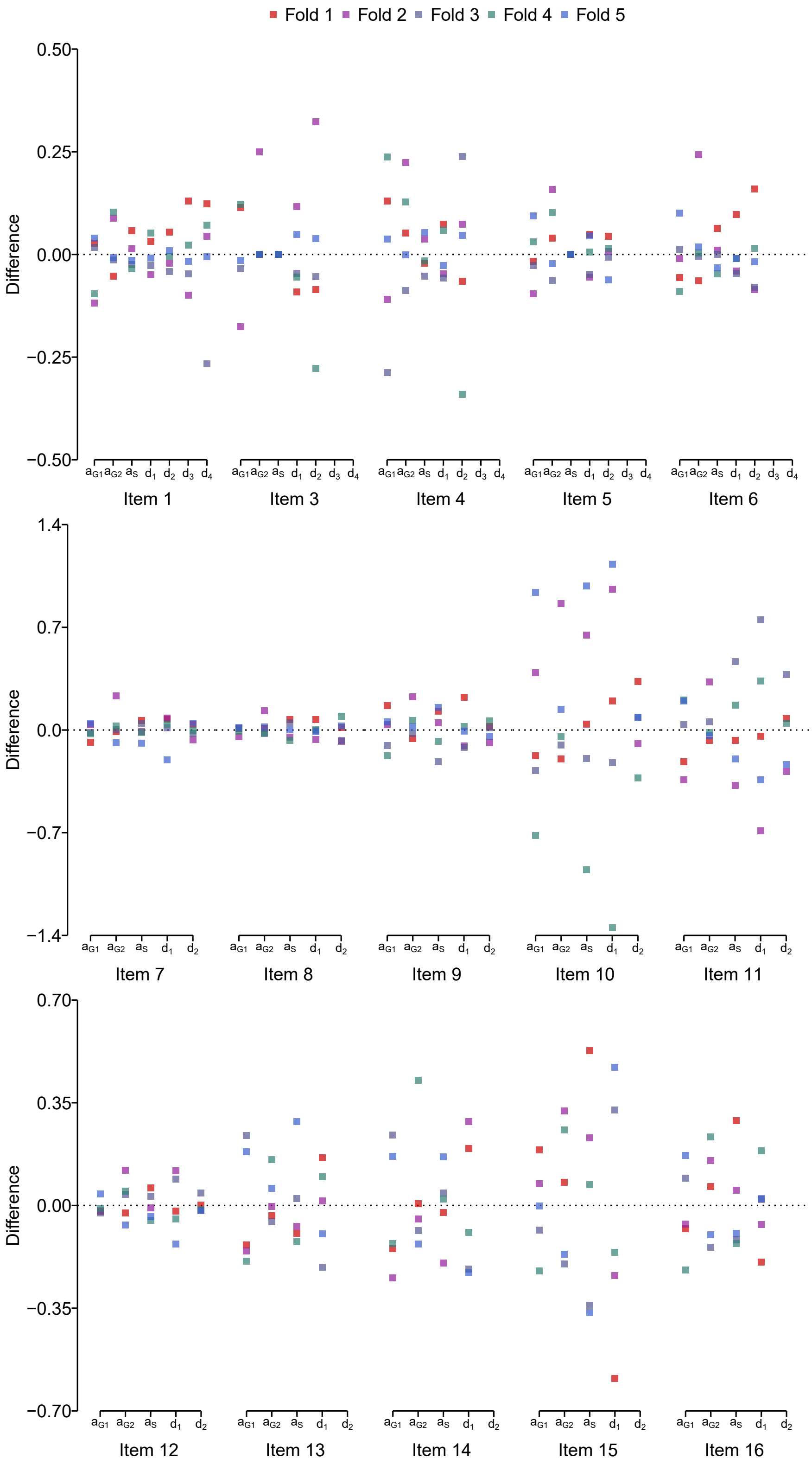

Figure 1.9: Parameter estimate difference between training (excluding fold *k*) and validation (fold *k*) models for items 1-16.

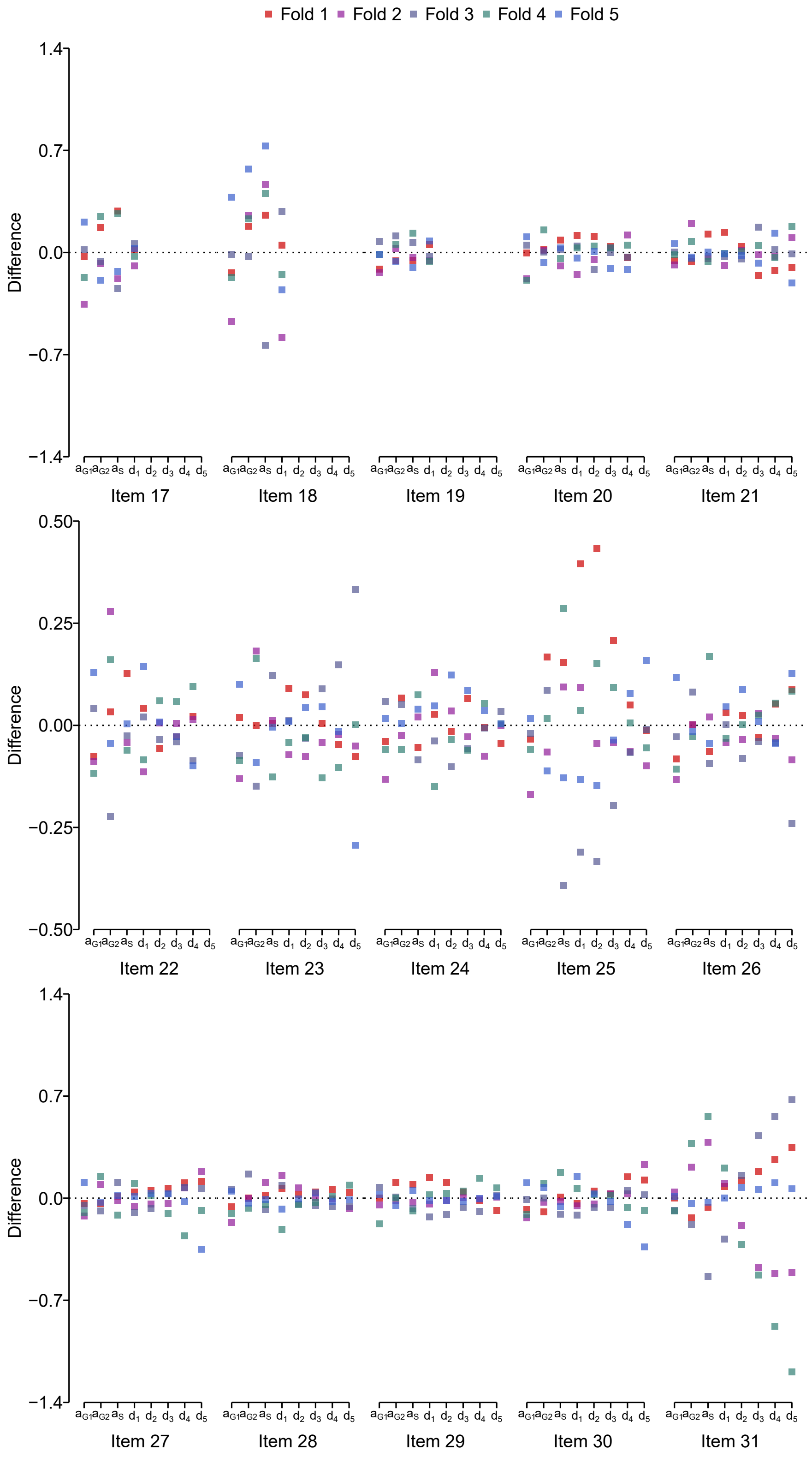

Figure 1.9: Parameter estimate difference between training (excluding fold k) and validation (fold k) models for items 17-31.

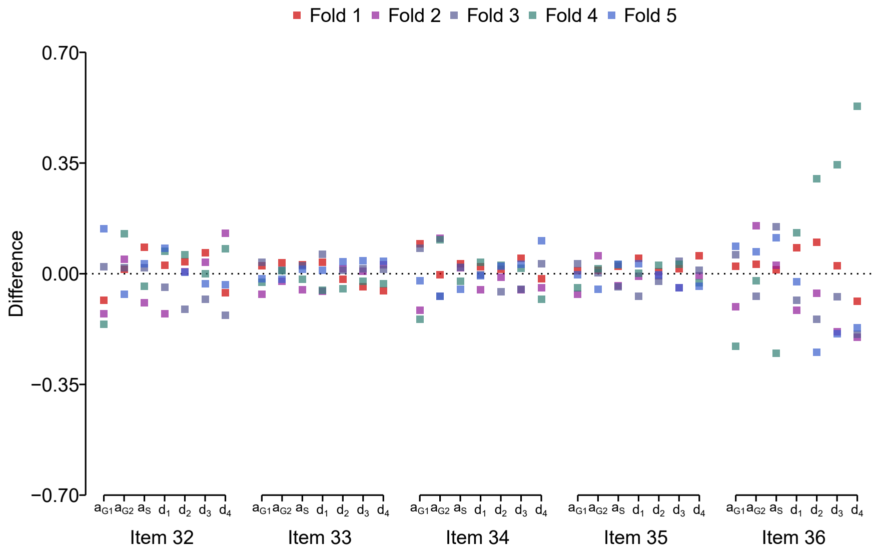

Figure 1.9: Parameter estimate difference between training (excluding fold *k*) and validation (fold *k*) models for items 32-36.

1.6.2 Person estimates

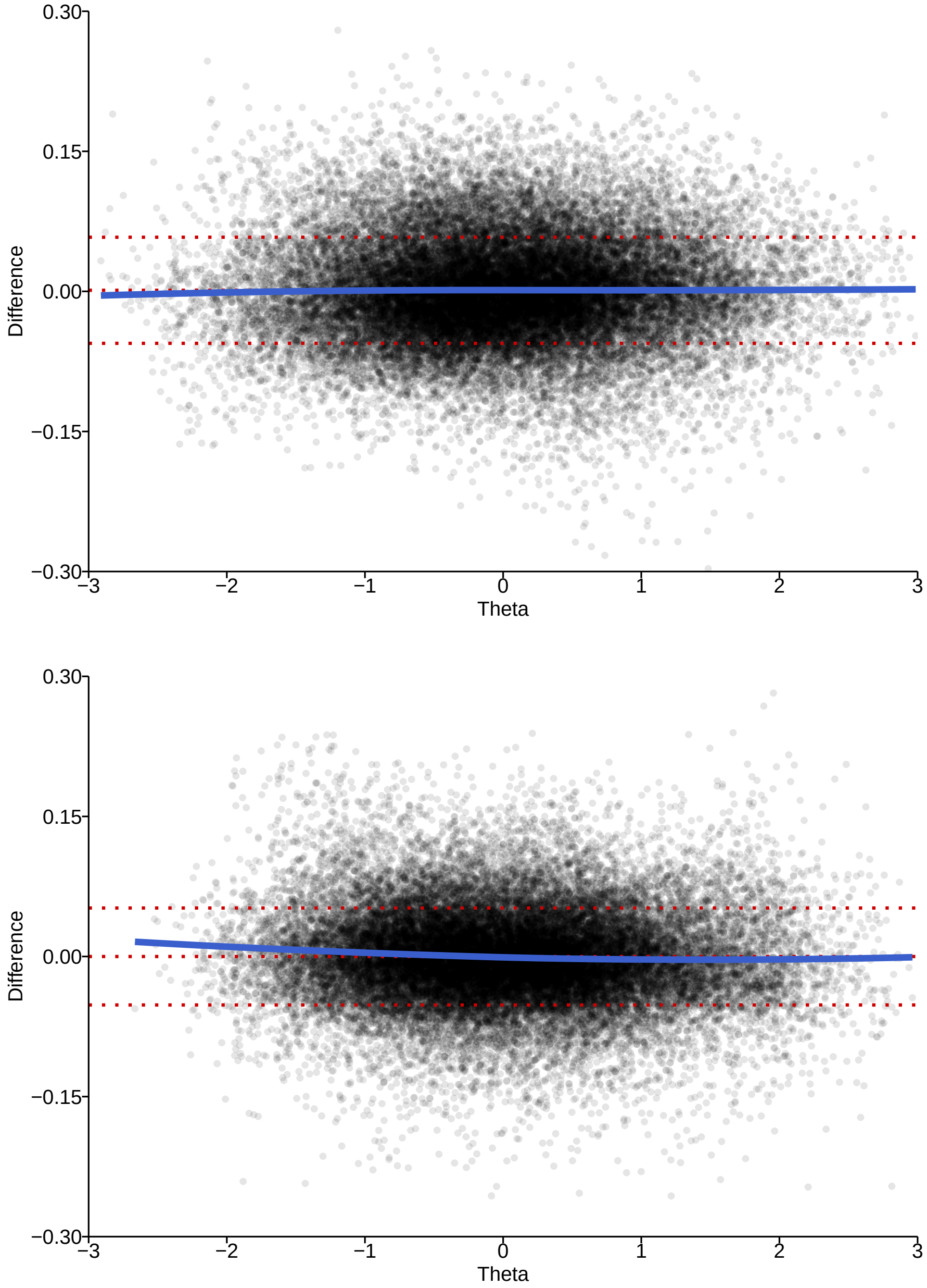

Figure 1.10: Difference in theta estimates of the physical (top) and mental (bottom) factors between the training (excluding fold k) and validation (fold k) models. The sample trend is depicted by a fitted generalized additive model line with smoothed integration (blue line). Points show individual observations, with darker areas indicating higher density. Red dotted lines mark mean and one standard deviation.

2. EQ-5D

2.1 Questionnaire

Table 2.1: English version of EQ-5D-3L

Item	Reponse option (score)
(1) Mobility	I have no problems walking about (0)<br>I have some problems walking about (1)<br>I am confined to bed (2)
(2) Self-Care	I have no problems with self-care (0)<br>I have some problems washing or dressing myself (1)<br>I am unable to wash or dress myself (2)
(3) Usual activities *(e.g., work, study, housework, family or leisure activities)*	I have no problems with performing my usual activities (0)<br>I have some problems with performing my usual activities (1)<br>I am unable to perform my usual activities (2)
(4) Pain/Discomfort	I have no pain or discomfort (0)<br>I have moderate pain or discomfort (1)<br>I have extreme pain or discomfort (2)
(5) Anxiety/Depression	I am not anxious or depressed (0)<br>I am moderately anxious or depressed (1)<br>I am extremely anxious or depressed (2)

Adapted from *www.euroqol.org*.

2.2 Item response distribution

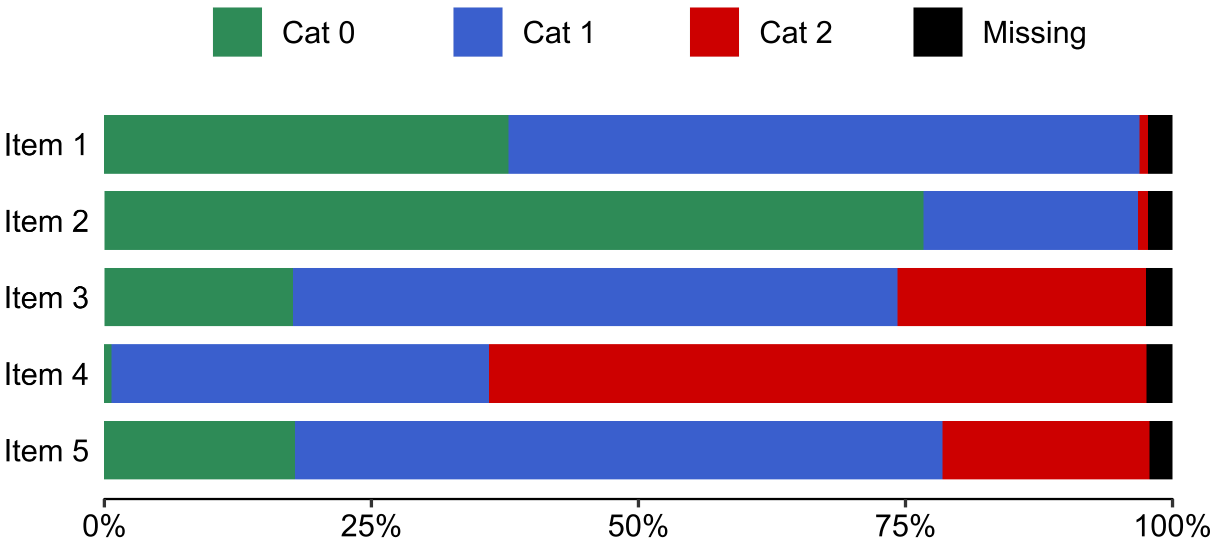

Figure 2.1: Relative item response frequencies (n = 35,908). Cat, category as defined in Table 2.1.

2.3 Model path diagram

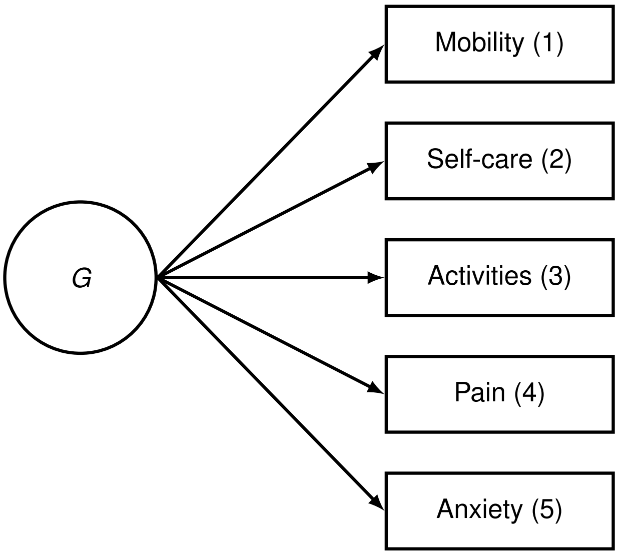

Figure 2.2: Path diagram of the final model. Factors, items and causal pathways are represented by circles, rectangles and arrows, respectively. Item number is presented within the parentheses.

2.4 Model selection

Table 2.2: Global unidimensional model fit per fold

Model	AIC, BIC	$C2_{(df)}$: p	RMSEA (90% CI)	SRMSR	TLI, CFI
Fold 1	206124, 206248	37(2): <0.001	0.051 (0.037, 0.065)	0.035	0.917, 0.967
Fold 2	206416, 206540	22(2): <0.001	0.038 (0.025, 0.053)	0.033	0.954, 0.982
Fold 3	206347, 206470	31(2): <0.001	0.046 (0.033, 0.061)	0.036	0.931, 0.973
Fold 4	206158, 206281	18(2): <0.001	0.034 (0.021, 0.049)	0.032	0.956, 0.982
Fold 5	205945, 206069	50(2): <0.001	0.059 (0.045, 0.074)	0.032	0.892, 0.957

AIC, Akaike's information criterion. BIC, Schwartz's bayesian information criterion. df, degrees of freedom. RMSEA, root mean square error of approximation. CI, confidence interval. SRMSR, standardized root mean square residual. TLI, Tucker Lewis index. CFI, comparative fit index.

2.5 Final model

2.5.1 Theta distribution proxies

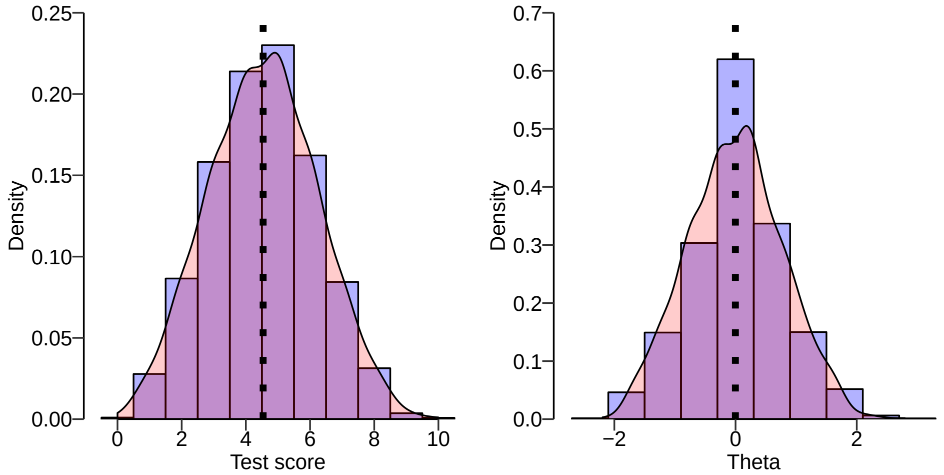

Figure 2.3: Histograms with overlayed density of test score (left) and estimated theta (right). Dotted vertical lines show the mean.

2.5.2 Item residual correlations

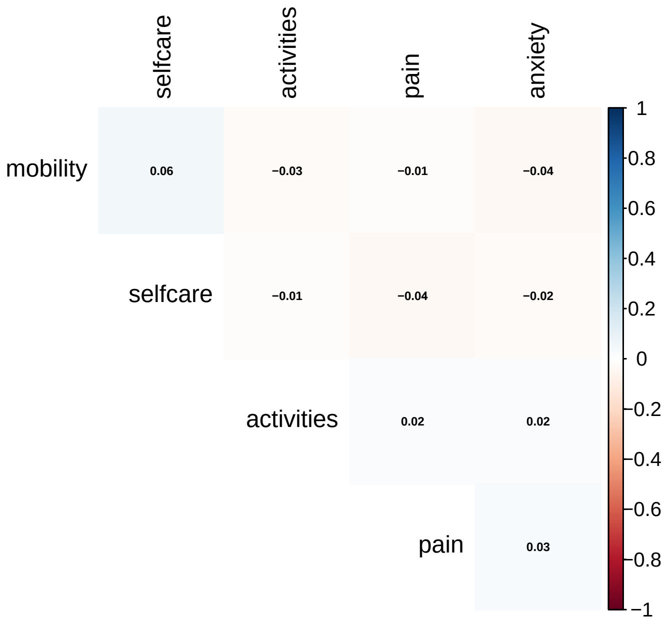

Figure 2.4: M_2^*-based item residuals.

2.5.3 Observed and expected item response proportions

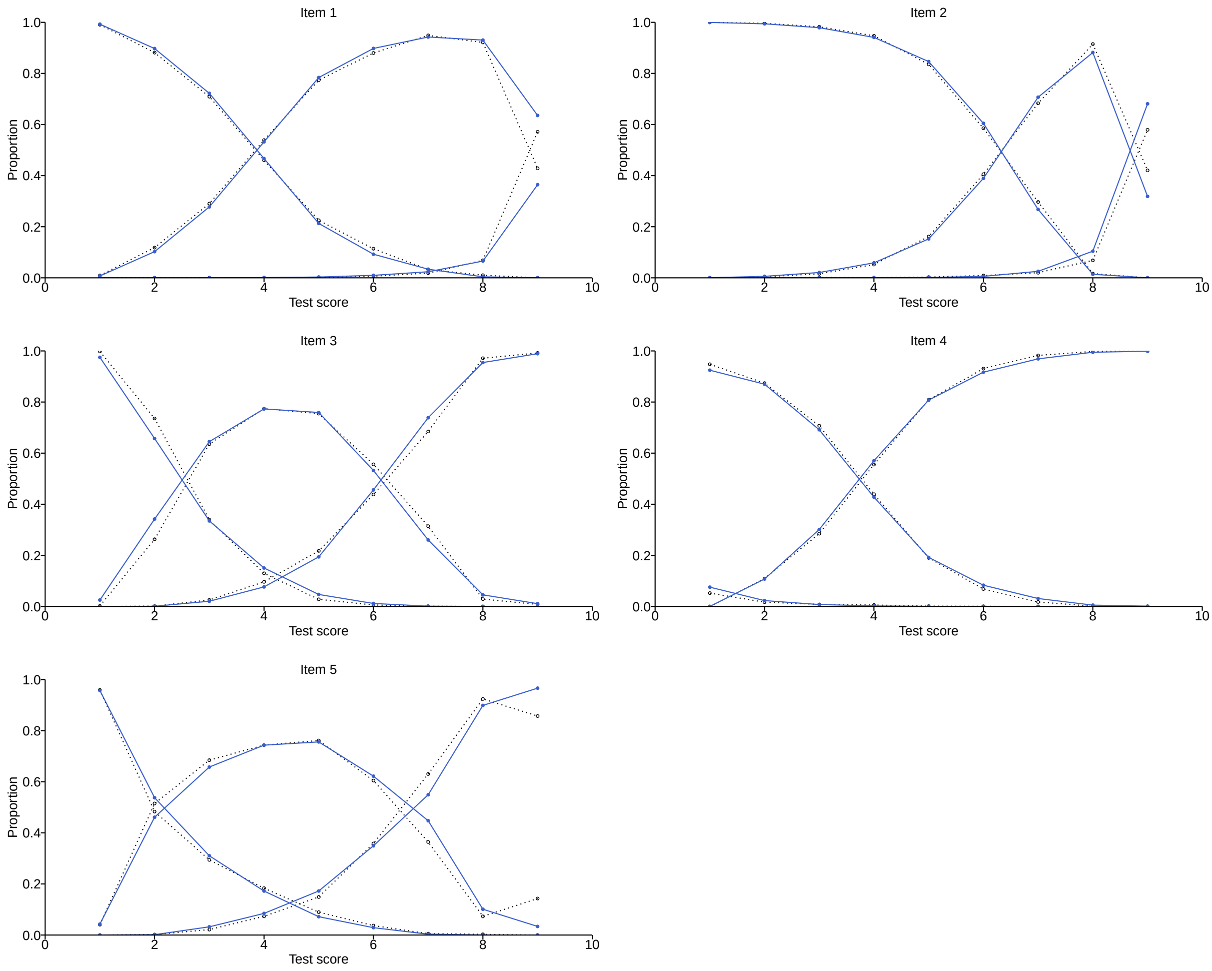

Figure 2.5: Observed and expected S-X^2-based proportions per item category.

2.5.4 Marginal item category proportions

Table 2.3: Marginal item category response proportions based on S-X^2 frequencies

	Observed (expected) proportions		
	**Cat 0**	**Cat 1**	**Cat 2**
Item 1	0.380 (0.380)	0.546 (0.568)	0.074 (0.052)
Item 2	0.629 (0.627)	0.295 (0.282)	0.076 (0.091)
Item 3	0.249 (0.242)	0.371 (0.377)	0.381 (0.381)
Item 4	0.009 (0.012)	0.361 (0.358)	0.630 (0.630)
Item 5	0.228 (0.231)	0.437 (0.429)	0.335 (0.339)

2.5.5 Person fit

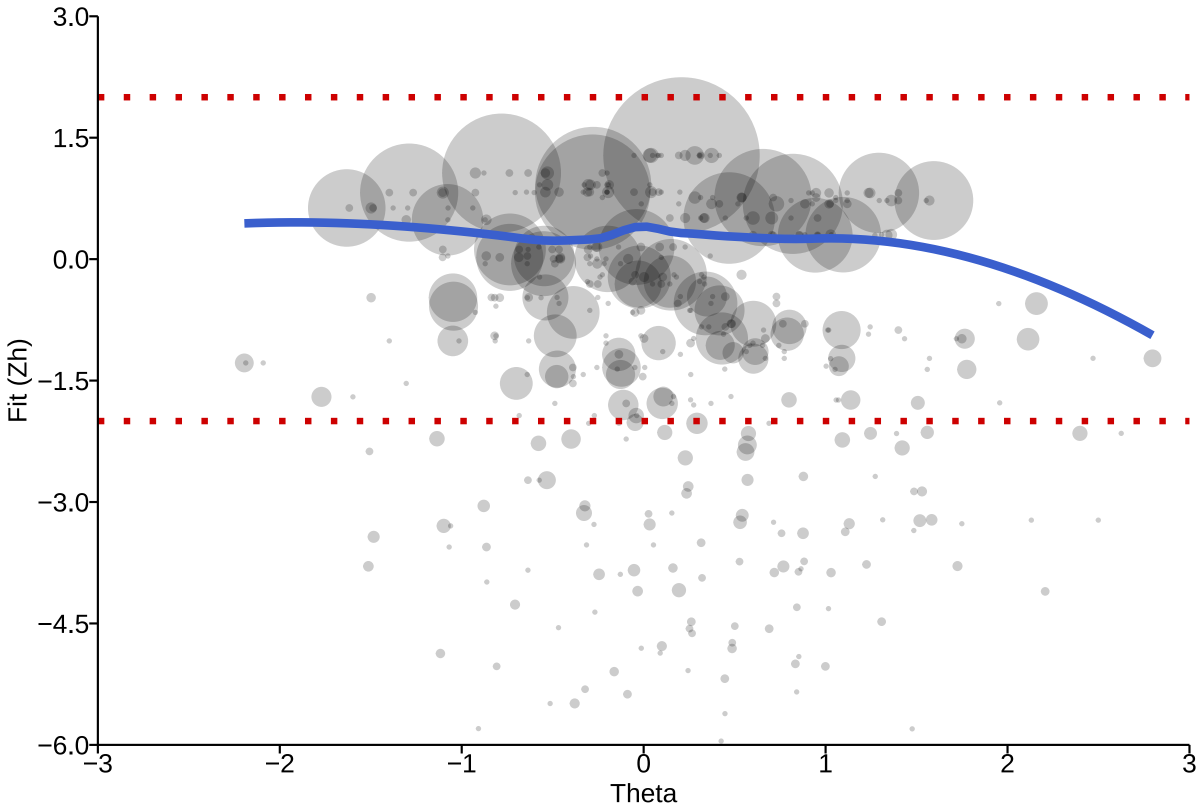

Figure 2.6: Person fit based on Zh. Bubbles depict estimates with increased size and darkness showing higher density. The trend is represented by the fitted line from a generalized additive model with smoothed integration. The dotted red lines mark ±2.

2.6 Parameter estimate robustness

2.6.1 Item parameters

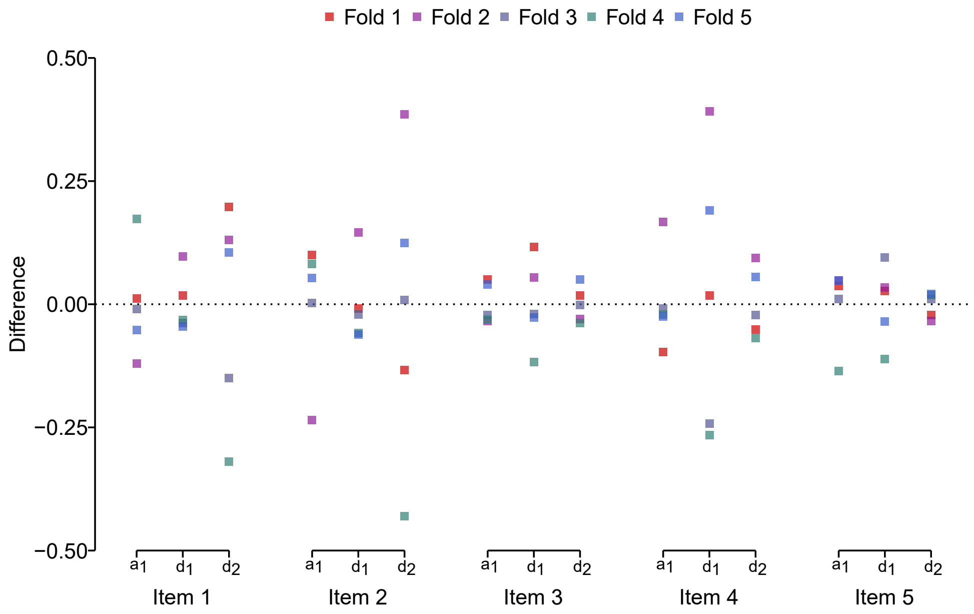

Figure 2.7: Parameter estimate difference between training (excluding fold k) and validation (fold k) models.

2.6.2 Person estimates

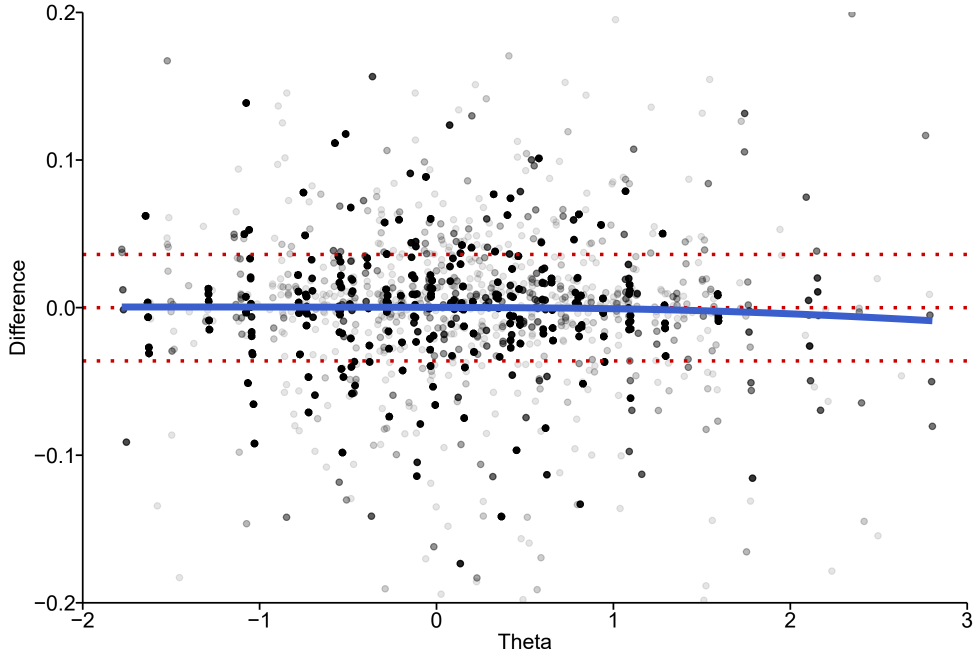

Figure 2.8: Difference in theta estimates between the training (excluding fold k) and validation (fold k) models. The sample trend is depicted by a fitted generalized additive model line with smoothed integration (blue line). Points show individual observations, with darker areas indicating higher density. Red dotted lines mark mean and one standard deviation.

3. Hospital Anxiety and Depression Scale

3.1 Questionnaire

Table 3.1: English version of the Hospital Anxiety and Depression scale

**Item**	**Reponse option (score)**
(1) I feel tense or 'wound up':	Most of the time (3)<br>A lot of the time (2)<br>From time to time (1)<br>Not at all (0)
(2) I still enjoy the things I used to enjoy:	Definitely as much (0)<br>Not quite so much (1)<br>Only a little (2)<br>Hardly at all (3)
(3) I get a sort of frightened feeling as if something awful is about to happen:	Very definitely and quite badly (3)<br>Yes, but not too badly (2)<br>A little, but it doesn't worry me (1)<br>Not at all (0)
(4) I can laugh and see the funny side of things:	As much as I always could (0)<br>Not quite so much now (1)<br>Definitely not so much now (2)<br>Not at all (3)
(5) Worrying thoughts go through my mind:	A great deal of time (3)<br>A lot of the time (2)<br>From time to time, but not too often (1)<br>Only occasionally (0)
(6) I feel cheerful:	Not at all (3)<br>Not often (2)<br>Sometimes (1)<br>Most of the time (0)
(7) I can sit at ease and feel relaxed:	Definitely (0)<br>Usually (1)<br>Not often (2)<br>Not at all (3)
(8) I feel as I am slowed down:	Nearly all the time (3)<br>Very often (2)<br>Sometimes (1)<br>Not at all (0)
(9) I get sort of frightened feeling like 'butterflies' in the stomach:	Not at all (0)<br>Occasionally (1)<br>Quite often (2)<br>Very often (3)

Continued on next page

Table 3.1 – *Continued from previous page*

**Item**	**Reponse option (score)**
(10) I have lost my interest in my appearance:	Definitely (3)<br>I don't take as much care as I should (2)<br>I may not take quite as much care (1)<br>I take just as much care as ever (0)
(11) I feel restless as I have to be on the move:	Very much indeed (3)<br>Quite a lot (2)<br>Not very much (1)<br>Not at all (0)
(12) I look forward with enjoyment to things:	As much as I ever did (0)<br>Rather less than I used to (1)<br>Definitely less than I used to (2)<br>Hardly at all (3)
(13) I get sudden feelings of panic:	Very often indeed (3)<br>Quite often (2)<br>Not very often (1)<br>Not at all (0)
(14) I can enjoy a good book or radio or TV program:	Often (0)<br>Sometimes (1)<br>Not often (2)<br>Very seldom (3)

Adapted from *en.wikipedia.org/wiki/Hospital_Anxiety_and_Depression_Scale*.

3.2 Item response distribution

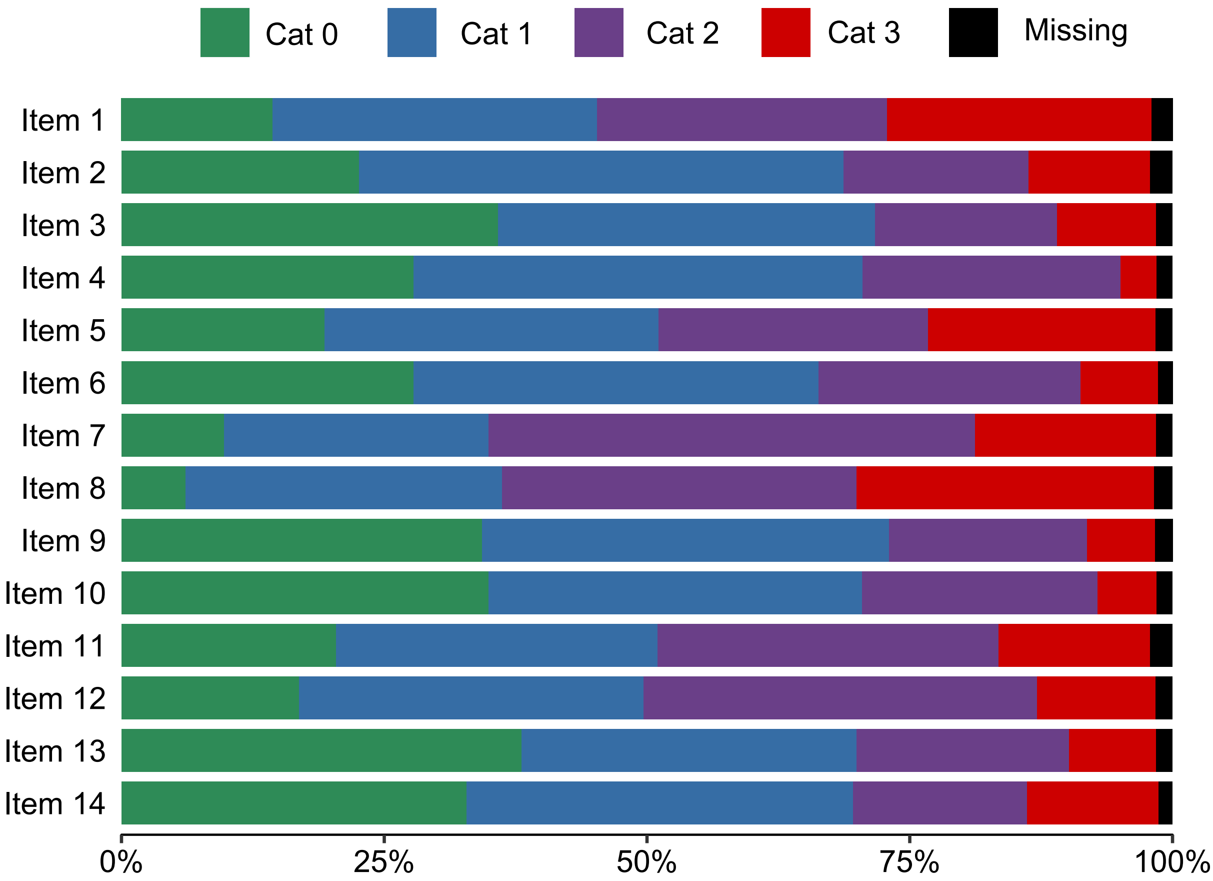

Figure 3.1: Relative item response frequencies (*n* = 35,908). Cat, category as defined in Table 3.1.

3.3 Model path diagrams

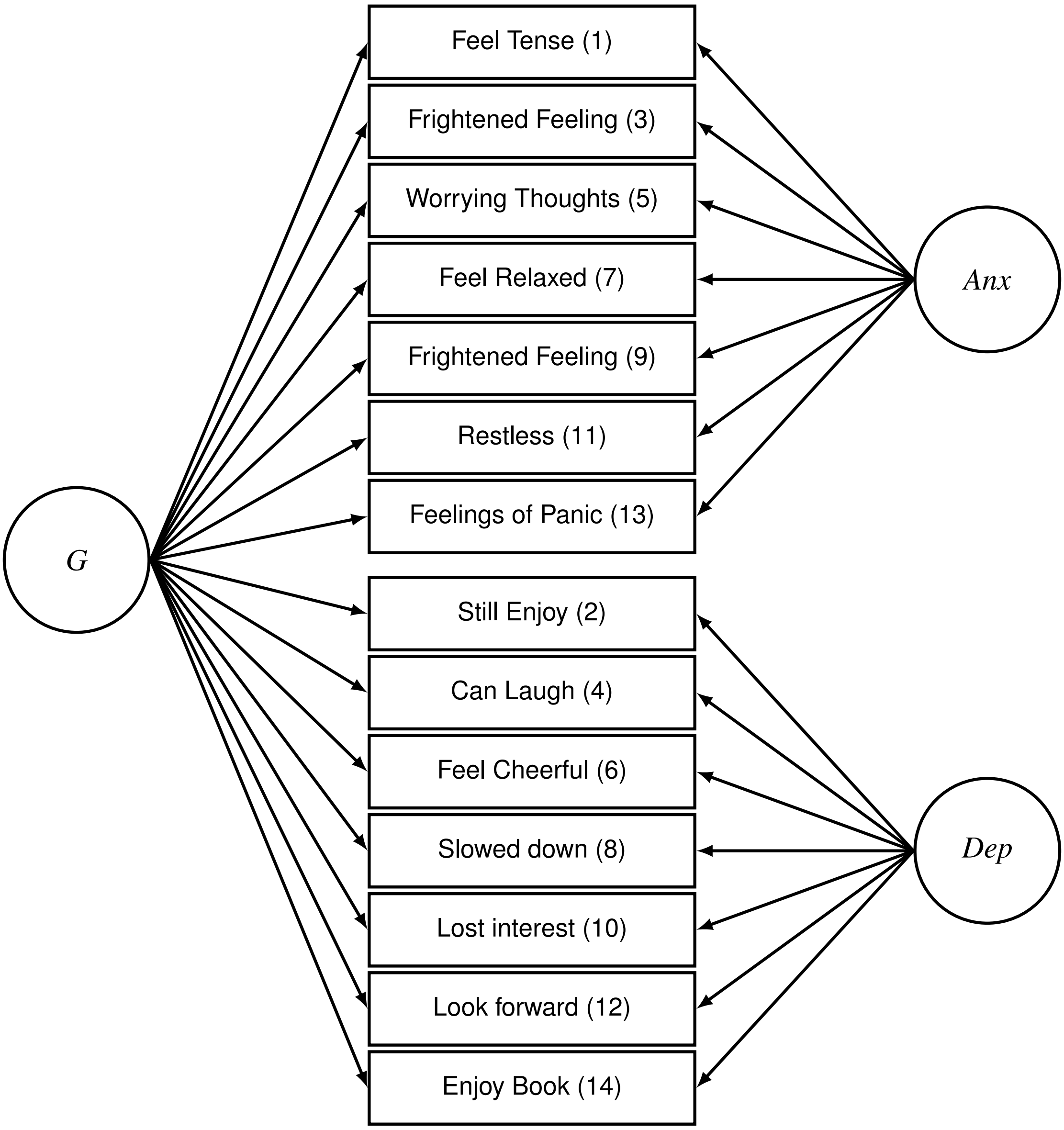

Figure 3.2: Path diagram of the final model. Factors, items and causal pathways are represented by circles, rectangles and arrows, respectively. Item number is presented within the parentheses.

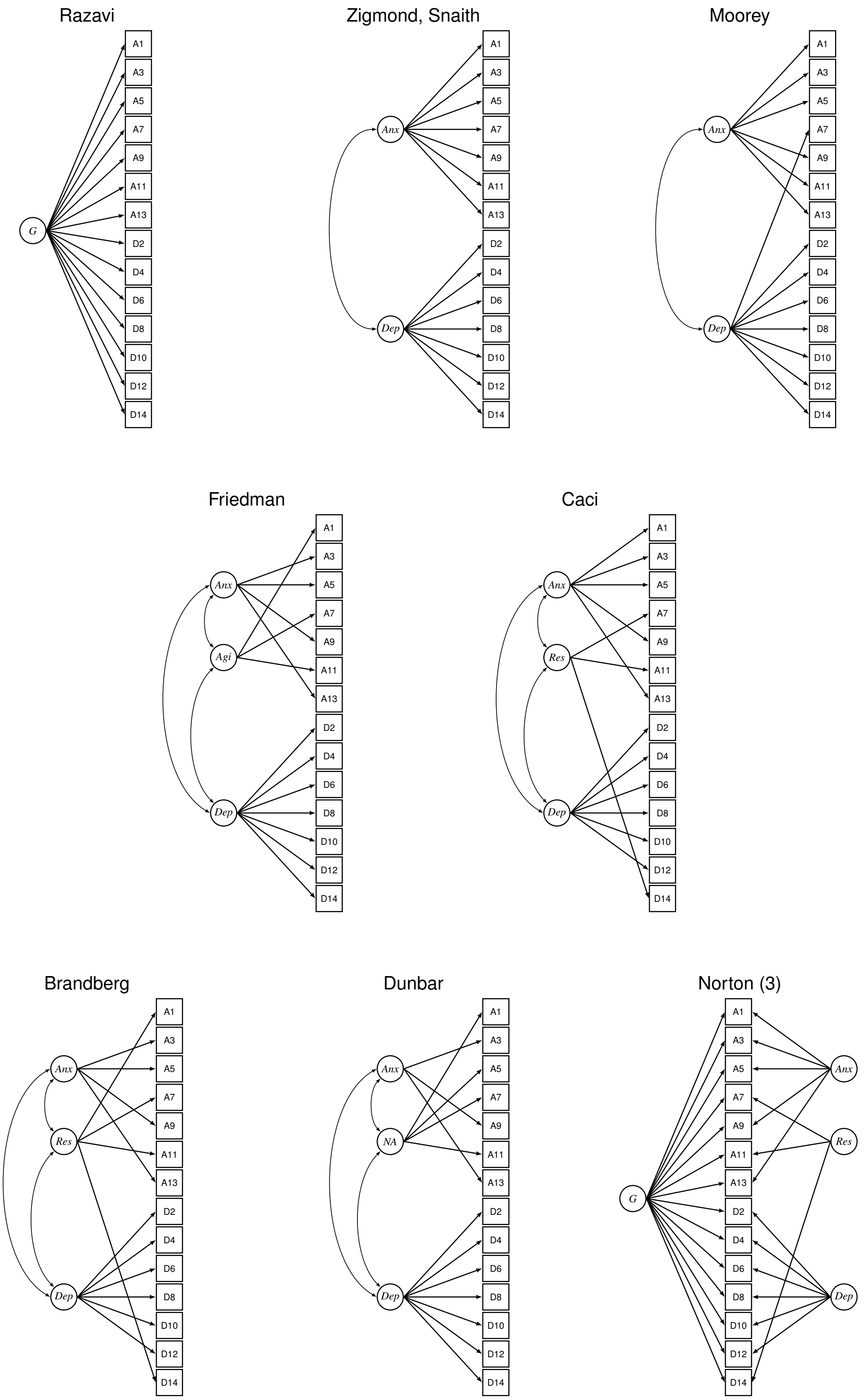

Figure 3.3: Path diagrams of computed models. Factors, items and causal pathways are represented by circles, squares and arrows, respectively.

3.4 Model selection

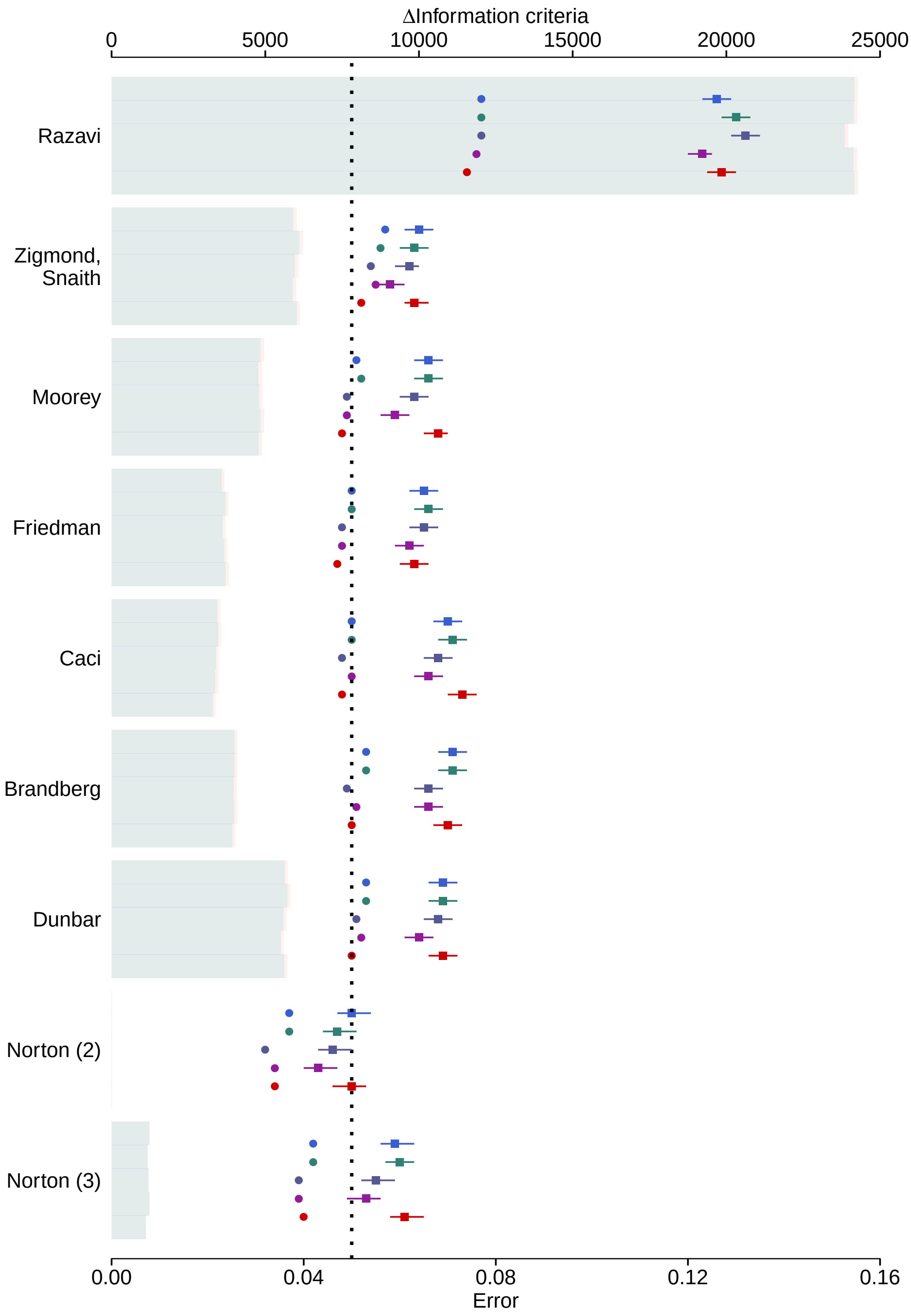

Figure 3.4: Global fit of the competing models. Squares (lines) and circles mark the point estimate (95% CI) of the root mean square error of approximation and standardized root mean square residual of the validation models (fold *k*), respectively. Meanwhile, green and red bars denote the difference in Akaike's information criterion and Schwartz's bayesian information criterion, relative to the best fitting model.

3.5 Final model

3.5.1 Theta distribution proxies

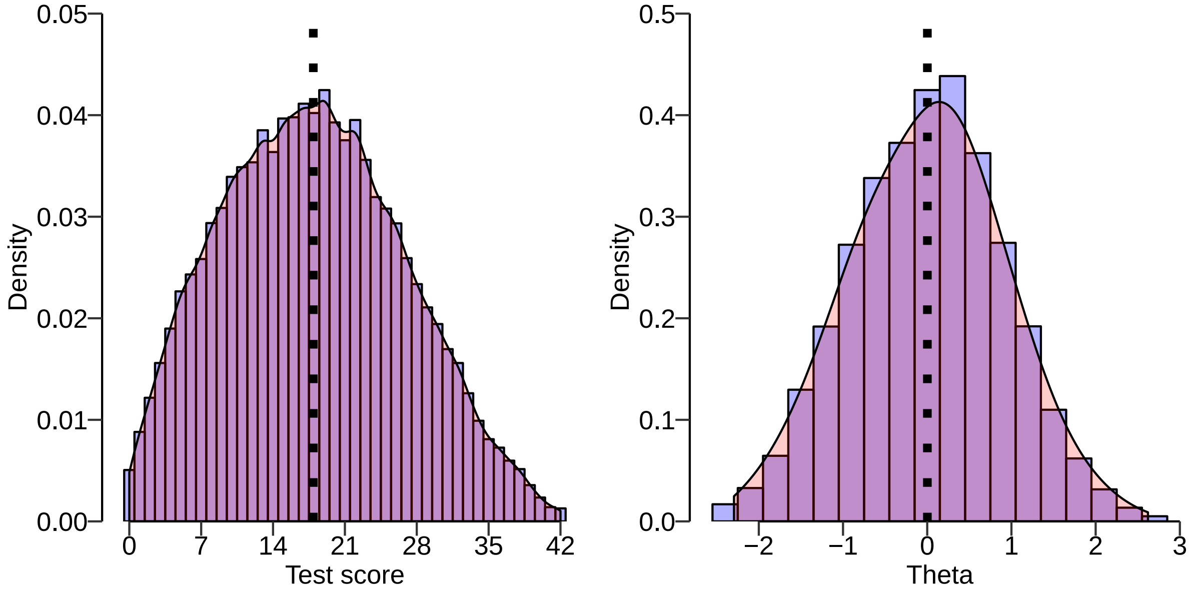

Figure 3.5: Hospital Anxiety and Depression theta distribution proxies. Histogram with overlayed density of test score (left) and estimated theta (right). Dotted vertical line shows the mean.

3.5.2 Item residual correlations

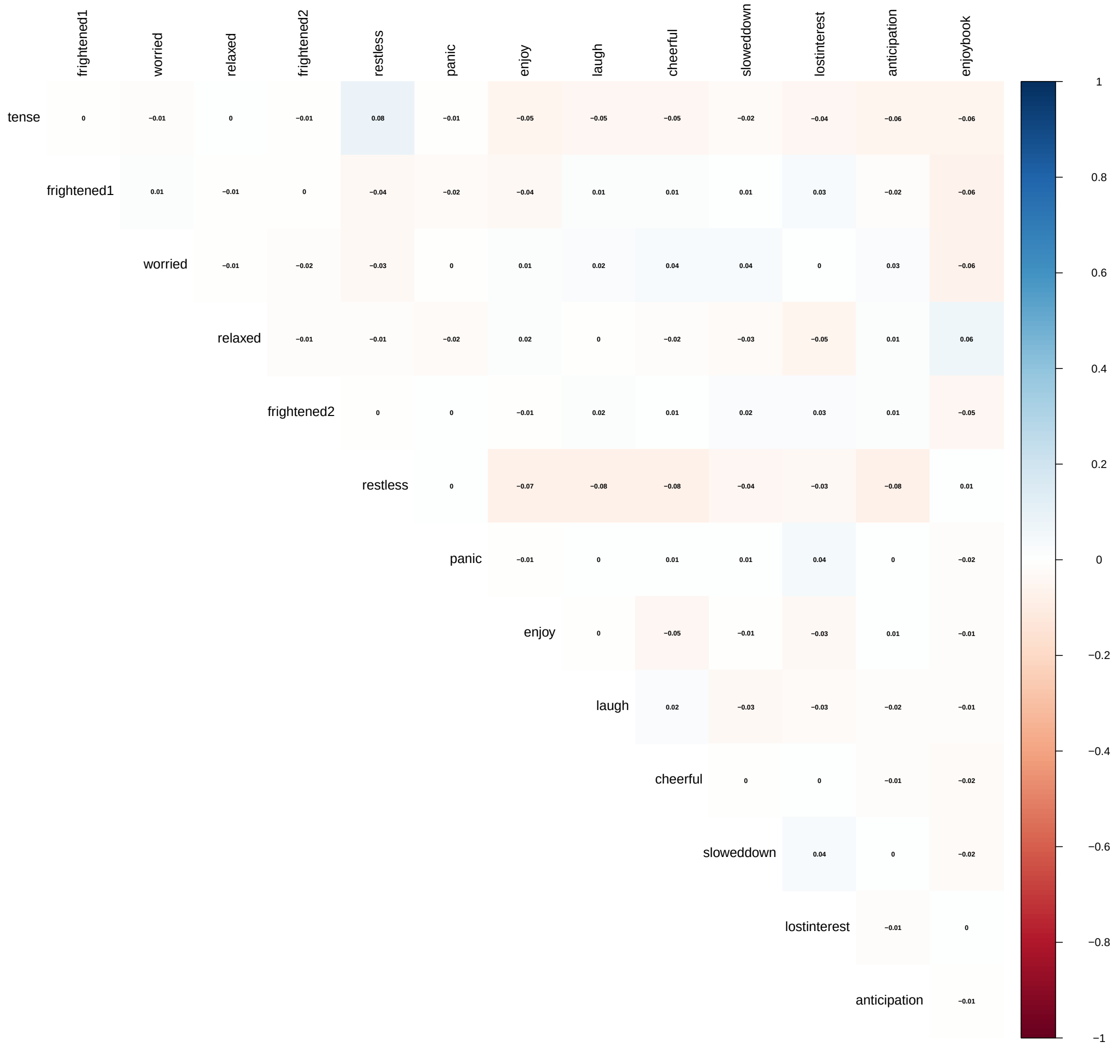

Figure 3.6: M_2^*-based item residuals.

3.5.3 Observed and expected item response proportions

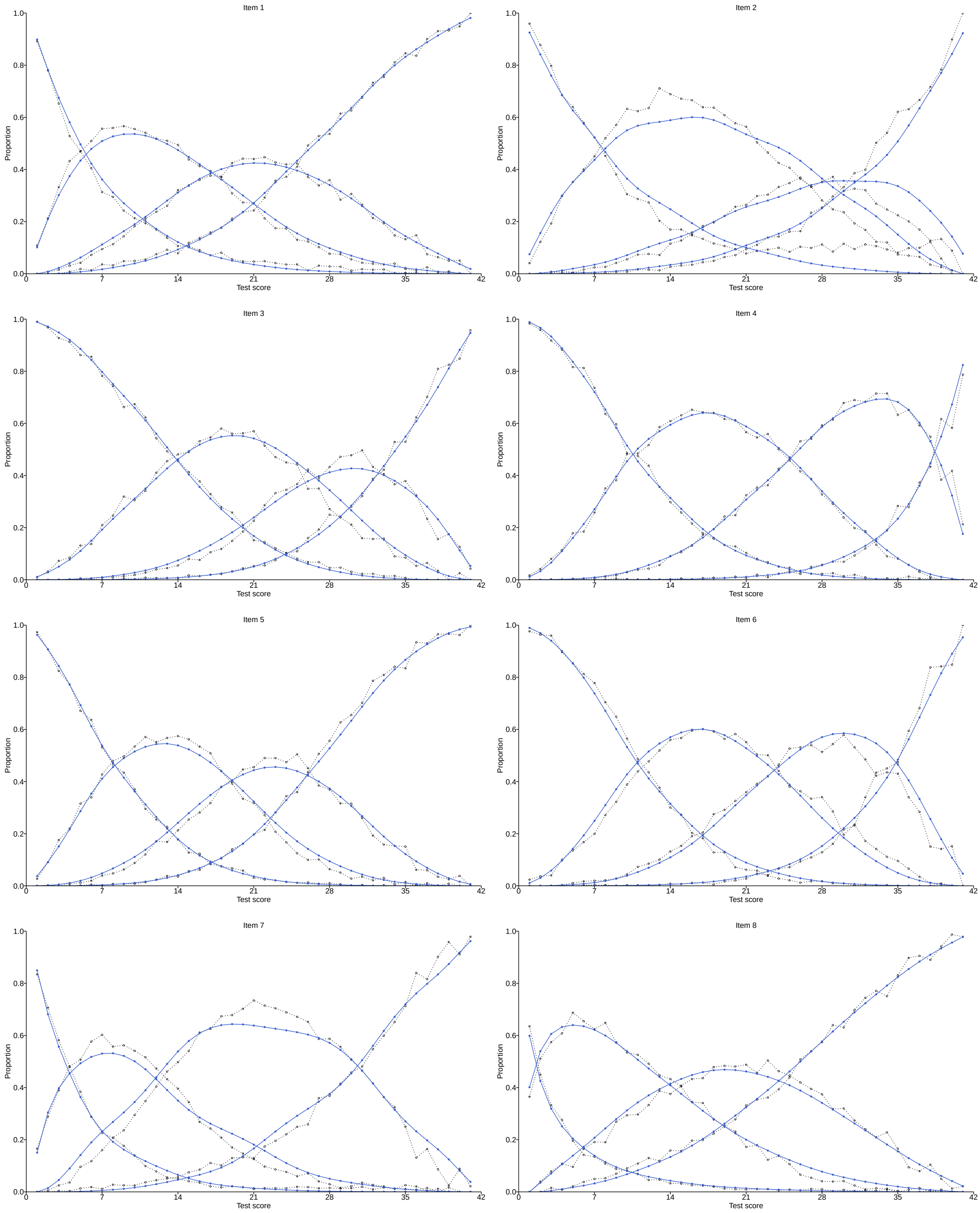

Figure 3.7: Observed and expected S-X^2-based proportions per category for items 1-8.

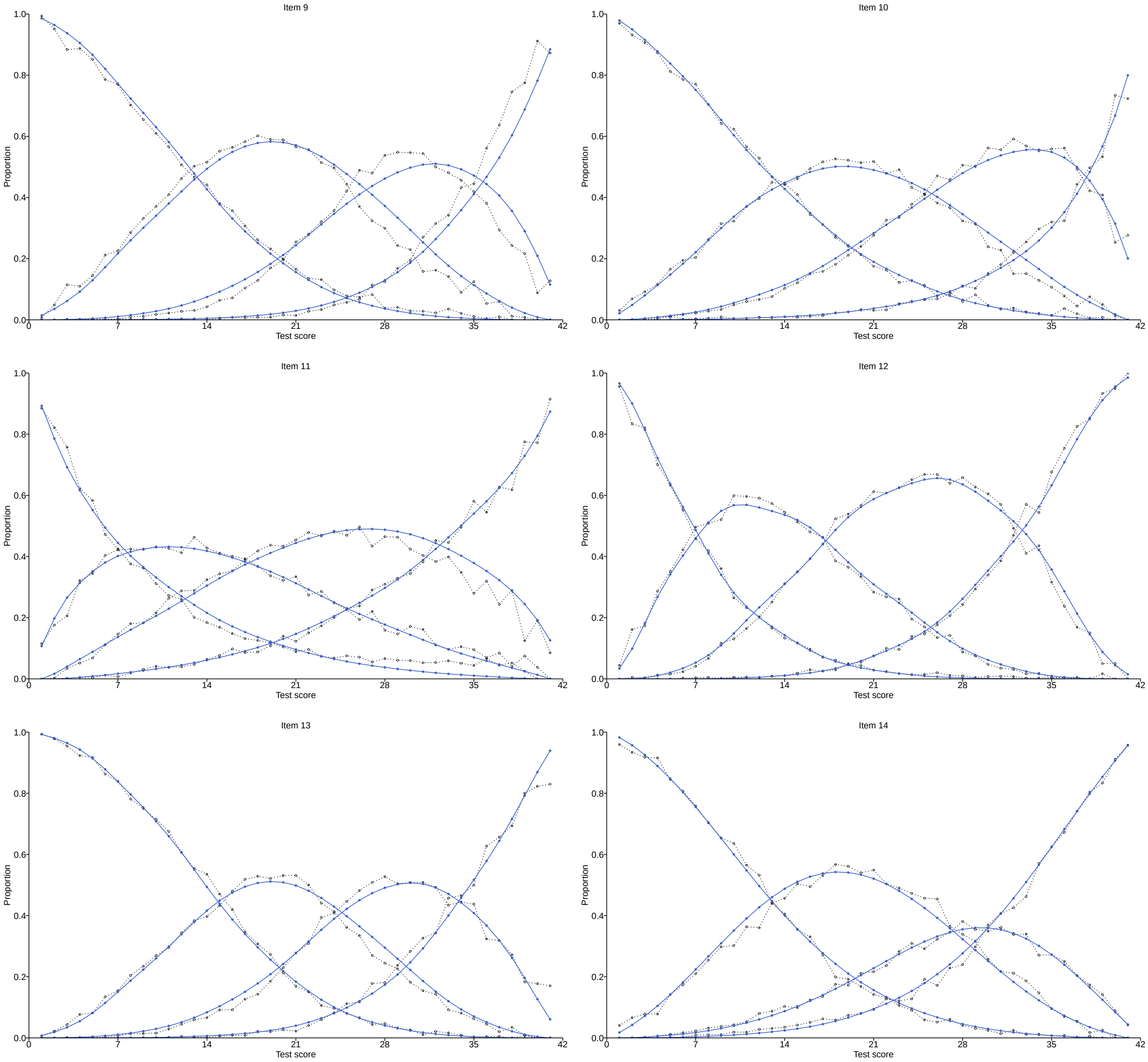

Figure 3.8: Observed and expected S-X^2-based proportions per category for items 9-14.

3.5.4 Marginal item category proportions

Table 3.2: Marginal item category response proportions based on S-X^2 frequencies.

	Observed (expected) proportions			
	Cat 0	Cat 1	Cat 2	Cat 3
Item 1	0.152 (0.153)	0.248 (0.245)	0.224 (0.229)	0.375 (0.373)
Item 2	0.237 (0.218)	0.360 (0.367)	0.172 (0.195)	0.231 (0.221)
Item 3	0.324 (0.322)	0.273 (0.279)	0.191 (0.192)	0.211 (0.207)
Item 4	0.268 (0.267)	0.321 (0.324)	0.305 (0.303)	0.106 (0.107)
Item 5	0.201 (0.201)	0.236 (0.237)	0.205 (0.210)	0.358 (0.353)
Item 6	0.269 (0.270)	0.292 (0.285)	0.249 (0.260)	0.190 (0.185)
Item 7	0.115 (0.111)	0.211 (0.210)	0.377 (0.386)	0.297 (0.293)
Item 8	0.070 (0.069)	0.258 (0.263)	0.273 (0.273)	0.398 (0.394)
Item 9	0.312 (0.311)	0.294 (0.302)	0.217 (0.228)	0.176 (0.159)
Item 10	0.319 (0.318)	0.279 (0.283)	0.268 (0.267)	0.133 (0.132)
Item 11	0.207 (0.199)	0.253 (0.247)	0.298 (0.311)	0.242 (0.242)
Item 12	0.180 (0.181)	0.249 (0.247)	0.327 (0.331)	0.245 (0.241)
Item 13	0.342 (0.340)	0.239 (0.244)	0.226 (0.224)	0.194 (0.193)
Item 14	0.303 (0.305)	0.285 (0.284)	0.170 (0.168)	0.242 (0.244)

3.5.5 Person fit

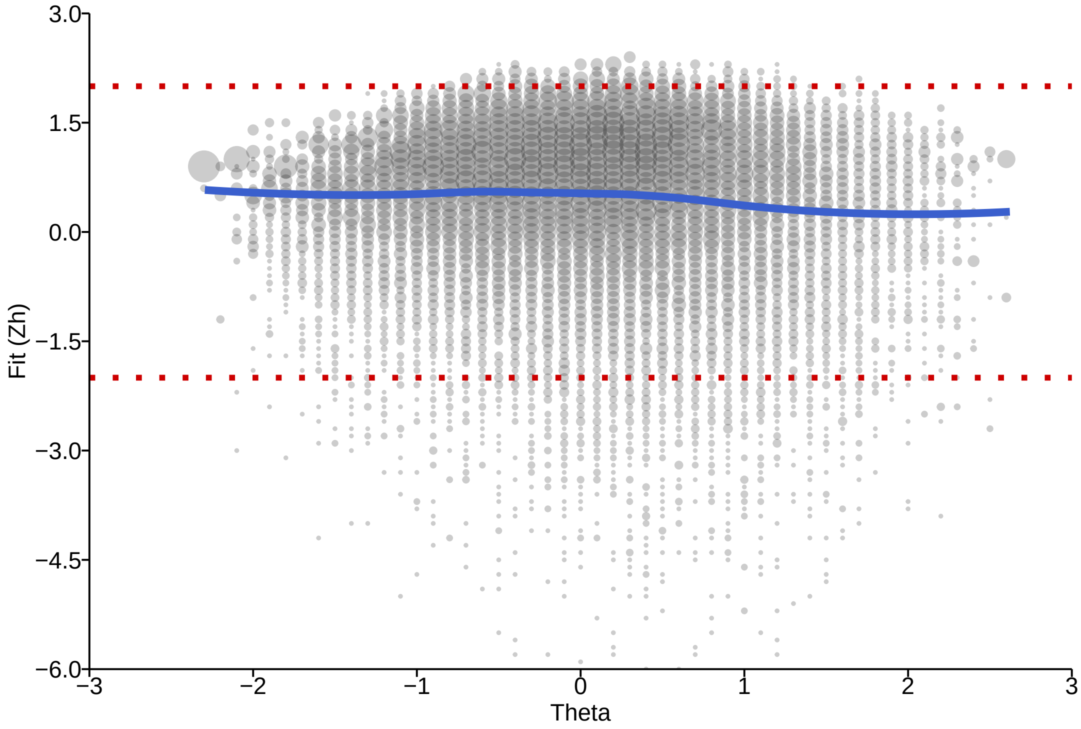

Figure 3.9: Person fit based on Zh. Bubbles depict estimates with increased size and darkness showing higher density. The trend is represented by the fitted line from a generalized additive model with smoothed integration. The dotted red lines mark ± 2.

3.6 Parameter estimate robustness

3.6.1 Item parameters

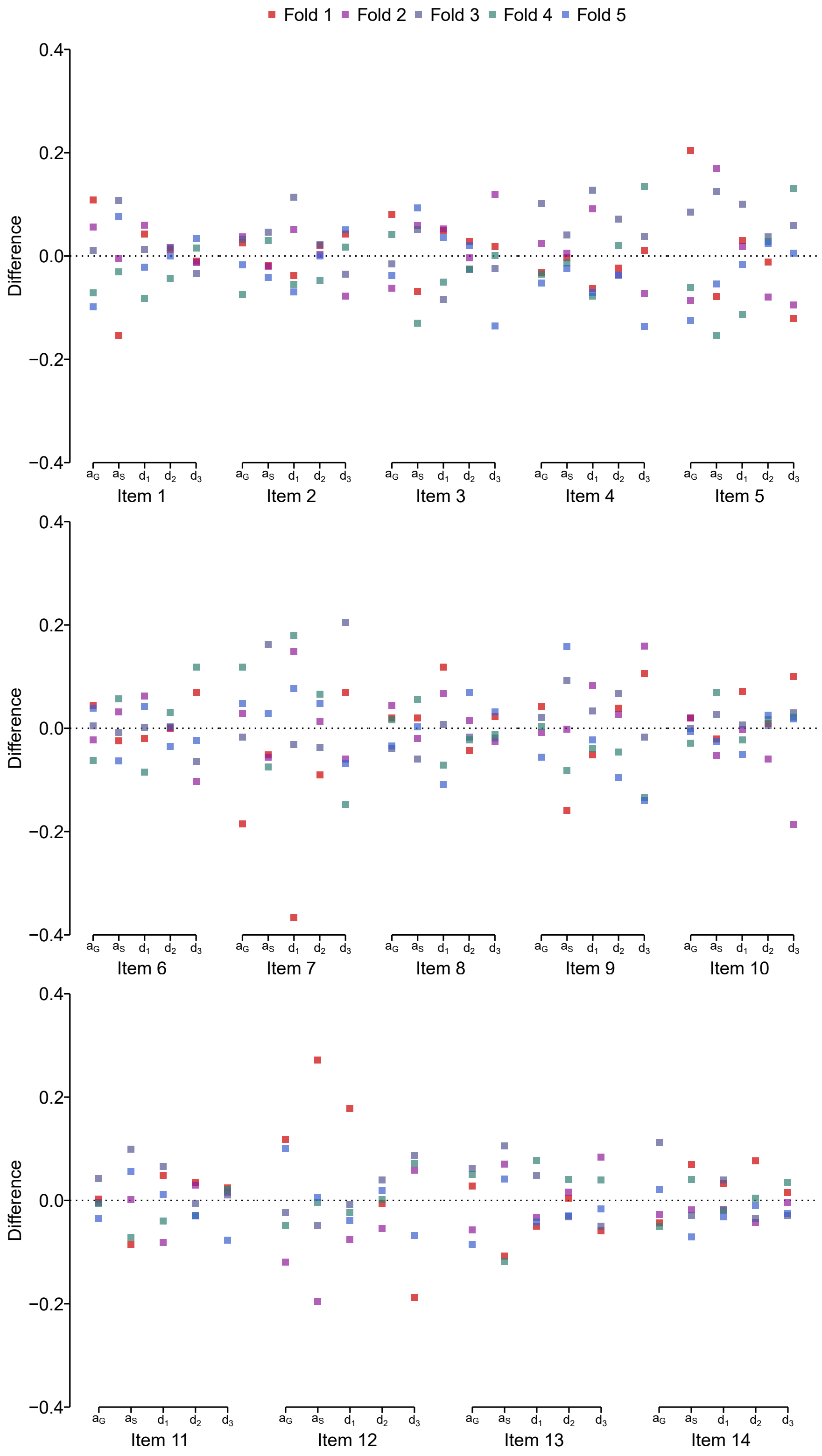

Figure 3.10: Parameter estimate difference between training (excluding fold k) and validation (fold k) models.

3.6.2 Person estimates

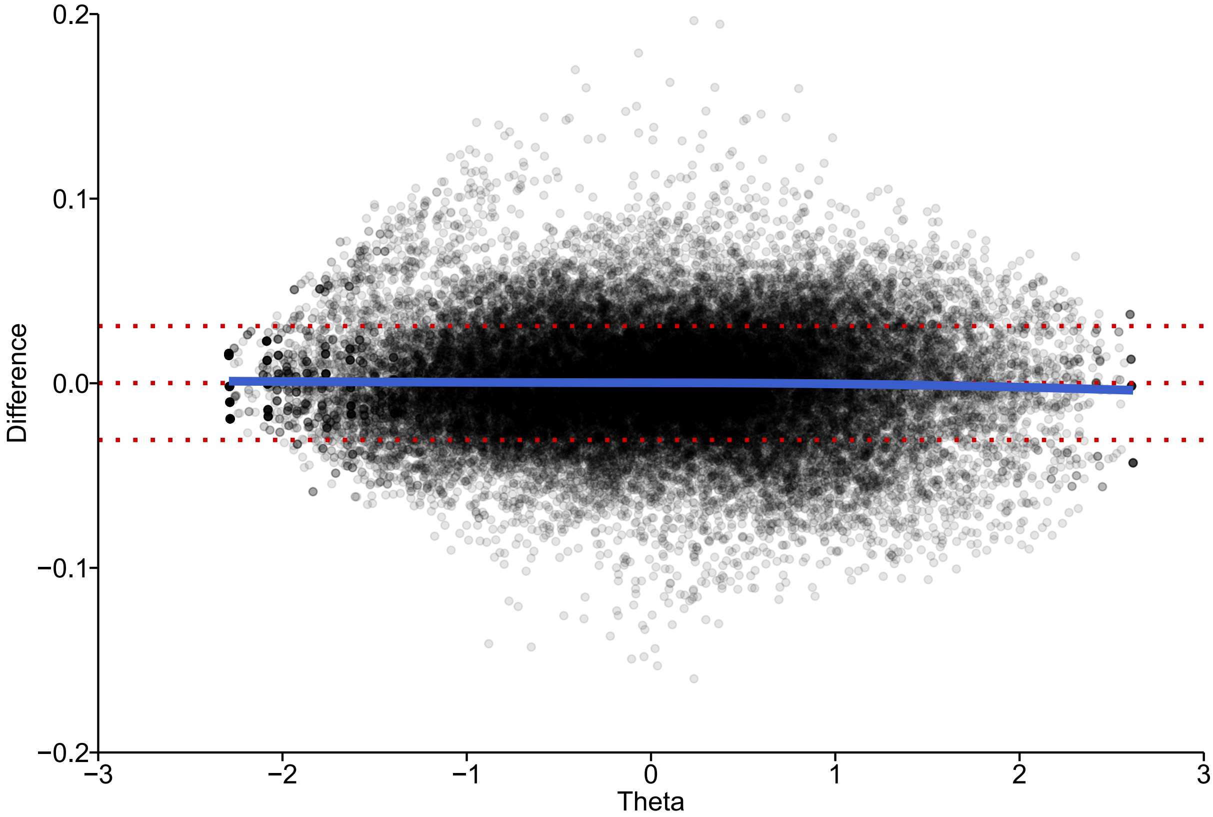

Figure 3.11: Difference in theta estimates between the training (excluding fold *k*) and validation (fold *k*) models. The sample trend is depicted by a fitted generalized additive model line with smoothed integration (blue line). Points show individual observations, with darker areas indicating higher density. Red dotted lines mark mean and one standard deviation.

4. R scripts

4.1 Install CRAN packages

```r
install.packages(c('mirt','lavaan','scales'),dependencies=TRUE)
```

4.2 Analysis scripts

```r
#0. @@@@ Load mirt package for multidimensional item response theory @@@@
library(mirt)
citation('mirt')
```

```r
#1. @@@@ Randomly split the data into k folds @@@@

## function to create folds ----
kfold <- function(data,k){

  # Set random seed for reproducibility
  seed <- sample.int(n=1000000,size=1)
  set.seed(seed)

  # Shuffle the order of the rows
  shuffle <- sample(1:nrow(data))

  # Split the shuffled dataset into k groups
  fold <- as.numeric(cut(shuffle,
                         breaks=quantile(1:length(shuffle),
                                         probs=seq(0,1,by=1/k),na.rm=T),
                         include.lowest=T))

  # Export
  return(list('fold'=fold,
              'seed'=seed))

}

## Create folds ----
fold <- kfold(data,k=5)
```

```r
#2. @@@@ Fit the k-fold cross-validation models @@@@

## mirt wrapper function ----
cvalmirt <- function(data=NULL,fold=NULL,model=NULL,
                     iType=NULL,estimator='EM',
                     cval=c('train','fixed','free'),
                     pars=NULL,k){

  tryCatch(
    mirt(data=if(cval=='train'){data[fold!=k,]}else{data[fold==k,]},
         model=model,
         itemtype=iType,
         SE=ifelse(cval=='fixed',FALSE,TRUE),
         calcNull=TRUE,
         method=ifelse(cval=='fixed','EM',estimator),
         technical=list(
           NCYCLES=5000,
           theta_lim=c(-4,4),
           removeEmptyRows=TRUE
         ),
         TOL=switch(cval=='fixed',NaN),
         pars=if(cval=='fixed'){pars[[k]]}else{NULL}),
    error=function(e) NULL
  )

}


## bfactor wrapper function ----
cvalbfac <- function(data=NULL,fold=NULL,model=NULL,
                     cval=c('train','fixed','free'),
                     iType=NULL,spec=NULL,
                     pars=NULL,k){

  tryCatch(
    bfactor(data=if(cval=='train'){data[fold!=k,]}else{data[fold==k,]},
            model=spec,
            model2=model,
            itemtype=iType,
            SE=ifelse(cval=='fixed',F,T),
            calcNull = TRUE,
            technical=list(
              NCYCLES=5000,
              theta_lim=c(-4,4),
              removeEmptyRows=TRUE
            ),
            TOL = switch(cval=='fixed',NaN),
            pars=if(cval=='fixed'){pars[[k]]}else{NULL}),
    error=function(e) NULL
  )

}
```

```r
## Train mirt models on folds!=k ----
train <- lapply(1:k,function(x) cvalmirt(data,fold,model,
                                         cval='train',iType,
                                         k=x))

## Fit mirt models on folds==k, using the parameter estimates from training models ----
pars <- lapply(1:k,function(x) mod2values(train[[x]]))
fixed <- lapply(1:k,function(x) cvalmirt(data,fold,model,
                                         cval='fixed',iType,
                                         k=x))

## Train mirt models on folds==k ----
free <- lapply(1:k,function(x) cvalmirt(data,fold,model,
                                        cval='free',iType,
                                        k=x))
```

```r
#3. @@@@ Model fit @@@@

## Convergence information, AIC, BIC ----
train[[1]]

## RMSEA, SRMSR, CFI, TLI ----
M2(fixed[[1]],type='M2*',QMC=FALSE)

## Item residuals ----
M2(fixed[[1]],QMC=FALSE,residmat=TRUE)

## Observed and expected S_X2 frequencies ----
itemfit(fixed[[1]],S_X2.tables=TRUE,QMC=FALSE)

## Person fit statistics (Zh) ----
personfit(fixed[[1]],QMC=FALSE)
```

```r
#4. @@@@ Parameter estimates @@@@

## Standardized coefficients (Loadings, Communalities/R2) ----
summary(fixed[[1]])

## Unstandardized coefficients (Threshold, Slopes/Discrimination) ----
coef(fixed[[1]],simplify=TRUE)

## Multidimensional estimates ----
MDISC(fixed[[1]])
MDIFF(fixed[[1]])

## Person estimates (Theta/Factor scores)
fscores(fixed[[1]],
        method='EAP',
        full.scores=TRUE,
        full.scores.SE=TRUE,
        QMC=FALSE)
```

4.3 Model specifications

```r
#1. @@@@ SF-36 @@@@

## Unidimensional model ----
model <- '
  G = bp1,bp2,gh1,gh2,gh3,gh4,gh5,mh1,mh2,mh3,mh4,mh5,pf1,pf2,pf3,pf4,pf5,pf6,pf7,pf8,pf9,pf10,
  re1,re2,re3,rp1,rp2,rp3,rp4,sf1,sf2,vt1,vt2,vt3,vt4
'

## Correlated-traits model with eight factors ----
model <- '
  BP = bp1,bp2
  GH = gh1,gh2,gh3,gh4,gh5
  MH = mh1,mh2,mh3,mh4,mh5
  PF = pf1,pf2,pf3,pf4,pf5,pf6,pf7,pf8,pf9,pf10
  RE = re1,re2,re3
  RP = rp1,rp2,rp3,rp4
  SF = sf1,sf2
  VT = vt1,vt2,vt3,vt4
  CONSTRAIN = (19,31,a7),(20,21,a1)
  COV = BP*GH*MH*PF*RE*RP*SF*VT
'

## Correlated-traits model with two factors ----
model <- '
  G1 = bp1,bp2,gh1,gh2,gh3,gh4,gh5,mh1,mh2,mh3,mh4,mh5,pf1,pf2,pf3,pf4,pf5,pf6,pf7,pf8,pf9,pf10,
  re1,re2,re3,rp1,rp2,rp3,rp4,sf1,sf2,vt1,vt2,vt3,vt4
  G2 = bp1,bp2,gh1,gh2,gh3,gh4,gh5,mh1,mh2,mh3,mh4,mh5,pf1,pf2,pf3,pf4,pf5,pf6,pf7,pf8,pf9,pf10,
  re1,re2,re3,rp1,rp2,rp3,rp4,sf1,sf2,vt1,vt2,vt3,vt4
  COV = G1*G2 #Remove this row for testing orthogonal factors
'

## Bifactor model with eight specific factors ----
model <- '
  G = bp1,bp2,gh1,gh2,gh3,gh4,gh5,mh1,mh2,mh3,mh4,mh5,pf1,pf2,pf3,pf4,pf5,pf6,pf7,pf8,pf9,pf10,
  re1,re2,re3,rp1,rp2,rp3,rp4,sf1,sf2,vt1,vt2,vt3,vt4
  S1 = gh1,gh2,gh3,gh4,gh5
  S2 = pf1,pf2,pf3,pf4,pf5,pf6,pf7,pf8,pf9,pf10
  S3 = rp1,rp2,rp3,rp4
  S4 = re1,re2,re3
  S5 = sf1,sf2
  S6 = bp1,bp2
  S7 = vt1,vt2,vt3,vt4
  S8 = mh1,mh2,mh3,mh4,mh5
  CONSTRAIN = (19,31,a6),(20,21,a7)
'
spec <- c(1,2,2,2,2,2,2,2,2,2,2,3,3,3,3,4,4,4,5,6,6,7,8,8,8,7,8,7,8,7,5,1,1,1,1)
```

```r
## Two-tier bifactor model with eight specific factors (1) ----
model <- '
  G1 = pf1,pf2,pf3,pf4,pf5,pf6,pf7,pf8,pf9,pf10,rp1,rp2,rp3,rp4,bp1,bp2,gh1,gh2,gh3,gh4,gh5
  G2 = mh1,mh2,mh3,mh4,mh5,re1,re2,re3,sf1,sf2,vt1,vt2,vt3,vt4
  S1 = gh1,gh2,gh3,gh4,gh5
  S2 = pf1,pf2,pf3,pf4,pf5,pf6,pf7,pf8,pf9,pf10
  S3 = rp1,rp2,rp3,rp4
  S4 = re1,re2,re3
  S5 = sf1,sf2
  S6 = bp1,bp2
  S7 = vt1,vt2,vt3,vt4
  S8 = mh1,mh2,mh3,mh4,mh5
  CONSTRAIN = (19,31,a7),(20,21,a8)
  COV = G1*G2 #Remove this row for testing orthogonal factors
'
spec <- c(1,2,2,2,2,2,2,2,2,2,2,3,3,3,3,4,4,4,5,6,6,7,8,8,8,7,8,7,8,7,5,1,1,1,1)

## Two-tier bifactor model with eight specific factors (2) ----
model <- '
  G1 = pf1,pf2,pf3,pf4,pf5,pf6,pf7,pf8,pf9,pf10,rp1,rp2,rp3,rp4,bp1,bp2,gh1,gh2,gh3,gh4,gh5,
  vt1,vt2,vt3,vt4
  G2 = mh1,mh2,mh3,mh4,mh5,re1,re2,re3,sf1,sf2,vt1,vt2,vt3,vt4,gh1,gh2,gh3,gh4,gh5
  S1 = gh1,gh2,gh3,gh4,gh5
  S2 = pf1,pf2,pf3,pf4,pf5,pf6,pf7,pf8,pf9,pf10
  S3 = rp1,rp2,rp3,rp4
  S4 = re1,re2,re3
  S5 = sf1,sf2
  S6 = bp1,bp2
  S7 = vt1,vt2,vt3,vt4
  S8 = mh1,mh2,mh3,mh4,mh5
  CONSTRAIN = (19,31,a7),(20,21,a8)
  COV = G1*G2 #Remove this row for testing orthogonal factors
'
spec <- c(1,2,2,2,2,2,2,2,2,2,2,3,3,3,3,4,4,4,5,6,6,7,8,8,8,7,8,7,8,7,5,1,1,1,1)

## Two-tier bifactor model with eight specific factors (3) ----
ttm3 <- '
  G1 = bp1,bp2,gh1,gh2,gh3,gh4,gh5,mh1,mh2,mh3,mh4,mh5,pf1,pf2,pf3,pf4,pf5,pf6,pf7,pf8,pf9,pf10,
  re1,re2,re3,rp1,rp2,rp3,rp4,sf1,sf2,vt1,vt2,vt3,vt4
  G2 = bp1,bp2,gh1,gh2,gh3,gh4,gh5,mh1,mh2,mh3,mh4,mh5,pf1,pf2,pf3,pf4,pf5,pf6,pf7,pf8,pf9,pf10,
  re1,re2,re3,rp1,rp2,rp3,rp4,sf1,sf2,vt1,vt2,vt3,vt4
  S1 = gh1,gh2,gh3,gh4,gh5
  S2 = pf1,pf2,pf3,pf4,pf5,pf6,pf7,pf8,pf9,pf10
  S3 = rp1,rp2,rp3,rp4
  S4 = re1,re2,re3
  S5 = sf1,sf2
  S6 = bp1,bp2
  S7 = vt1,vt2,vt3,vt4
  S8 = mh1,mh2,mh3,mh4,mh5
  LBOUND = (1-35,a1,0.0),(1-35,a2,0.0),(1-35,a3,0.0),(1-35,a4,0.0),
  (1-35,a5,0.0),(1-35,a6,0.0),(1-35,a7,0.0),(1-35,a8,0.0),(1-35,a9,0.0),
  (1-35,a10,0.0) # Remove this row to allow negative values
  CONSTRAIN = (19,31,a7),(20,21,a8)
  COV = G1*G2 #Remove this row for testing orthogonal factors
'
spec <- c(1,2,2,2,2,2,2,2,2,2,2,3,3,3,3,4,4,4,5,6,6,7,8,8,8,7,8,7,8,7,5,1,1,1,1)
```

```r
## Unidimensional model (physical health part) ----
model <- '
  G = gh1,pf1,pf2,pf3,pf4,pf5,pf6,pf7,pf8,pf9,pf10,rp1,rp2,rp3,rp4,bp1,bp2,gh2,gh3,gh4,gh5
'

## Correlated-traits model with four factors (physical health part) ----
ct.pcs <- '
  BP = bp1,bp2
  GH = gh1,gh2,gh3,gh4,gh5
  PF = pf1,pf2,pf3,pf4,pf5,pf6,pf7,pf8,pf9,pf10
  RP = rp1,rp2,rp3,rp4
  CONSTRAIN = (16,17,a1)
  COV = BP*GH*PF*RP
'

## Bifactor model with four specific factors (physical health part) ----
model <- '
  G = bp1,bp2,gh1,gh2,gh3,gh4,gh5,pf1,pf2,pf3,pf4,pf5,pf6,pf7,pf8,pf9,pf10,rp1,rp2,rp3,rp4
  S1 = bp1,bp2
  S2 = gh1,gh2,gh3,gh4,gh5
  S3 = pf1,pf2,pf3,pf4,pf5,pf6,pf7,pf8,pf9,pf10
  S4 = rp1,rp2,rp3,rp4
  CONSTRAIN = (16,17,a2)
'
spec <- c(1,2,2,2,2,2,2,2,2,2,2,3,3,3,3,4,4,1,1,1,1)


## Unidimensional model (mental health part) ----
model <- '
  G = re1,re2,re3,sf1,vt1,mh1,mh2,mh3,vt2,mh4,vt3,mh5,vt4,sf2
'

##  Correlated-traits model with four factors (mental health part) ----
model <- '
  MH = mh1,mh2,mh3,mh4,mh5
  RE = re1,re2,re3
  SF = sf1,sf2
  VT = vt1,vt2,vt3,vt4
  CONSTRAIN = (4,14,a3)
  COV = MH*RE*SF*VT
'

## Bifactor model (mental health part) ----
model <- '
  G = mh1,mh2,mh3,mh4,mh5,re1,re2,re3,sf1,sf2,vt1,vt2,vt3,vt4
  S1 = mh1,mh2,mh3,mh4,mh5
  S2 = re1,re2,re3
  S3 = sf1,sf2
  S4 = vt1,vt2,vt3,vt4
  CONSTRAIN = (4,14,a4)
'
spec <- c(1,1,1,2,3,4,4,4,3,4,3,4,3,2)
```

```
#2. @@@@ EQ-5D @@@@

## Unidimensional model ----
model <- '
  G = mobility,selfcare,activities,pain,anxiety
'
```

```
#3. @@@@ Hospital Anxiety and Depression Scale @@@@

## Razavi ----
model <- '
  G = enjoy,laugh,cheerful,sloweddown,lostinterest,anticipation,enjoybook,
  tense,frightened1,worried,relaxed,frightened2,restless,panic
'

## Zigmond and Snaith ----
model <- '
  ANX = tense,frightened1,worried,relaxed,frightened2,restless,panic
  DEP = enjoy,laugh,cheerful,sloweddown,lostinterest,anticipation,enjoybook
  COV = ANX*DEP
'

## Moorey ----
model <- '
  ANX = tense,frightened1,worried,frightened2,restless,panic
  DEP = enjoy,laugh,cheerful,sloweddown,lostinterest,anticipation,enjoybook,relaxed
  COV = ANX*DEP
'

## Friedman ----
model <- '
  ANX = frightened1,worried,frightened2,panic
  AGI = tense,relaxed,restless
  DEP = enjoy,laugh,cheerful,sloweddown,lostinterest,anticipation,enjoybook
  COV = ANX*AGI*DEP
'

## Caci ----
model <- '
  ANX = tense,frightened1,worried,frightened2,panic
  RES = relaxed,restless,enjoybook
  DEP = enjoy,laugh,cheerful,sloweddown,lostinterest,anticipation
  COV = ANX*RES*DEP
'

## Brandberg ----
model <- '
  ANX = frightened1,worried,frightened2,panic
  RES = tense,relaxed,restless,enjoybook
  DEP = enjoy,laugh,cheerful,sloweddown,lostinterest,anticipation
  COV = ANX*RES*DEP
'
```

```
## Dunbar ----
model <- '
  ANX = frightened1,frightened2,panic
  NEA  = tense,worried,relaxed,restless
  DEP = enjoy,laugh,cheerful,sloweddown,lostinterest,anticipation,enjoybook
  COV = ANX*NEA*DEP
'

## Bifactor model with two specific factors ----
spec <- c(1,2,1,2,1,2,1,2,1,2,1,2,1,2)

## Bifactor model with three specific factors ----
spec <- c(1,2,1,2,1,2,3,2,1,2,3,2,1,3)
'
```

4.4 Limited-information reproduction of the final models

```
#1. @@@@ Load lavaan package for confirmatory factor analysis @@@@
library(lavaan)
citation('lavaan')
```

```
#2. @@@@ SF-36 @@@@

## Import data ----
load('sf36.rda')

## Specify model ----
model <- '
  G1 =~ bp1+bp2+gh1+gh2+gh3+gh4+gh5+mh1+mh2+mh3+mh4+mh5+pf1+pf2+pf3+pf4+pf5+pf6+pf7+
  pf8+pf9+pf10+re1+re2+re3+rp1+rp2+rp3+rp4+sf1+sf2+vt1+vt2+vt3+vt4
  G2 =~ bp1+bp2+gh1+gh2+gh3+gh4+gh5+mh1+mh2+mh3+mh4+mh5+pf1+pf2+pf3+pf4+pf5+pf6+pf7+
  pf8+pf9+pf10+re1+re2+re3+rp1+rp2+rp3+rp4+sf1+sf2+vt1+vt2+vt3+vt4

  BP =~ a*bp1+a*bp2
  GH =~ gh1+gh2+gh3+gh4+gh5
  MH =~ mh1+mh2+mh3+mh4+mh5
  PF =~ pf1+pf2+pf3+pf4+pf5+pf6+pf7+pf8+pf9+pf10
  RE =~ re1+re2+re3
  RP =~ rp1+rp2+rp3+rp4
  SF =~ b*sf1+b*sf2
  VT =~ vt1+vt2+vt3+vt4
'

## Compute model (based on Pearson correlation due to non-convergence when
## based on the polychoric correlation matrix) ----
fit <- cfa(model=model,
           sample.cov=sf36$data$pearson.cor,
           sample.nobs=sf36$data$sample.size,
           std.lv=TRUE,
           orthogonal=TRUE)
```

```r
# ##Should be more accurate, but is not currently supported by lavaan
# fit <- cfa(model=model,
#            ordered=c('bp1','bp2','gh1','gh2','gh3','gh4','gh5','mh1','mh2','mh3','mh4','mh5',
#                      'pf1','pf2','pf3','pf4','pf5','pf6','pf7','pf8','pf9','pf10','re1','re2',
#                      're3','rp1','rp2','rp3','rp4','sf1','sf2','vt1','vt2','vt3','vt4'),
#            sample.cov=sf36$data$poly.cor,
#            sample.nobs=sf36$data$sample.size,
#            WLS.V=sf36$data$weights,
#            NACOV=sf36$data$gamma,
#            estimator='WLSMV',
#            std.lv=TRUE)

## Results ----
fitMeasures(fit,c('cfi','tli','rmsea','srmr'))
lavInspect(fit,'std')$lambda
parameterEstimates(fit)
residuals(fit)
```

```r
#3. @@@@ EQ-5D @@@@

## Import data ----
load('eq5d.rda')

## Specify model ----
model <- '
  G =~ mobility+selfcare+activities+pain+anxiety
'

## Compute model ----
fit <- cfa(model=model,
           sample.cov=eq5d$data$poly.cor,
           sample.nobs=eq5d$data$sample.size,
           std.lv=TRUE)

## Should be more accurate, but is not currently supported by lavaan
# fit <- cfa(model=model,
#            ordered=c('mobility','selfcare','activities','pain','anxiety'),
#            sample.cov=eq5d$data$poly.cor,
#            sample.nobs=eq5d$data$sample.size,
#            WLS.V=eq5d$data$weights,
#            NACOV=eq5d$data$gamma,
#            estimator='WLSMV',
#            std.lv=TRUE)

## Results ----
fitMeasures( fit, c('cfi','tli','rmsea','srmr') )
lavInspect( fit, 'std' )$lambda
parameterEstimates(fit)
residuals(fit)
```

```r
#4. @@@@ Hospital Anxiety and Depression Scale @@@@

## Import data ----
load('hads.rda')

## Specify model ----
model <- '
  G =~ tense+frightened1+worried+relaxed+frightened2+restless+panic+
  enjoy+laugh+cheerful+sloweddown+lostinterest+anticipation+enjoybook
  anx =~ tense+frightened1+worried+frightened2+restless+panic
  dep =~ enjoy+laugh+cheerful+sloweddown+lostinterest+anticipation+enjoybook
'

## Compute model ----
fit <- cfa(model=model,
           sample.cov=hads$data$poly.cor,
           sample.nobs=hads$data$sample.size,
           orthogonal=TRUE,
           std.lv=TRUE)

# ##Should be more accurate, but is not currently supported by lavaan
# fit <- cfa(model=model,
#            ordered=c('tense','frightened1','worried','relaxed','frightened2','restless',
#                      'panic','enjoy','laugh','cheerful','sloweddown','lostinterest',
#                      'anticipation', 'enjoybook'),
#            sample.cov=hads$data$poly.cor,
#            sample.nobs=hads$data$sample.size,
#            WLS.V=hads$data$weights,
#            NACOV=hads$data$gamma,
#            estimator='WLSMV',
#            orthogonal=TRUE,
#            std.lv=TRUE)

## Results ----
fitMeasures(fit,c('cfi','tli','rmsea','srmr'))
lavInspect(fit,'std')$lambda
parameterEstimates(fit)
residuals(fit)
```

4.5 Scoring

```
#1. @@@@ Load mirt package for factor scoring algorithms @@@@
library(mirt)
citation('mirt')
```

```
#2. @@@@ Load scales package for scaling factor scoring @@@@
library(scales)
citation('scales')
```

```
#3. @@@@ Import the model @@@@

## SF-36 ----
load('sf36.rda')

## EQ-5D ----
load('eq5d.rda')

## Hospital Anxiety and Depression Scale ----
load('hads.rda')
```

```
#4. @@@@ Create example data for HADS model @@@@
DATA <- matrix(sample(c(0:3),2800,T),ncol=14)
```

```
#5. @@@@ Estimate the factor scores @@@@

## Function to estimate the scores ----
score <- function(Q=c('sf36','eq5d','hads'),data=DATA,scorerange=NULL){

  #Prepare data
  if(Q=='sf36'){
    model <- sf36$results$final.model$model
    colnames(data) <- colnames(sf36$data$poly.cor)
    SF36PCS <- c(-2.9049853,3.820332)
    SF36MCS <- c(-2.662892,4.0759777)

    #Weight pattern
    # rbind(c(4,2,2,2,2,2,2,2,2,0,2,1,1,1,1,0,0,0,4,5,4,5,0,0,0,0,0,0,0,0,4,0,4,0,4), #PCS max
    #       c(0,0,0,0,0,0,0,0,0,0,0,0,0,0,1,0,0,0,0,0,5,0,5,0,5,0,5,0,0,0,0,0,0), #PCS min
    #       c(4,0,0,0,0,2,0,2,2,2,0,1,1,1,0,1,1,1,4,5,4,5,5,5,5,5,5,5,5,5,4,4,4,4,4), #MCS max
    #       c(0,2,2,2,2,2,2,2,0,2,2,1,0,1,0,0,0,0,0,5,0,0,0,0,0,0,0,5,0,5,0,4,0,4,0)) #MCS min

  } else if(Q=='eq5d'){
    model <- eq5d$results$final.model$model
    colnames(data) <- colnames(eq5d$data$poly.cor)
    EQ5D <- c(-2.194757,2.796851)

  } else{
    model <- hads$results$final.model$model
    colnames(data) <- colnames(hads$data$poly.cor)
    HADS <- c(-2.494035,2.80567)
  }
```

```r
#Factor scores
  fs <- fscores(object=model,
                method=ifelse(Q=='sf36','MAP','EAP'),
                full.scores=TRUE,
                full.scores.SE=FALSE,
                append_response.pattern=FALSE,
                QMC=ifelse(Q=='sf36',TRUE,FALSE),
                response.pattern=data)

  #Scale factor scores
  if(is.numeric(scorerange)){
    if(Q=='sf36'){
      fs <- cbind(rescale(x=fs[,1],
                          to=scorerange,
                          from=SF36PCS),
                  rescale(fs[,2],
                          to=scorerange,
                          from=SF36MCS))
    } else {
      fs <- matrix(rescale(x=fs[,1],
                           to=scorerange,
                           from=if(Q=='eq5d'){EQ5D}else{HADS}))
    }
  }

  #Name factor scores
  if(Q=='sf36'){
    fs <- round(fs[,1:2],3)
    colnames(fs) <- c('PCS','MCS')
  } else{
    fs <- round(as.matrix(fs[,1]),3)
    colnames(fs) <- ifelse(Q=='eq5d','HRQoL','ED')
  }

  #Export results
  return(fs)

}


## Estimate scores ----
SCORE <- score(Q=c('sf36','eq5d','hads'),
               data=DATA,
               scorerange=c(0,100))
```
